# Advances in 3D Bioprinting for Breast Cancer Modeling: Tumor Microenvironment, Metastasis, and Drug Screening

**DOI:** 10.1002/adhm.71734

**Published:** 2026-10-05

**Authors:** Mustafa Nakipoglu, Mahboobeh Mahmoodi, Ehsanul Hoque Apu, Fahimeh Shahabipour, Sibel Emir Diltemiz, Sanjib Saha, Halima Alem, Nureddin Ashammakhi

**Affiliations:** ^1^ Department of Molecular Biology and Genetics Faculty of Sciences Bartın University Bartın Turkiye; ^2^ Department of Bioengineering Samueli School of Engineering University of California Los Angeles California USA; ^3^ Department of Biomedical Engineering Ya. C. Islamic Azad University Yazd Iran; ^4^ Joint Reconstruction Research Center Tehran University of Medical Sciences Tehran Iran; ^5^ Department of Biomedical Sciences Northeast Ohio Medical University Rootstown Ohio USA; ^6^ 3D Tissue Bioprinting Laboratory Division of Preclinical Innovation, National Center for Advancing Translational Sciences (NCATS), National Institute of Health (NIH) Rockville, MD USA; ^7^ Orthopedic and Trauma Research Center Mashhad University of Medical Sciences Mashhad Iran; ^8^ Department of Chemistry Eskisehir Technical University Eskisehir Turkiye; ^9^ Department of Pharmaceutical Sciences and Experimental Therapeutics College of Pharmacy University of Iowa Iowa City Iowa USA; ^10^ CNRS, IJL, Nancy Université de Lorraine France; ^11^ Institut Universitaire de France Paris France; ^12^ Institute For Quantitative Health Science & Engineering Michigan State University East Lansing Michigan USA

**Keywords:** 3D bioprinting, breast cancer, drug screening, in vitro models, metastasis, tumor microenvironment

## Abstract

The tumor microenvironment (TME) is a complex and heterogeneous milieu encompassing biological and biochemical events and diverse physical, chemical, and mechanical interactions between tumor and stromal cells. This heterogeneity plays a pivotal role in regulating tumor progression, metastasis, and resistance to therapy. In breast cancer, cancer‐associated fibroblasts, adipocytes, endothelial cells, and immune cells all contribute to this intricate environment, necessitating precise spatial and cellular modeling to better replicate physiological conditions. Moreover, angiogenesis and metastasis are key contributors to breast cancer mortality. Therefore, three‐dimensional (3D) models that incorporate co‐cultures of cancer and stromal cells are essential for recreating realistic TMEs. This review highlights the advances in 3D bioprinting technologies for modeling the breast cancer TME. We discuss their potential in drug screening, explore their advantages and limitations, and outline future directions to enhance their fidelity and translational relevance.

## Graphical abstract

Three‐dimensional bioprinting transforms breast cancer modeling from simple constructs into spatially organized, multicellular, perfused, and patient‐derived platforms. This review shows how bioinks, cellular composition, architecture, and flow govern tumor behavior and drug response, while revealing why complexity alone is insufficient without reproducibility, functional immune–vascular–lymphatic integration, and validation against patient outcomes.

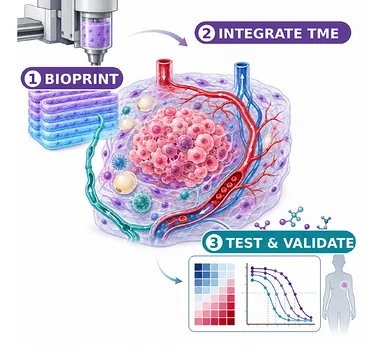

**Abbreviations**
3DBP: three‐dimensional bioprinting
5‐ARU: 5‐amino‐6‐D‐ribitylaminouracil
7‐AAD: 7‐aminoactinomycin D
ABCB1: ATP‐binding cassette subfamily B member 1
ABCC2: ATP‐binding cassette subfamily C member 2
ABCG2: ATP‐binding cassette subfamily G member 2
ADMSC: adipose‐derived mesenchymal stromal cell
ADSC/ASC: adipose‐derived stromal cell
Akt: protein kinase B
ALDH1: aldehyde dehydrogenase 1
AuNR: gold nanorod
BCN: bicyclo[6.1.0]nonyne
CAF: cancer‐associated fibroblast
CAP: cold atmospheric plasma
CAR‐T: chimeric antigen receptor T cell
CCL2: C‐C motif chemokine ligand 2
CCL21: C‐C motif chemokine ligand 21
CD24: cluster of differentiation 24
CD31: cluster of differentiation 31
CD44: cluster of differentiation 44
CD8: cluster of differentiation 8
CDH2: cadherin 2
CDK4/6: cyclin‐dependent kinases 4 and 6
CH: collagen–hyaluronic acid–poly(N‐isopropylacrylamide) formulation
CK5: cytokeratin 5
c‐Met: hepatocyte growth factor receptor
COL1A1: collagen type I alpha 1 chain
ColMA: methacrylated collagen
cRGD: cyclic Arg–Gly–Asp
CSC: cancer stem cell
DiI: 1,1′‐dioctadecyl‐3,3,3′,3′‐tetramethylindocarbocyanine dye
DLP: digital light processing
DYIGSR: Asp–Tyr–Ile–Gly–Ser–Arg
E‐cadherin: epithelial cadherin
EMT: epithelial–mesenchymal transition
ER: estrogen receptor
FAP: fibroblast activation protein
FAT‐ink: fragmented adipose tissue ink
FBS: fetal bovine serum
FITC: fluorescein isothiocyanate
GelMA: gelatin methacryloyl
GFOGER: Gly–Phe–Hyp–Gly–Glu–Arg
GFP: green fluorescent protein
HA: hyaluronic acid
HAMA: hyaluronic acid methacrylate
HA‐SH: thiolated hyaluronic acid
hBM‐MSC: human bone marrow‐derived mesenchymal stromal cell
HDF: human dermal fibroblast
HER2: human epidermal growth factor receptor 2
HGF: hepatocyte growth factor
HIF‐1α: hypoxia‐inducible factor 1 alpha
HLA: human leukocyte antigen
HPMEC: human pulmonary microvascular endothelial cell
HR: hormone receptor
HUVEC: human umbilical vein endothelial cell
IFN‐γ: interferon gamma
IL‐6: interleukin 6
IL‐8: interleukin 8
Ki‐67: Ki‐67 proliferation marker
LEC: lymphatic endothelial cell
MAIT: mucosal‐associated invariant T cell
MC: MCF‐7 monoculture
MCR: MCF‐7/MRC‐5 co‐culture
MCRT: transforming growth factor‐β‐treated MCF‐7/MRC‐5 co‐culture
MMP: matrix metalloproteinase
MMP2: matrix metalloproteinase 2
MSC: mesenchymal stromal cell
mTOR: mechanistic target of rapamycin
MUC1: mucin 1
N‐cadherin: neural cadherin
nHAp: nanohydroxyapatite
OoC: organ‐on‐a‐chip
PARP1: poly(ADP‐ribose) polymerase 1
PBMC: peripheral blood mononuclear cell
PDX: patient‐derived xenograft
PEG: poly(ethylene glycol)
PEGDA: poly(ethylene glycol) diacrylate
PEG‐DMA/PEGDM: poly(ethylene glycol) dimethacrylate
PI3K: phosphoinositide 3‐kinase
pNIPAM: poly(*N*‐isopropylacrylamide)
PODXL: podocalyxin‐like protein 1
PR: progesterone receptor
PTT: photothermal therapy
RDG: Arg–Asp–Gly
RGD: Arg–Gly–Asp
RuNO_2_TPyP: nitro‐ruthenium porphyrin complex {TPyP[Ru(NO_2_)(bpy)_2_]_4_}(PF_6_)_4_
S6: ribosomal protein S6
SLA: stereolithography
SLUG: snail family transcriptional repressor 2
SSEA‐4: stage‐specific embryonic antigen 4
STEM: synthetic tumor microenvironment mimic
TAF: tumor‐associated fibroblast
TGF‐β: transforming growth factor beta
Ti_3_C_2_T_x_
: surface‐terminated titanium carbide MXene
TME: tumor microenvironment
TNF‐α: tumor necrosis factor alpha
TWIST1: twist family basic helix–loop–helix transcription factor 1
VEGF: vascular endothelial growth factor
VPM: matrix metalloproteinase–degradable peptide crosslinker
α‐SMA: alpha‐smooth muscle actin
αvβ3: alpha‐v beta‐3 integrin

## 1 Introduction

Breast cancer is the second most commonly diagnosed cancer in women [1], with 2.3 million new cases and 670 000 deaths globally in 2022 [2]. It is expected that by 2050, new cases and related mortality will rise by 38% and 68%, respectively [2]. Treatment strategies depend on tumor stage, molecular subtype (e.g., hormone receptor status), and patient‐specific factors, and include surgery, radiation, and systemic therapy. While differences in the types and sensitivity to medications were demonstrated [3] and targeted therapies such as monoclonal antibodies have been developed [4, 5, 6], most preclinical studies rely on traditional two‐dimensional (2D) cell cultures [7] or animal models, which fail to recapitulate the complex human tumor microenvironment (TME).

Two‐dimensional (2D) cultures, though cost‐effective and widely used, lack key components of the TME [8] such as the extracellular matrix (ECM), stromal interactions, and mechanical cues [9, 10, 11]. Furthermore, 2D cell culture does not imitate the intercellular interactions and native structure of tumors [8], which are vital for cell differentiation, cell proliferation, cell migration, gene expression, protein production, enzymatic drug metabolism, and other important cellular functions [12, 13, 14]. These deficiencies impair their predictive value for drug screening, contributing to the high failure rate of anticancer drugs in clinical trials. Moreover, prolonged 2D culture often results in altered morphology, phenotypic drift, and lack of nutrient and metabolite gradients typical of in vivo tumors.

Transwell models have been developed, and allow limited investigation of cancer cell migration and invasion through porous polymeric membranes [15, 16]. In a migration assay, cancer cells are seeded on a porous membrane insert that allows measurement of cell translocation through pores in the membrane [16]. Invasion assays are relatively complex: a porous polymeric membrane is covered with a layer of ECM, and migration of cells through the ECM is measured [17, 18]. While useful for studying specific aspects of metastasis, they do not capture the cellular and structural complexity of solid tumors.

Animal models have been developed to recapitulate tumor pathophysiology [19, 20]. These include patient‐derived xenografts (PDX), which offer a more realistic three‐dimensional (3D) context [21]. However, species‐specific physiological differences limit their translational accuracy [22, 23], and ethical and cost considerations further complicate their use [24]. Thus, more physiologically relevant and human‐relevant models are urgently needed.

The breast cancer TME consists of a diverse array of tumor and stromal cells: fibroblasts, adipocytes, endothelial cells, immune cells, and mesenchymal stem cells (MSCs), which are embedded in a complex ECM [13, 14]. This dynamic microenvironment not only supports tumor growth but also promotes therapy resistance and metastatic dissemination through biochemical signaling and mechanical interactions [25, 26, 27]. Understanding the complexity of TME is critical for developing appropriate models and advancing drug development and personalized cancer therapy.

Therefore, the development of 3D microphysiological in vitro systems has opened new possibilities because such systems are more representative of in vivo 3D structures. These models enable the development of individualized approaches by using patient‐derived cells [28], potentially improving therapeutic prediction [29]. Improved and accurate models and screening tools would accelerate the translation of effective anti‐cancer therapeutics by early elimination of poor drug candidates and saving billions of dollars to bolster the efforts on the selected drug candidates to be launched on the market [23, 30, 31].

Among 3D microphysiological in vitro systems, 3D bioprinting (3DBP) has emerged as a powerful platform for constructing multicellular models of breast cancer. 3DBP enables replication of the complexity of TME by combining cancer cells with various supporting cell types, including fibroblasts, adipocytes, endothelial cells, and immune cells, thereby enhancing the model's physiological relevance.

Recent studies have utilized 3DBP to recreate the breast cancer multicellular TME by co‐culturing cancer cells with (1) one other cell type such as normal epithelial cells [32], fibroblasts [33, 34, 35], adipocytes [36], stem cells [37, 38, 39, 40], or lymphatic endothelial cells (LECs) [41]; (2) two other cell types, such as fibroblasts and endothelial cells [42, 43], stem cells and endothelial cells [44], or fibroblasts and mononuclear cells [45]; or (3) three other cell types (fibroblasts, stem cells and endothelial cells), adding more to TME complexity [46]. Furthermore, advanced 3DBP models of the breast cancer TME were developed using: (1) preformed spheroids comprising breast cancer cells alone [47, 48, 49], with fibroblasts [33, 50], with fibroblast and endothelial cells [51, 52], with MSCs and endothelial cells [53], or with normal epithelial, MSCs, and endothelial cells [53]; (2) a combination of preformed spheroids and individual cells (one [47] or two [42] cell types) was also explored; (3) preformed organoids [42]; and (4) 3DBP combined with microfluidics [54, 55, 56].

Recent work increasingly points to a shift in 3DBP breast cancer modeling from static, cell‐line‐based constructs toward patient‐derived or organoid‐informed systems that retain tumor heterogeneity, dynamically perfused models, and platforms coupling vascular and immune compartments to better reproduce transport, cell trafficking, and treatment responses [52, 57]. In parallel, automated multiwell bioprinting and high‐content readouts are enabling scalable, high‐throughput drug screening, while AI‐ and machine‐learning‐assisted image and data analyses are emerging for response quantification, although direct AI integration with bioprinted tumor models remains limited [58, 59, 60]. These human‐relevant platforms increasingly align with new approach methodologies (NAMs); however, their regulatory validation and clinical translation will require standardized materials and fabrication protocols, quantitative performance benchmarks, interlaboratory reproducibility, and fit‐for‐purpose validation against matched patient samples and clinical responses [61, 62, 63].

Despite progress, previous reviews have only partially addressed these developments. Existing reviews often cover bioengineering methods applied to breast tissue [64] and breast cancer models [65, 66, 67]; however, the 3DBP approach is only one part of these reviews. Reviews that address 3D‐printed cancer models [68] or regenerative applications of 3DBP [69] devote limited attention to 3DBP‐specific advances in breast cancer modeling [70, 71]. The two main reviews on 3DBP of breast cancer (published in 2018 and 2021) [72, 73] briefly discuss 3DBP and do not comprehensively analyze recent advancements in bioprinting strategies. The first review primarily covers TME and tissue engineering [72]. It covers 3DBP only in part, describing 3DBP techniques and materials used for breast cancer, especially in pharmaceutical studies. The second review is on “engineering breast cancer microenvironments and 3DBP” [73]. Both these reviews lack focus on breast cancer 3DBP and deep analysis of the evolution of 3DBP models of breast cancer in terms of mimicking TME and complexity. The field has advanced since the publication of these reviews in 2018 [73] and 2021 [72]. Other recent reviews are compared in Table S1.

To bridge this knowledge gap, the current review focuses specifically on 3DBP‐based breast cancer models, emphasizing: (1) the evolution of bioprinted models from simple one cell type‐cultures to complex, multiple cell type [32, 33, 34, 35, 36, 37, 38, 39, 40, 41, 42, 43, 44, 45, 46, 74, 75, 76, 77, 78, 79, 80, 81, 82, 83, 84, 85, 86, 87, 88, 89, 90, 91], vascularized systems; (2) the use of preformed spheroids [33, 47, 48, 49, 50, 51, 52, 53], preformed organoids [42], and microfluidic integration [54, 55, 56]; and (3) Applications in metastasis studies, vascular [56] and lymphatic modeling [41], and drug screening [92, 93, 94] (Figure 1).

**FIGURE 1:**
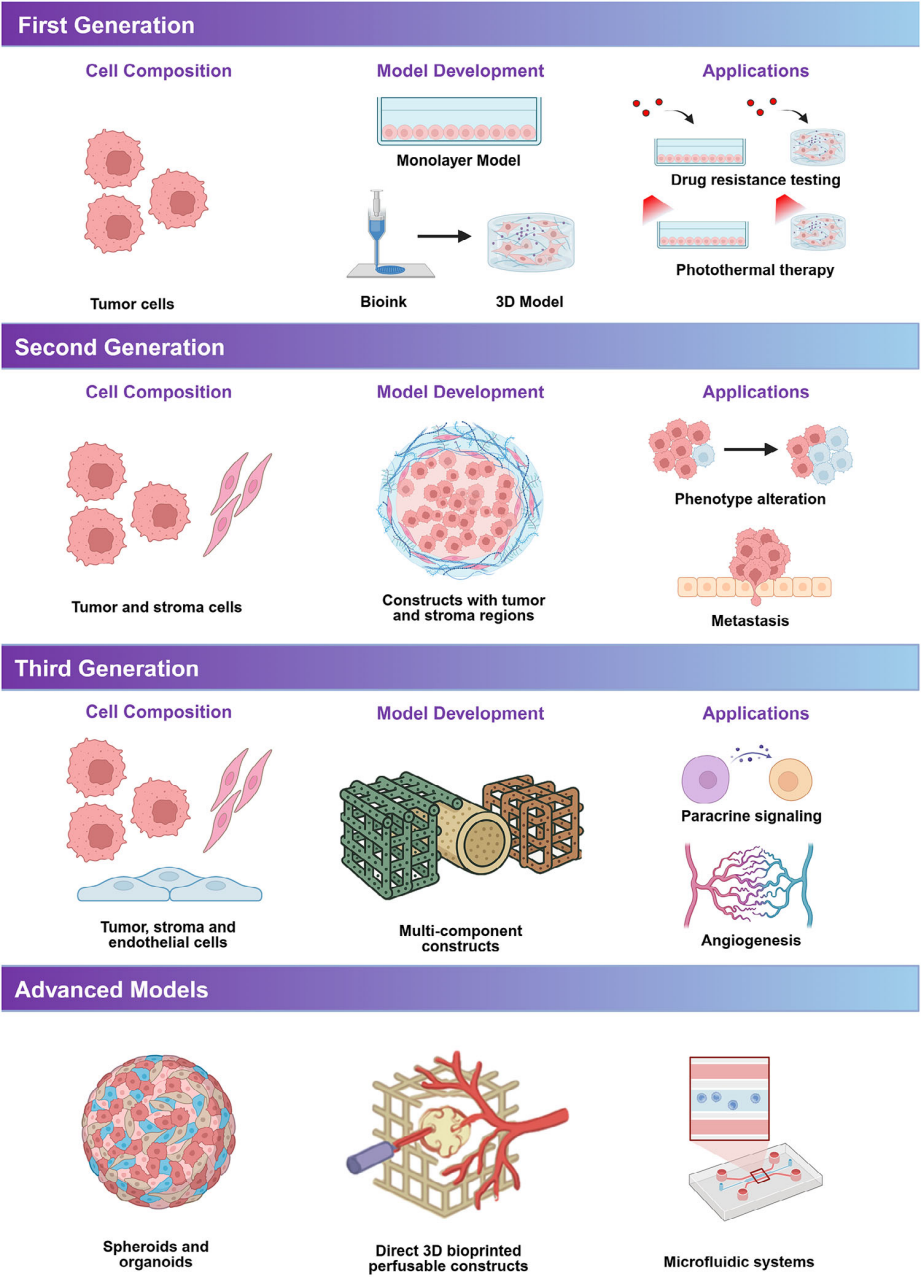
Overview of the 3DBP for breast cancer modeling. Created in BioRender, M. Nakipoglu, (2025), https://BioRender.com/bjimxhr.

To avoid repeating content well‐covered in prior reviews (e.g., various methods of 3DBP, materials used, tissue engineering, breast cancer anatomy, cancer, and TME), this review focuses on highlighting how unique advances in breast cancer‐specific 3DBP research have been achieved through the stepwise evolution of research in this field, organizing the discussion chronologically and thematically to guide future work. The scope of this review was limited to original studies in which 3DBP was used with breast cancer cells to create a tumor model. This scope encompasses directly bioprinted constructs as well as hybrid spheroid‐ and microfluidic‐based platforms when bioprinting contributes to model construction; their inclusion does not imply that spheroid formation or microfluidics are themselves bioprinting techniques. The generation‐based organization used in this study traces the broad development of the field from relatively simple cancer‐cell constructs toward models with increasing biological complexity, spatial organization, and functional integration of tumor‐microenvironment components. Because these advances have occurred partly in parallel, the categories are not mutually exclusive, and some chronological and conceptual overlap is expected.

## 2 Overview of Breast Cancer Modeling Using 3DBP

The objective of breast cancer modeling is to recapitulate tumor and TME as closely as possible [95, 96]. To achieve this, 3DBP was employed to enable control over cell positioning in the engineered tissue construct, enable the use of multiple cell types [32, 33, 35, 36, 37, 38, 39, 40, 42, 43, 44, 46], and create channels that mimic blood [93] and lymphatic [41] vessels, and incorporate immune cells [45]. In addition, it was used to develop metastasis models [92, 93, 94] and models that employ gradients of chemokines [53]. It provides an opportunity to study tumor cell behaviors under normal conditions. To be able to achieve this, various biomaterials were used to include tumor and other cell types and to mimic as much as possible the properties of tumor ECM in terms of mechanical properties, allowing spread and invasion, and metastasis [97].

### 2.1 Cells Used

The most common cancers of the breast are carcinomas, usually adenocarcinomas, which arise in the ducts or lobules of the mammary gland [98]. Among all breast cancer diagnoses, invasive ductal carcinoma accounts for 80% [99]. Although it accounts for only ∼15% of all breast cancers, triple‐negative breast cancer is an aggressive type of invasive breast cancer, which can be difficult to treat [98]. Triple‐negative breast cancer cells do not express estrogen or progesterone receptors (ER or PR) and express little or no HER2. Therefore, cell lines such as triple‐negative invasive ductal carcinoma cell lines (MDA‐MB‐231 [33, 34, 35, 37, 39, 41, 42, 43, 44, 46, 47, 50, 51, 54, 76, 81, 84, 86, 92, 93, 100, 101, 102], MDA‐MB‐468 [32, 79]) have been used in 3DBP models (Table 1).

**TABLE 1:** Cell lines used in the 3DBP of breast cancer models.

Nos.	Cell lines	Name derivation	Types	Years	Origin	Invasive?	Receptors	Useful in	3DBP studies
1	MCF‐7	Michigan Cancer Foundation	Ductal	1973	69‐Year‐old white woman [108]	Rapidly growing noninvasive [109, 110]	ER+, PR+, and HER2‐ [35, 111]	3DBP platform validation	[32, 35, 36, 38, 43, 46, 47, 54, 74, 75, 76, 78, 79, 80, 81, 82, 83]
2	MDA‐MB‐231	MD Anderson [112]	Ductal	1973	51‐Year‐old caucasian female [113]	Invasive	ER‐, PR‐, and HER2‐	Modelling late‐stage breast cancer [112]	[33, 34, 35, 37, 39, 41, 42, 43, 44, 46, 50, 51, 54, 76, 81, 84, 86, 92, 93, 100, 101, 102]
3	MDA‐MB‐468	MD Anderson	Ductal	1977	51‐Year‐old black female [114]	Invasive [109, 115, 116]	ER‐, PR‐, and HER2‐ [117]	Immuno‐oncology studies [114]	[32, 79]
4	ZR75.1	Zeida Rae [118]	Ductal	1978 [119]	63‐Year‐old white female [91, 120]	Invasive	ER+, PR+, and HER2 (low) [105]	Drug and metabolic profiling [91]	[91]
5	SK‐BR‐3	Sloan‐Kettering Breast Cancer	Ductal	1970	43‐Year‐old caucasian female [121]	Invasive [122]	ER‐, PR‐, and HER2+ (high expression)	Studying HER2‐based approaches	[45, 76]
6	BT‐474	Number of cells that grew from a particular dish [123]	Ductal	1978 [124]	60‐Year‐old white female [110]	Invasive	ER+, PR+, and HER2+ [125]	Forms compact, slowly growing multi‐layered colonies [125]	[55]
7	4T1	Clone 1 isolated from the fourth serial transplant	Murine	1978 [126]	BALB/c mouse [127]	Invasive	ER‐, PR‐, and HER2‐	Transplantable [126] Represents stage IV human breast cancer [128]	[88]
8	Primary				Human		2 Samples: ER+/PR+/HER2− 1 Sample: ER−/PR−/HER2+	Progress of normal cells to cancer	[89]
9	Primary				Mouse			3D culture and transplantation assays	[42]

70%–80% of breast cancers are ER positive [103]. ER plays an important role in the proliferation, survival and invasion of cancer cells, and it provides a target for therapy [104]. Hormone receptor‐positive (HR+)/HER2‐negative breast cancer emerged as a clinical challenge, for which the combination therapy comprising cyclin‐dependent kinases 4 and 6 (CDK4/6) inhibitors with endocrine therapy is becoming the standard option for both first‐ and second‐line treatments [105]. Cell lines such as ZR‐75‐1, which is ER+, PR+, and HER2‐low [105], can be useful although they have not been widely used in 3DBP models so far [91]. So far, breast cancer 3DBP studies most commonly involved the use of ductal carcinoma cell lines, namely, MCF‐7 [32, 36, 38, 43, 46, 47, 54, 74, 75, 76, 78, 79, 80, 81, 82, 83] and MDA‐MB‐231 [33, 34, 35, 37, 39, 41, 42, 43, 44, 46, 47, 50, 51, 54, 76, 81, 84, 86, 92, 93, 100, 101, 102]. Cell lines such as SK‐BR‐3 [45, 76], MDA‐MB‐468 [32, 79], and 4T1 [87] were also used. Some of these cell lines were used to produce spheroids that were employed in bioinks, including spheroids derived from MDA‐MB‐231 [47, 48, 51, 52], MCF‐7 [40, 47, 49], MCF‐10A [47, 48, 53], and MCF‐10AT [56].

Most of the studies used cell lines because of easier handling, infinite self‐replication, and a relatively high degree of homogeneity [106]. Primary cells, although more representative of the tumor, have limited time span, and they suffer from heterogeneity [106]. However, when using these models for developing personalized medicine, primary cells should be used [107].

### 2.2 Bioink Materials Used

Hydrogels used for the 3DBP of breast cancer models were mostly alginate [39, 87, 88, 91] or alginate‐containing materials. The latter group comprised mostly alginate and gelatin [33, 34, 36, 40, 46, 54, 77, 82, 85, 129]. This is because alginate is a biocompatible and inert material [39]. Gelatin provides elasticity and cell adhesion sites [34]. In addition, alginate/gelatin combined with other components was used, for example, with Matrigel [102], which contains basement membrane macromolecules [130], and it helps spheroid genesis [102]. Other hydrogels used for breast cancer 3DBP include gelatin‐based materials such as GelMA [75, 86]. Gelatin provides a good environment for cell attachment and proliferation. Decellularized ECM [79] and its derivatives [43, 79] were also used to better mimic TME (Table 2). Bioink composition strongly influences tumor behavior and drug response in 3DBP breast cancer models. Breast tumors generally exhibit stiffnesses above 4 kPa [131] and may reach approximately 42 kPa in highly fibrotic regions [132]; therefore, moderately stiff bioinks (∼3–20 kPa [89, 133]) can improve physiological relevance and shape fidelity, whereas excessive stiffness, often associated with higher viscosity, crosslink density, and smaller mesh size, may increase extrusion‐induced cell damage, restrict migration, and reduce drug diffusion [134, 135, 136]. Degradability must likewise be balanced to permit ECM remodeling, proliferation, invasion, and prolonged culture while preserving construct integrity: collagen, gelatin, GelMA, and dECM are susceptible to MMP‐mediated degradation [137], whereas alginate is not [138], and hyaluronic acid is primarily degraded by hyaluronidases [139]. Porosity and interconnectivity govern oxygen, nutrient, waste, and drug transport while also affecting cellular confinement and invasion [140]. Adhesive‐ligand presentation further regulates integrin signaling, survival, migration, stemness, and therapeutic sensitivity, with gelatin, collagen, Matrigel, and dECM providing more biologically active cues than alginate or PEG. However, the undefined composition and batch variability of Matrigel and dECM can alter stiffness, ligand density, degradation, and growth‐factor content, reducing reproducibility. Finally, crosslinking chemistry determines stiffness, mesh size, swelling, stability, ligand accessibility, and degradability; excessive crosslinking can impair remodeling and drug penetration [141, 142], whereas insufficient crosslinking compromises structural fidelity.

**TABLE 2:** Bioink biomaterials used in the 3DBP of breast cancer models.

Nos.	Bioink biomaterials	Advantages	Limitations	Refs.
A	Natural polymers			
i.	Alginate and alginate‐containing			
1.	Alginate	‐Biocompatible, biologically inert [39] and ionically crosslinkable at physiological temperature by divalent cations [143]. ‐Easy cell encapsulation due to slow gelation at mild temperatures [40, 144, 145].	‐Poor mechanical properties.	[39, 87, 88, 91]
2.	Alginate and gelatin	‐Enhanced thermal stability and stiffness compared to gelatin alone, while maintaining good mechanical integrity [146]. ‐The combination leverages complementary properties: gelatin (denatured collagen) provides elasticity and cell‐adhesion sites, whereas alginate is bioinert but ensures structural support [34]. ‐Improved cell‐binding ability compared to alginate alone, supporting cell viability, proliferation, and migration [146]. ‐Mechanical properties can be tuned by adjusting the gelatin/alginate ratio and divalent cation concentration [36, 40, 85]. ‐Post‐printing immersion in divalent cation solutions enables ionic crosslinking, forming a core–shell structure that facilitates cell migration [33]. ‐Water‐soluble natural polymer system with uniform extrusion, structural integrity, and high print fidelity due to synergistic interactions [82, 147].	‐When alginate concentration is low, samples have insufficient recovery (due to low gelatin %) and cohesive properties (due to low alginate %), which results in higher filament roughness [85]. ‐When alginate concentrations are decreasing in hydrogel compositions, the gels become soft [85].	[33, 34, 36, 40, 46, 54, 77, 82, 85, 129]
3.	Alginate, gelatin, and Matrigel	‐In addition to the advantages the combination of alginate and gelatin has, Matrigel contains basement membrane macromolecules such as laminin and collagen IV [130]. ‐Laminin is important in breast epithelial spheroid formation [47, 102]. ‐Matrigel component accelerates the gelation process and softens the bioink [102]. ‐Less expensive than Matrigel used alone.	‐Matrigel induces phenotypic changes to breast cancer epithelial cells [102]. ‐Matrigel batch‐to‐batch variability [47].	[47, 102]
4.	Alginate, gelatin, hydroxyethyl cellulose	‐Can be an alternative to commercial Matrigel, and it can be easily manufactured and standardized [80]. ‐Good printability and biocompatibility; key protein expressions of tumor cells with this bioink material are basically the same as those in Matrigel; printable at room temperature. ‐Cost‐effective and easy to obtain from nature [80].	‐It cannot perfectly simulate the ECM compared to Matrigel [80].	[47, 80, 102]
5.	Alginate and fibrin	‐Modular crosslinking via thrombin and divalent cations. ‐The combination offers the advantages of both materials, including enhanced cell–material interactions through fibrin, tunable degradation properties, and a TME mimicking environment.		[55]
6.	Alginate, peptide modified alginate, collagen and fibrinogen	‐Different crosslinking mechanisms (ionic, pH, and enzymatic) can be employed [44]. ‐Useful for fabricating heterogeneous constructs: fibrinogen is used to print the vascular layers, alginate for growth‐factor release, and alginate‐RGD/collagen for the fibroblast layer [44].	‐Complex crosslinking method limits the bioprinting process [44].	[44]
7.	Alginate and collagen	‐The combination offers improved biocompatibility, tunable mechanical properties, and enhanced biomimicry.		[47]
8.	Alginate, collagen, and hyaluronic acid (HA)	‐Gelatin and HA for enhancing printability. Alginate for providing a non‐adherent surface that induces cell spheroid formation [148]. ‐Alginate has excellent mechanical strength, rapid crosslinking, and no cell‐binding sites [148].		[47, 76]
9.	Alginate and boronic acid‐functionalized laminarin	‐Produced using double crosslinking, which allows dynamic interactions within the bioink network. This results in: (1) prevention of cellular sedimentation. (2) High cell survival due to minimal bioink shear stress during bioprinting [84]. ‐Long term construct stability after extrusion, endowed by divalent cation crosslinking [84]. ‐Laminarin is a biodegradable and cytocompatible polymer that exhibits immunomodulating and antioxidant activities [84, 149]. ‐Boronic acid enables the formation of reversible boronate ester complexes between the boronic acid groups on laminarin and diol groups on alginate [84, 148, 150].	‐Laminarin has anticancer activity, which may interfere with cancer models and their response to therapy.	[84]
10.	Alginate, GelMA and decellularized porcine breast tissue ECM	‐Tissue‐derived hydrogels have bioactive properties [38]. ‐Decellularized breast and adipose tissues can mimic the ECM [38]. ‐GelMA provides better printability of the ECM; alginate provides stiffness and integrity [38, 84, 149, 150].		[38, 84]
ii.	Gelatin or gelatin containing			
1.	Gelatin and Matrigel	‐Combines gelatin and Matrigel advantages listed above.		[151]
2.	GelMA	‐Combines gelatin advantages that include cell‐attachment motifs and photo‐crosslinkability. ‐Widely used for 3DBP with proved printability and biocompatibility.	‐UV‐induced crosslinking and the associated photoinitiators can exert cytotoxic effects, posing risks to primary cells [89]. ‐Mammalian‐based gelatins have limited use for drop‐on‐demand bioprinting due to high viscosity and shear‐thinning at room temperature [89].	[75, 86]
3.	GelMA and agarose as a sacrificial material	‐In addition to the advantages of using GelMA listed above, a printed agarose microfiber can be gently removed from the GelMA construct as a sacrificial component using physical force. This allows the formation of hollow microchannels of a desired size and morphology in the hydrogel [75, 152].		[41, 75]
4.	GelMA and methacrylated hyaluronic acid (MeHA)	Enhanced hydrophilicity, printability and structural integrity, cell behavior and function, and TME mimicking environment.		[37]
5.	GelMA, thiolated‐gelatin (GelSH) and adhesion peptide‐functionalized PEG	‐PEG can be a malleable alternative to Matrigel [89]. ‐Tuning GelMA crosslinking density enables the formation of physiologically relevant breast, normal or pathological, environments. ‐In contrast to mammalian gelatin, fish gelatin shows Newtonian‐like flow behavior and has lower viscosity at room temperature. Its thiolated form can undergo Michael‐type click crosslinking to form hydrogels near‐instantaneously [89]. ‐The three hydrogels provide stiffnesses that support the formation and growth of organoids for more than 3 weeks.	‐PEG lacks protein adsorption [89]. ‐Problems with mammalian‐derived gelatin (e.g., high viscosity). ‐Reduced drug potency in thiolated gelatin, which needs to be investigated further [89].	[89]
6.	GelMA/Polyethylene glycol diacrylate (PEGDA) and GelMA/PEGDA/HAp	‐GelMA has been widely used as a bioink for 3DBP. ‐The combined GelMA–PEGDA hydrogel provides tunable stiffness and has been investigated as a bone matrix for osteoblast 3D culture [153]. ‐Contains the mineral HAp to mimic bone microenvironment.		[93]
7.	GelMA with nHAp	‐nHAp component improves osteoblast and MSC proliferation in the constructs [92]. ‐GelMA and nHAp are used to mimic native bone tissue matrix. ‐Useful for developing models of metastasis to the bone [92].		[92]
iii.	Collagen and collagen‐containing			
1.	Collagen	‐Has motifs that help cell attachment and function.		[32]
2.	Collagen	‐Semi‐crosslinked nature (achieved by incubation) of collagen makes it suitable for embedded bioprinting. ‐TME typically comprises of ECM proteins such as collagen and fibrin. Crosslinking of collagen is slower than that of fibrin at room temperature, making it favorable for bioprinting [50].	‐Very low viscosity at low concentrations, which makes 3DBP challenging [42].	[50]
3.	Collagen and fibrin	‐TME typically comprises ECM proteins such as collagen and fibrin [50].	Crosslinking of fibrin is faster than that of collagen at room temperature, which can be challenging for 3DBP [50].	[50, 52]
4.	Collagen/Carboxylated agarose (CA) hydrogel‐based bioink	‐Collagen can be used to mimic the biological barrier (basement membrane) that tumors have to remodel and traverse. ‐CA bioink is biologically inert and thus can support cell growth but cannot be remodeled by the cells, ensuring that tumor spheroids remain intact over the study period and that their collective behavior can be elucidated.		[52, 53]
5.	Collagen–HA–[poly(*N*‐isopropylacrylamide)] (pNIPAM). Silk fibroin (for support)	‐The use of a thermoresponsive HA‐based polymer optimizes the bioink composition to make it suitable for maintaining different cell phenotypes. ‐Silk fibroin is a biocompatible material that can be used for producing a physically crosslinked hydrogel.		[42, 53]
iv.	Others			
1.	Elastin‐like protein	‐Tunable matrix properties. ‐Cell‐laden ELP hydrogels are stable for at least 2 weeks [56]. ‐Proteolytically degradable by proteases [56]. ‐Protein‐engineered ELP‐RGD hydrogels are printable and have motifs to help cell adhesion and proliferation [154].		[56]
2.	Fibrin	‐TME typically comprised of ECM proteins such as collagen and fibrin [50]. ‐Fibrin has an important role in angiogenesis [155].	‐Crosslinking of fibrin is faster than that of collagen at room temperature, which can be challenging for 3DBP [50].	[51]
3.	Thiol‐modified HA, allyl‐functionalized poly(glycidol)	‐Allyl‐functionalized poly(glycidol) can be used for UV‐photocrosslinking [156]. ‐HA‐based hydrogels are suitable for printing cells [157] and spheroids [101].		[101]
v.	Tissue or ECM derived			
1.	Decellularized whole human breast tissue	‐Decellularized whole human breast tissue can form a self‐gelling hydrogel according to the developed protocol [79]. ‐These ECM scaffolds contain growth factor patterns unique to the mammary gland, allowing common cancer cell lines to proliferate [79].		[79]
2.	Gel from ECM from decellularized human and rat mammary tissue	‐Cancer cells grow into tumoroids within mammary‐derived ECM extracts due to the presence of growth factor composition in tissue‐specific ECM [79].		[79]
3.	Gel derived from porcine skin and decellularized human adipose tissue	‐Porcine breast skin tissue is similar to human skin tissue. ‐Decellularized human adipose tissue provides an accurate platform to model early‐stage breast cancer.	‐When used for investigating plasmonic photothermal therapy, the bioprinted 3D construct showed a smaller temperature increase as compared to the human tissue simulation, suggesting that more factors must be considered for fabricating a better skin construct.	[79, 83]
4.	Mix of porcine breast decellularized ECM (dECM) and HAMA	‐dECMs more closely replicate the native ECM microenvironment while lacking immunogenic nucleic acids and tumor‐promoting components commonly present in tumor‐derived matrices such as Matrigel [158, 159]. ‐They partially recapitulate breast tumor complexity, including tissue architecture, spatial organization of tumor and stromal cells, and relevant ECM signaling within the TME [43]. ‐HA is a biocompatible biomaterial, also known to be overexpressed in breast tumors [160]. ‐HAMA can serve as a rheological modifier to improve the viscosity of the ink. The incorporation of HAMA into the dECM bioink increases ink viscosity and improves its printability [43].		[43]
5.	Matrigel (ECM hydrogel derived from a mouse tumor) [130]	Allows the organization of breast epithelial cells into polarized spheroids that with an acinar structure [48, 158, 159, 160].	‐Limited capacity for precise biophysical tuning to model diverse cancer microenvironments and prone to batch‐to‐batch variability [89]. ‐Poorly defined composition (mixture of laminins, collagen IV, entactin), resulting in limited standardization, batch variability, and reduced reproducibility in cell studies [161].	[48]
B	Synthetic polymers			
1.	PEG	‐PEG can be a malleable alternative to Matrigel [89] ‐PEG bioink formulations can be developed to resemble mechanical properties of tissues [35].	‐PEG lacks protein absorption [89].	[35]
2.	PEG dimethacrylate (PEG DMA; to mold concave wells), sacrificial gelatin	‐PEG‐based hydrogels are biocompatible and photocrosslinkable. ‐PEG‐based hydrogels are non‐adhesive, making them suitable for spheroid formation. Non‐adhesiveness to the biomaterial encourages cell‐to‐cell interaction and aggregation, which is critical for spheroid development [162, 163]. ‐Gelatin is a suitable sacrificial biomaterial, as it is biocompatible, injectable and has reversible gelling ability under mild temperature conditions [74].		[74]

### 2.3 3DBP Techniques Used

Although various techniques have been used for 3DBP, most 3DBP studies of breast cancer models employed extrusion‐based 3DBP [32, 33, 34, 36, 38, 40, 43, 44, 45, 46, 47, 48, 53, 54, 55, 56, 73, 76, 79, 80, 82, 83, 84, 88, 93, 100, 101, 102, 164], because of its ease of use and cost‐effectiveness [42] and its capacity to use high‐cell‐density and highly viscous bioinks [165, 166]. In addition, few studies employed inkjet [35, 78, 81, 87] to offer faster printing and lower cost as compared to extrusion [165, 166]. Less often sacrificial 3DBP was used for breast cancer model fabrication. One study employed laser‐based 3DBP [39], which offers high precision and cell viability; however, it is slower than inkjet [166]. Stereolithography (SLA) was also used in another study [92]. SLA is characterized by high resolution [167], but its use is limited by cost and maintenance constraints [168]. The use of sacrificial 3DBP [41, 75] enables us to produce constructs with channels representing blood vessels. In more recent studies, where spheroids were used in bioinks, aspiration assisted 3DBP was used [50, 51, 52]. In addition, the recent development of the embedding technique (extrusion of viscoelastic inks through the nozzles inserted (embedded) into an elastomeric reservoir) [166, 169] allows the construction of larger, more complex constructs by printing very‐low‐viscosity bioinks inside a suspension bath, which prevents the printed construct from spreading; this technique has been used for 3DBP of a breast cancer construct [42]. Although it is a promising technique, its use is limited by the incompatibility of current bioprinters with embedded bioprinting, limited availability of materials for support bath preparation, similar crosslinking mechanisms for support bath and functional bioinks, and sterilization of support baths [170]. Readers interested in more details on 3DBP techniques for cancer models, including breast cancer, are referred to excellent reviews on the subject [72, 170, 171].

## 3 Evolution of 3DBP Breast Cancer Models That Have Increasing Levels of Development and Complexity

The objective of the first‐generation studies was to develop 3D structures that can better mimic in vivo tumors as compared to conventionally used 2D cultures. In the second generation, the objective was to advance these models further by enhancing TME biomimicry. In the third generation, multiple cell types were used to enable better TME mimicry and include cross talk between different cell types. Advanced 3DBP models focus more on preformed tumor spheroids/organoids and complex tumor constructs to study metastasis with or without the integration of microfluidic devices. Last, integration of printed constructs with vascularized tissues ex vivo enhances these models to a different level where in vitro and in vivo approaches are combined (Table 3).

**TABLE 3:** Summary of 3DBP breast cancer models that evolved through generations of development.

Model class	Role of 3DBP	Capabilities	Limitations	Refs.
First generation (cancer‐cell‐only)	Standardize cell placement, geometry, matrix, and assay scale.	Isolate tumor‐intrinsic, matrix, and printing effects; enable scalable testing.	Dynamic transport; controlled oxygenation; standardized exposures/readouts.	[28, 39, 40, 74, 75, 76, 77, 78, 79, 80, 81, 82, 83, 85, 86, 88, 89, 90, 91, 102, 172, 173]
Second generation (cancer + one supporting population)	Set cell proximity, compartments, interfaces, channels, or sensors.	Resolve tumor–stroma, –vascular, or –bone crosstalk.	Patient‐matched pairs; perfusion; independent material/geometry control.	[32, 33, 36, 37, 38, 101, 133, 141, 174, 175, 176, 177, 178, 179, 180, 181]
Third generation (multicomponent TME)	Organize interacting compartments, interfaces, vessels, and immune access.	Integrate immune–vascular–lymphatic–stromal regulation.	Perfusable vessels; barrier/drainage assays; autologous cells; cell‐resolved endpoints.	[41, 42, 43, 44, 45, 46, 182, 183]
Metastasis models	Connect tumor, vascular, and secondary‐site compartments.	Reconstruct sequential dissemination and tissue‐specific colonization.	Complete cascades under flow; lung/liver/brain niches; resident immune cells; long‐term validation.	[92, 93, 94, 184]
Preformed spheroid models	Control spheroid placement, spacing, depth, and coupling to cells/channels.	Assemble preorganized tumor units for mechanistic and therapeutic studies.	Patient‐derived/Heterotypic spheroids; controlled maturation; dynamic transport; cell‐resolved analysis.	[47, 48, 49, 50, 51, 52, 53, 185, 186]
Preformed organoid models	Position organoids reproducibly and parallelize matrix/condition testing.	Preserve patient heterogeneity for personalized modeling and response assessment.	Breast patient‐derived organoids; matched TME; perfusion; long‐term culture.	[42]
3DBP–microfluidic models	Define starting geometry/composition; chips control gradients, flow, and transport.	Enable dynamic, multivariable modeling with controlled exposure and sampling.	Physiological flow/oxygen; permeability/adsorption standards; immune–lymphatic integration.	[54, 55, 187, 188]
Ex vivo tissue‐integrated models	Control cell pattern and tumor–tissue distance while retaining native tissue.	Leverage native ECM, resident cells, and microvessels with limited reconstruction.	Tissue standardization.	[87, 189, 190]

### 3.1 First Generation: Cancer‐Cell‐Only 3DBP Models

First‐generation models contain breast cancer cells without deliberately incorporated stromal, vascular, lymphatic, or immune cells. They have been developed predominantly from established human or murine breast cancer cell lines [74, 75, 76, 77, 78, 79, 80, 81, 82, 83, 85, 86, 87, 88, 90, 91] and, less frequently, patient‐derived tumor cells [89]. These platforms use bioprinting to control cell aggregation, tumor geometry, matrix conditions, or assay reproducibility, enabling tumor‐intrinsic effects of 3D organization on growth, invasion, and therapeutic response to be examined. However, their lack of heterotypic tumor‐microenvironment interactions limits their biological and translational relevance [74, 75, 76, 77, 78, 79, 80, 81, 82, 83].

#### 3.1.1 Early Models

Early cancer‐cell‐only models used bioprinting primarily to standardize the geometry within which tumor cells subsequently organized. One such model used MCF‐7‐laden gelatin droplets as sacrificial templates within photocrosslinked PEGDM arrays, combining microwell fabrication with in situ cell seeding (Figure 2A) [74]. Altering droplet volume and initial cell density controlled well depth and aggregate size. The platform maintained high cell viability and improved well‐to‐well consistency, demonstrating reproducible control over spheroid position and dimensions.

**FIGURE 2:**
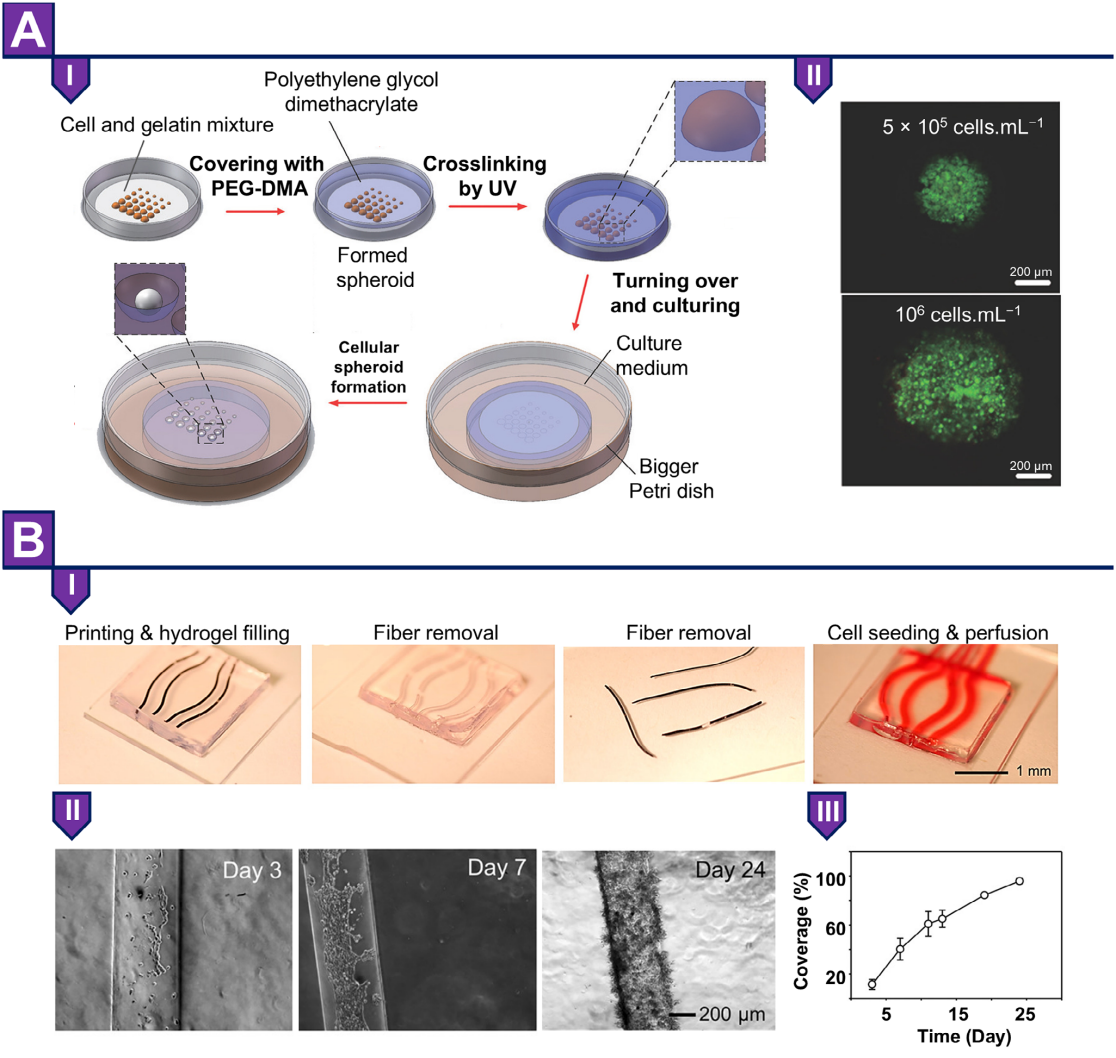
Early models of 3DBP breast cancer. (A) Concave‐well fabrication and in situ cell seeding for cellular spheroid formation. (I) Stepwise process creates cellular spheroids by printing cell‐laden gelatin onto a PTFE‐covered petri dish, forming a hydrogel array at 4°C, encapsulating it in PEG‐DMA via UV crosslinking, and then releasing the cells by liquefying the gelatin at 37°C. (II) Influence of initial in situ cell‐seeding density on the formation of cellular spheroids. (B) Mammary ductal carcinoma model. (I) Bioprinted agarose microfibers in the cast GelMA hydrogel, microchannels formed after removal of the agarose microfibers, the removed agarose microfibers, and perfusion of the microchannels. (II) Proliferation of MCF‐7 cells in 24 days. (III) Percentage coverage of the cells. Figure A was reproduced from ref. [74] (an open‐access article under the CC BY license, http://creativecommons.org/licenses/by/4.0). Figure B reproduced with permission.[75] 2019, Wiley.

Another work extended this architectural control to mammary duct‐like geometry by extrusion‐printing sacrificial agarose fibers within GelMA, removing the fibers, and manually seeding MCF‐7 cells into the resulting channels (Figure 2B) [75]. Printing permitted control of channel diameter, tortuosity, and branching while producing perfusable structures. MCF‐7 cells seeded within these channels underwent epithelial‐to‐mesenchymal transition (EMT), evidenced by the downregulation of E‐cadherin and moderate upregulation of N‐cadherin at invasion fronts. These cells invaded the surrounding matrix by Day 19, mimicking invasive ductal carcinoma and enabling real time analysis of invasion and ECM remodeling. Nevertheless, manual seeding produced heterogeneous cell distribution and GelMA provided limited mammary‐specific matrix cues.

#### 3.1.2 Investigating Bioink Material

Mammary dECM provides tissue‐specific biochemical complexity for 3DBP breast cancer models: MCF‐7 and MDA‐MB‐468 cells in hydrogels derived from decellularized human mammary tissue demonstrated expression profiles distinct from those in Geltrex [79]. However, the clearest ductal branching and associated changes in Ki‐67 and CK5 occurred in nonmalignant MCF‐12A cells (Figure 3A,I). This demonstrated mammary‐specific matrix bioactivity more clearly than a cancer‐specific advantage, while the compositional complexity and source dependence of dECM limit experimental control and reproducibility.

Bioink composition critically influences encapsulated‐cell behavior and spheroid formation. Independent variation of alginate (1%–5%) and gelatin (5%–9%) concentrations enables the production of crosslinked hydrogels with Young's moduli in the 5–23 kPa range [85]. Here, modulus is governed predominantly by alginate concentration, whereas increasing gelatin supplies more potential adhesion sites without substantially changing stiffness. For MDA‐MB‐231 cells, the A1G7 formulation (1% alginate and 7% gelatin; approximately 8 kPa) produced medium and large spheroids by Day 14, whereas the stiffer A3G7 formulation (3% alginate and 7% gelatin; approximately 13 kPa) produced large spheroids only by Day 28 (Figure 3A, II). Although these differences cannot be attributed exclusively to stiffness, they highlight the interrelationship among polymer composition, matrix mechanics, and spheroid formation. In a similar work, matrix‐dependent mechanical adaptation and drug response were further examined in DLP‐printed MDA‐MB‐231 constructs formed from a soft GelMA bioink (compressive modulus, ∼7 kPa) or a stiff GelMA–PEGDM bioink (∼44 kPa) [172]. By Day 14, spindle‐like spreading and a fourfold increase in metabolic activity were observed in the soft matrix, whereas predominantly rounded cells and minimal metabolic change were retained in the stiff matrix. Cells recovered from the printed scaffolds had higher elastic moduli than 2D‐cultured cells, and 48‐h exposure to 1 µM doxorubicin produced 45% cell death in the soft constructs but only 7% in the stiff constructs.

**FIGURE 3:**
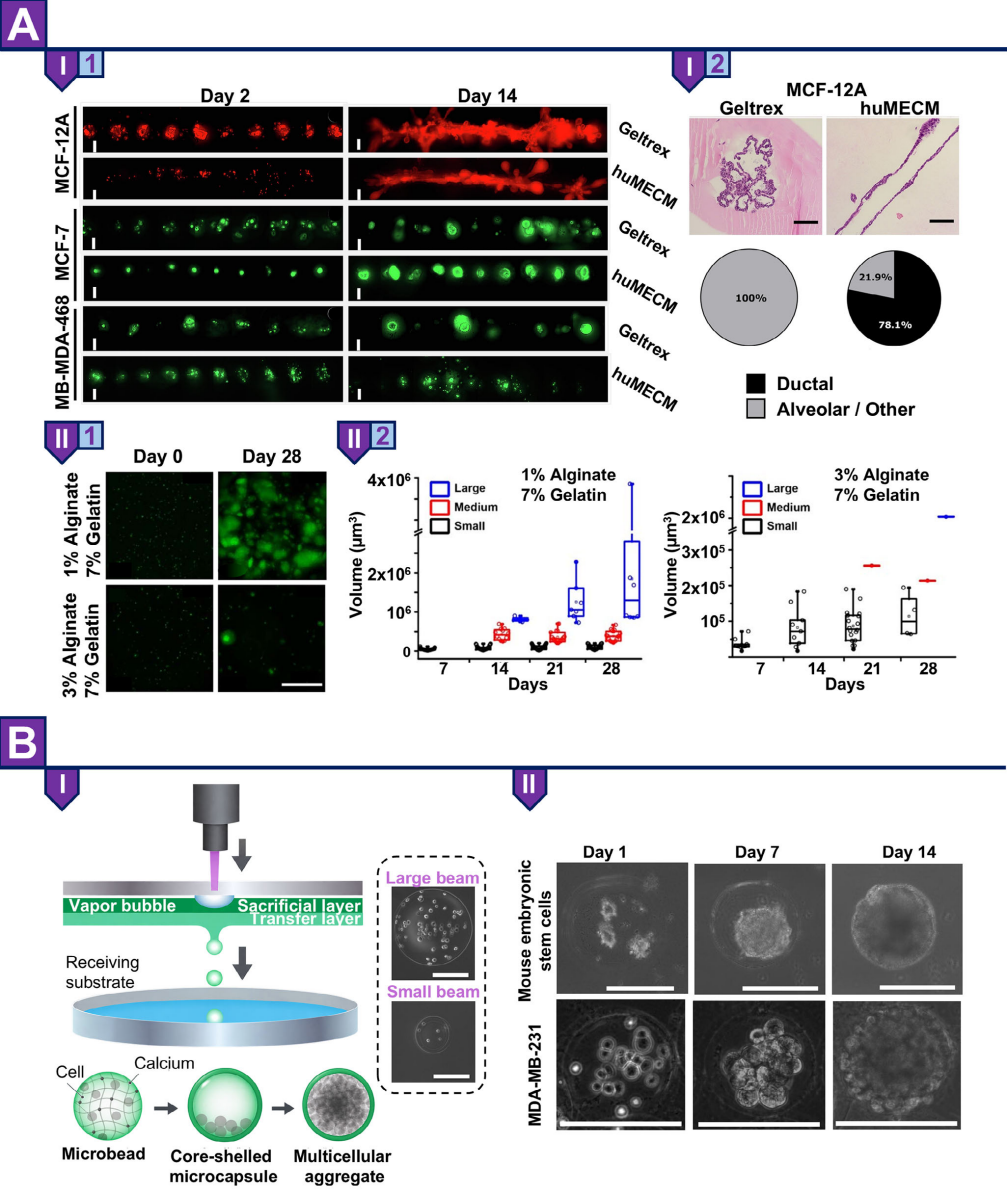
Investigating bioink material and bioprinting for 3DBP breast cancer models first generation. (A) Effect of bioink content on spheroid/organoid growth and morphology. (I) Ductal structure formation (1) Morphological variations across different breast cancer cell types (MCF‐12A, MCF‐7, and MDA‐MB‐468) in human mammary derived extracellular matrix hydrogels (huMECM). Larger organoids were observed on Geltrex Scale bar 200 µm. (2) Effect of substrate on ductal structure formation. While huMECM hydrogels enabled the formation of ductal structures, Geltrex did not yield ductal features. Scale bar 100 µm. (II) Effect of matrix stiffness on spheroid size. (1) Multicellular tumor spheroids in hydrogels with different compositions. (2) Spheroid volumes during 28 days of culture. Increased alginate content yielded smaller spheroids due to increased matrix stiffness. Scale bar 500 µm. (B) Laser‐based 3DBP. (I) 3DBP was enabled by a quartz disk spin‐coated with alginate–gelatin as a sacrificial layer and a pipette‐added cell–alginate suspension as a transfer layer. Petri dish at the bottom was spincoated with gelatin‐CaCl_2_ and acted as the receiving substrate. Laser beams hit the sacrificial layer to generate a vapor bubble, which ejects a sphere from the transfer layer to the receiving substrate for subsequent Ca^2+^ crosslinking. The inset image shows the precise control of beam diameter on sphere size (scale bar 200 µm). (II) Development of core–shelled structures generated with a 200‐µm beam. The cells grew and merged into morphologically distinct aggregates. Scale bar 200 µm. Figure A, (I) reproduced with permission.[79]. 2019, Elsevier. Figure A, (II) reproduced with permission.[85] 2019, IOP Publishing. Figure B reproduced with permission.[39] 2019, Elsevier.

Composite formulation can also improve printability and construct handling. The addition of 1% hydroxyethyl cellulose to 1% alginate–5% gelatin increased the compressive modulus from 7.9 to 13.0 kPa and enabled printing near room temperature with approximately 98% initial MCF‐7 viability [80]. This model demonstrated comparable performance to commercial basement membrane extracts in terms of clinically relevant biomarkers including Ki‐67, MUC1, and PARP1.

To assess whether serial passaging could support prolonged spheroid culture, MDA‐MB‐231 cells were bioprinted in an alginate–gelatin bioink [102]. After each 21‐day culture cycle, the alginate network was de‐crosslinked through citrate‐mediated calcium chelation, and the released spheroids were dissociated before the cells were re‐encapsulated and reprinted for at least three cycles. Post‐harvest viability was 93%, and the reprinted cells maintained their division rate and spheroid‐forming capacity. Adding 5% Matrigel accelerated gelation but reduced the storage modulus by ∼20%. It also altered spheroid growth, with some MDA‐MB‐231 multicellular structures reaching projected areas of approximately 1.3 mm^2^—up to 1000‐fold greater than the Day‐21 geometric mean of approximately 1300 µm^2^.

Dynamic double‐network crosslinking was used to balance extrusion behavior with post‐print stability [84]. A bioink containing 5% phenylboronic‐acid‐functionalized laminarin and 2.5% alginate was initially formed through reversible boronate ester bonds and subsequently reinforced by calcium‐mediated alginate crosslinking. Shear‐thinning behavior and a compressive modulus of 11 kPa were obtained, while MDA‐MB‐231 cells maintained over 90% viability for 14 days and were distributed throughout constructs up to 800 µm thick.

Stronger mechanistic comparisons were enabled by more controlled matrices. In patient‐derived breast cancer organoids, greater growth was supported by PEG functionalized with RGD, GFOGER, and DYIGSR than by formulations containing fewer adhesion peptides [89]. Matrix viscoelasticity can be more directly isolated using stiffness‐matched static and stress‐relaxing hydrogels [90]. TGF‐β‐induced invasion of premalignant MCF10AT cells was observed only in the dynamic matrix, while greater invasion was supported in spatially printed RGD regions than in nonbinding RDG regions.

#### 3.1.3 Investigating 3DBP Process

Beyond bioink composition, the printing process determines deposition accuracy and can itself alter cell viability and molecular state. Thermal, mechanical, electrical, photochemical, and post‐processing exposures must therefore be distinguished from phenotypes arising from 3D organization.

Precise droplet deposition was achieved within a restricted voltage window using electrohydrodynamic printing. MDA‐MB‐231‐laden 5% GelMA droplets, with minimum volumes of approximately 0.1 µL, were deposited onto a transparent conductive membrane to produce a 96‐well‐compatible 3D tumor array chip [86]. As voltage was increased, droplet formation transitioned from controlled microdripping to Taylor jetting, unstable multi‐jetting, and electrical breakdown. Stable deposition was obtained using a 32G nozzle at 2.7 kV and 10 µL min^−1^, and chamber‐to‐chamber cell‐number fluctuation was reduced from −99.5% to +136% without applied voltage to −4.6% to +3.1% at 2.7 kV. Viability above 90% was maintained for 7 days, with cell spreading observed from Day 1 and larger tumor‐like aggregates formed by Day 7. Reproducible fabrication of miniaturized tumor arrays was therefore achieved. However, subtler effects of the electric field and 405‐nm photocrosslinking were not excluded through molecular analyses or matched nonprinted 3D controls.

Control over aggregate size and spatial position was also achieved using laser direct‐write printing. By varying the laser‐beam diameter, MDA‐MB‐231‐laden alginate beads measuring 202 or 410 µm were produced and subsequently converted into liquid‐core, chitosan‐shelled capsules (Figure 3B) [39]. Cellular self‐assembly occurred within the capsules over 14 days, and final aggregate size was determined by capsule dimensions. However, cumulative processing stress was evident: viability decreased from 98% on the printing ribbon to 84% after printing and 76% after capsule processing. Greater heterogeneity in transferrin uptake was observed in larger aggregates; however, this represented a ligand‐transport proxy rather than direct evidence of drug penetration.

Collectively, modality‐specific trade‐offs were identified rather than a universally superior printing method. Substantial cellular perturbation was induced by thermal inkjet printing; array uniformity was improved by electrohydrodynamic printing, although voltage and photocrosslinking required careful control; and aggregate dimensions were regulated by laser direct‐write printing at the cost of reduced viability during multistep processing. Process validation should therefore include time‐resolved viability, apoptosis, and molecular analyses together with matched nonprinted 3D controls of equivalent cell density, matrix composition, and geometry.

#### 3.1.4 Application Studies

Cancer‐cell‐only models have been used to determine how 3D architecture, matrix composition, and assay configuration modify drug response.

Drug potency is affected by the interaction between culture format and compound structure. For 13 flavone phosphoramidates bearing different amino acid substituents, the 3D‐to‐2D IC_50_ ratios in bioprinted MCF‐7 constructs composed of hydroxyethyl cellulose, alginate, and gelatin ranged from 0.8 to 13.5 [80]. The alanine‐substituted derivative exhibited the greatest increase in IC_50_, whereas the phenylalanine‐substituted derivative showed little change, demonstrating that the effect of culture dimensionality was strongly structure‐dependent. This difference might be attributed to compound solubility, with less soluble derivatives potentially being less affected by the transition from 2D to 3D culture. In the same study, the doxorubicin IC_50_ was approximately threefold higher in 3D constructs than in monolayer cultures. Similar reductions in doxorubicin, epirubicin and paclitaxel sensitivity were observed in MDA‐MB‐231 spheroids relative to monolayer cultures [86, 102].

Morphology‐dependent responses were examined using printed ductal and solid tumor structures generated from MCF‐7, SK‐BR‐3 and MDA‐MB‐231 breast cancer cell lines [76]. Compared with ductal structures, solid aggregates exhibited more than eightfold greater hypoxia, two‐ to fourfold higher mesenchymal‐marker expression, and more than 15‐fold greater invasion. Greater resistance to doxorubicin but greater sensitivity to the hypoxia‐activated drug tirapazamine were also observed. While structural morphology was significant in these outcomes, the morphological difference was accompanied by differences in cell density and bioink formulation, which might also have contributed to the observed effects.

Assay standardization was addressed by integrating controlled fabrication, drug exposure, and image analysis into a single bioprinted miniwell workflow [40]. Bulk or CD44‐selected, resistance‐enriched MCF‐7 cells were printed at a fixed density in gelatin–alginate constructs, cultured for 5 days, exposed to a range of camptothecin or paclitaxel concentrations for 48 h, and analyzed in situ using the cell‐death marker 7‐AAD. Following treatment, resistance‐enriched spheroids exhibited significantly lower 7‐AAD fluorescence than bulk‐cell spheroids. Their dose–response data also showed substantially smaller standard deviations, which facilitates statistically significant differentiation among treatment groups.

More translational comparisons were attempted using xenograft models [91, 173]. Digital light processing bioprinting was used to fabricate GelMA constructs containing MDA‐MB‐231 or MCF‐7 cells recovered from corresponding mouse xenografts, with or without acellular blood vessel‐like channels [173]. Channel incorporation reduced the reported doxorubicin IC_50_ by 4.6‐fold in MDA‐MB‐231 constructs but only 1.2‐fold in MCF‐7 constructs; the corresponding reductions for capecitabine were 3.9‐ and 2.4‐fold, respectively. However, the dosewise viability data indicated a more distinctly drug‐dependent effect. Channel presence produced relatively little or inconsistent change in the viability of doxorubicin‐treated constructs, particularly for MCF‐7 cells, whereas capecitabine‐treated channel‐containing constructs exhibited approximately half the viability of channel‐free constructs across much of the tested concentration range. Engineered vascular‐like structures may therefore substantially alter the apparent response to some drugs while exerting limited effects on others, rather than uniformly increasing drug sensitivity.

Beyond conventional therapeutic screening, carcinogen‐induced stem‐like phenotypes were quantified using bioprinted miniwells (Figure 4A) [82]. After 6 days of culture, exposure to 0.2–10 µM benzo[a]pyrene for 24 h increased the CD44^+^/CD24^−^/low/ALDH1^+^ population from approximately 1.8% to 11.3% in 3D spheroids, compared with an increase from 1.1% to only 1.8% in monolayer cultures. Following exposure to 10 µM benzo[a]pyrene, the constructs were treated with 5 or 10 mM metformin, which reduced the marker‐positive population in a dose‐dependent manner. These results demonstrate the responsiveness of the assay to carcinogen‐induced cancer stem cell‐like enrichment and its pharmacological modulation. However, matched 2D and nonprinted 3D treatment controls were lacking, and further investigation is needed to explain the greater CSC‐like enrichment observed in 3D spheroid constructs than in 2D monolayer cultures.

**FIGURE 4:**
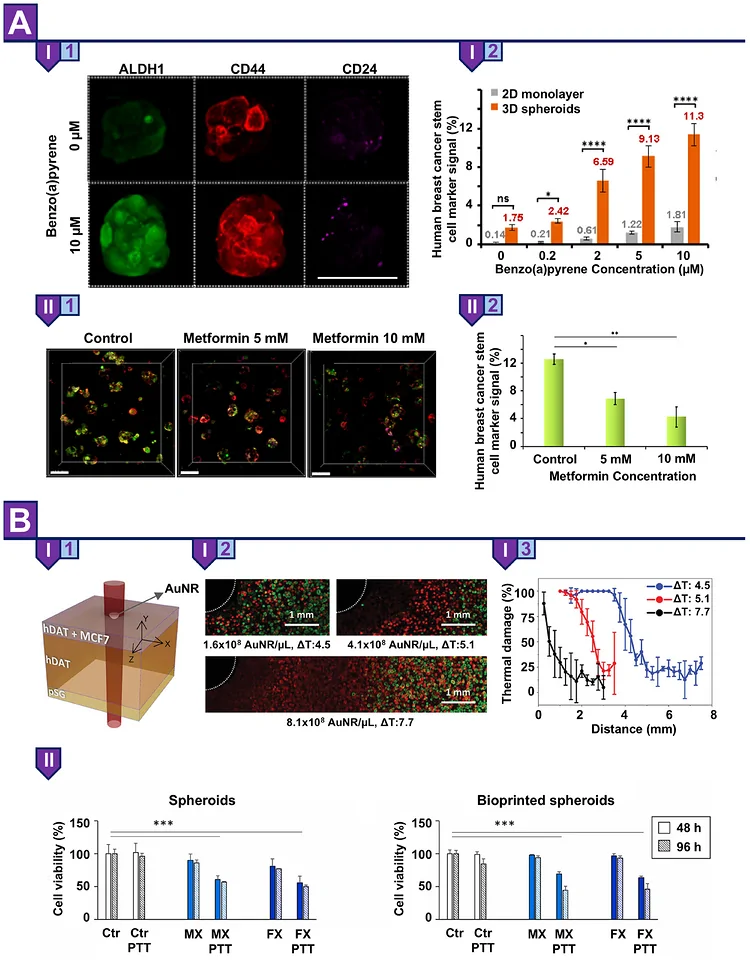
Investigating the applications of the first generation of 3DBP breast cancer models. (A) Drug testing investigations. (I) Carcinogenic drug benzo[a]pyrene induced mutation within 3D‐printed breast cancer spheroids. (1) Images showing cancer stem cell biomarker expressions in the spheroids. (2) Expression levels of the cancer stem cell biomarkers CD44, CD24, and ALDH1 in the spheroids. Cancer stem cells were enriched in a dose‐dependent manner with benzo[a]pyrene. (II) Effect of anticancer drug metformin on the benzo(a)pyrene‐induced cancer stem cell spheroids. (1) Images showing the CD44/ALDH1/CD24 biomarker signal of the spheroids. Scale bar 100 µm. (2) Dose‐dependent decrease in the cancer stem cell biomarker expression. (B) Investigating the potential of photothermal therapy with bioprinted 3D breast tissue constructs. (I) Cellular thermal damage in a bioprinted 3D complex tissue construct. (1) Schematic perspective and top view of the constructs. (2) Live/dead analysis of MCF‐7 cells. Live, green; dead, red. White dotted line indicates the gold nanorod‐loaded (AuNR) hemisphere edge. (3) Cellular thermal damage with laser irradiation as a function of the horizontal distance (on *X* and *Z* axes) from the AuNR hemisphere edge. Increased AuNR concentrations caused higher temperature variations over more distant areas, leading to cell death across wider areas. (II) Effect of Ti_3_C_2_T_x_ MXene‐mediated photothermal therapy on conventional and bioprinted 4T1 spheroids. Viability of conventional spheroids and bioprinted spheroids after photothermal therapy at 48 and 96 h after the administration of Mxenes demonstrated the greater effectiveness of photothermal exposure on the bioprinted spheroids. Ctr, control group; PTT, photothermal therapy; MX, multi‐layer Ti_3_C_2_T_x_ Mxenes; FX, few‐layer Ti_3_C_2_T_x_ Mxenes. Figure A reproduced with permission.[82] 2023, American Chemical Society. Figure B, (I) reproduced with permission.[83] 2021, John Wiley and Sons. Figure B, (II) reproduced from ref. [88] [This article is an open access article distributed under the terms and conditions of the Creative Commons Attribution (CC BY) license, https://creativecommons.org/licenses/by/4.0/].

Bioprinted constructs have also been used as photothermal‐treatment test beds. In an MCF‐7‐containing decellularized adipose‐tissue model, gold‐nanorod concentration, laser power, construct depth, temperature, and cellular damage were systematically related, enabling treatment parameters to be optimized within a multilayer tissue geometry (Figure 4B,I) [83]. A suspension of gold nanorods (AuNR) was syringe injected into the tissue‐construct. Following irradiation, AuNR generated localized heat and induced tumor cell death. In bioprinted 4T1 spheroids, Ti_3_C_2_T_x_ MXene‐mediated PTT produced a significantly greater reduction in cell viability than that seen in conventional spheroid models (Figure 4B, II) [88]. Enhanced thermal conduction by the ECM in the bioprinted constructs may explain this increased efficacy. Additionally, the treatment altered the behavior of immune‐related cells: macrophage migration increased, while endothelial cell infiltration was suppressed. These findings suggest that ECM composition and structure significantly influence not only thermal delivery but also immunomodulatory effects of PTT.

#### 3.1.5 Summary

Across cancer‐cell‐only platforms, the clearest contribution of bioprinting was experimental control rather than complete biological mimicry. Cell placement, aggregate dimensions, tumor geometry, matrix cues, and assay miniaturization were regulated with improved reproducibility. However, the resulting phenotypes were frequently influenced by interacting variables, including cell density, matrix composition and mechanics, molecular transport, crosslinking, and printing‐associated stress. The independent effects of architecture or fabrication could therefore rarely be established.

Cells in these models were generally more drug‐resistant than those in 2D culture, although the effect was model‐dependent rather than uniform. Sensitivity varied with cell source, geometry, material, treatment, exposure conditions, and analytical endpoint. These models are consequently most valuable as reductionist and standardized platforms for examining tumor‐intrinsic behavior and relative drug response. Their translational relevance remains restricted by the predominance of established cell lines, static culture, heterogeneous endpoints, and limited use of matched nonprinted 3D controls or patient‐derived cells (Table 4). These limitations provide the rationale for incorporating stromal, vascular, lymphatic, and immune components in subsequent model generations.

**TABLE 4:** Summary of first‐generation 3DBP breast cancer models.

Years	Cancer cells	Bioink	Printing/Crosslinking	Model complexity	Time	Drugs	Endpoint assays	Advantages/Limitations	Refs.
2015	MCF‐7	Cell‐laden 3% GEL sacrificial droplets; 20% PEG‐DMA microwell matrix + 0.1% photoinitiator	Pressure‐assisted valve droplet printing; GEL gelation at 4°C; PEG‐DMA UV crosslinking; GEL dissolution at 37°C	Bioprinting‐assisted, spheroid microwell array; spheroids self‐assembled after template dissolution	14 Days	None	Construct/Spheroid morphometry; viability	+Reproducible in situ seeding and tunable spheroid dimensions. −No treatment testing.	[74]
2019	MDA‐MB‐231	GEL/ALG transfer layer; ALG beads converted to liquid‐core, chitosan‐shelled capsules	Laser direct‐write deposition; CaCl_2_ ionic gelation; chitosan coating; ALG core liquefaction with citrate	Bioprinting‐assisted, size‐controlled spheroids in microcapsules	14 Days	None	Morphometry; viability; transport (transferrin)	+Precise spatial and size control. −Multistep processing reduced viability; no drug testing.	[39]
2019	MCF‐7	Sacrificial agarose fibers embedded in GelMA	Agarose extrusion; GelMA photocrosslinking; mechanical fiber removal; manual seeding into hollow channels	Bioprinting‐assisted, ductal carcinoma model with programmable, perfusable channel architecture	24 Days	None	Viability/Metabolism; invasion; ECM/EMT phenotype	+Programmable duct diameter, tortuosity and branching with long‐term invasion. −Heterogeneous manual seeding, limited mammary‐specific cues.	[75]
2019	MCF‐7 and MDA‐MB‐468; MCF‐12A	Self‐gelling human or rat mammary dECM; Geltrex and rat‐tail collagen control	Microextrusion of linear constructs; thermal self‐gelation at 37°C; no chemical crosslinker	Tumoroids in mammary dECM; nonmalignant mammary organoids included as control	14 Days	None	Growth/Morphology; histology; phenotype; gene/protein expression	+Tissue‐specific mammary cues and large organoids. −Source‐dependent matrix.	[79]
2020	MCF‐7	1% hydroxyethyl cellulose + 1% ALG + 5% GEL	Room‐temperature extrusion; physical pre‐gelation followed by ionic crosslinking	Directly bioprinted, in‐matrix spheroids; matched cast‐material control	15 Days	13 New drugs; doxorubicin	Viability/Metabolism; spheroid growth; phenotype (IF); IC_50_/SAR	+Low‐cost, room‐temperature printing with matched cast control. −Limited phenotypic marker panel.	[80]
2020	GFP‐expressing MDA‐MB‐231	ALG 1%, 3% or 5% × GEL 5%, 7% or 9% (AxGy formulations)	Extrusion through a 27G nozzle; room‐temperature pre‐gelation; ionic crosslinking	Directly bioprinted, disks with in‐matrix spheroids	28 Days	None	Print/Material QC; stiffness; spheroid growth/morphology; viability	+Separates alginate‐dominated stiffness from gelatin‐mediated adhesion over long culture. −No drug testing.	[85]
2020	MDA‐MB‐231	5% GelMA + 0.5% LAP photoinitiator	Electrohydrodynamic droplet printing; photocrosslinking	Directly bioprinted, 96‐well‐compatible 3D tumor array chip	7 Days	Epirubicin; paclitaxel	Viability/Metabolism; apoptosis/cell cycle; angiogenic/motility phenotype; IC_50_	+Highly uniform, miniaturized tumor arrays. −Comparisons only used 2D rather than matched nonprinted 3D controls.	[86]
2021	MCF‐7	Porcine‐skin gel + human adipose dECM; MCF‐7 embedded in dECM	Extrusion/Dispensing into a printed PCL mold; layerwise thermal gelation at 37°C	Tumor layer within a multilayer acellular dermis/hypodermis tissue analogue	Acute test	Gold nanorods	Thermography/Modeling; viability; thermal‐damage depth	+Quantitative photothermal dosimetry in a layered tissue geometry. −Acute endpoint.	[83]
2021	MDA‐MB‐231	1% ALG + 7% GEL, with or without 5% (v/v) Matrigel	Extrusion; room‐temperature pre‐gelation; ionic crosslinking; citrate chelation enabled recovery and reprinting	Spheroids	21 Days cycles (63 days)	Doxorubicin	Viability/Recovery; spheroid growth; histology/proliferation; dose–response (IC_50_/GR_50_)	+Reversible matrix and serial passaging. −Batch‐variable bioink.	[102]
2022	Bulk MCF‐7 and CD44‐selected MCF‐7	8% GEL + 3.2% ALG; polylactic‐acid miniwell chamber	Dual‐nozzle extrusion; cross‐shaped cell‐laden gel; ionic crosslinking	Directly bioprinted, miniwell spheroids; bulk versus resistance‐enriched cells	9 Days	Camptothecin; paclitaxel	Viability/Morphology; CSC/resistance phenotype; apoptosis/necrosis; EC_50_	+Quantitative, single‐spheroid drug‐response assay.	[40]
2022	MCF‐7; SK‐BR‐3; MDA‐MB‐231	Matrix: collagen–ALG–hyaluronic acid; tumor inks: GEL–hyaluronic acid with or without collagen	Custom extrusion into a printed matrix; collagen thermal gelation + ionic crosslinking; sacrificial gelatin/HA dissolution	Directly bioprinted, ductal, solid and co‐patterned tumor morphologies	10 Days	Doxorubicin; dasatinib; tirapazamine	Viability/Metabolism; histology; hypoxia/EMT/invasion	+Controlled histologic grade‐related morphology and spatial heterogeneity. −Geometry was confounded by cell density/bioink formulation.	[76]
2022	Murine 4T1	Cell suspension mixed 1:1 with 5% ALG (2.5% final)	Droplet printing into round‐bottom wells; ionic crosslinking	Directly bioprinted, spheroids; conventional spheroids as control; immune/endothelial assays were separate	30 Days	Ti_3_C_2_T_x_ MXene	Viability/Metabolism; ROS; spheroid growth; macrophage/endothelial migration	+Direct comparison with conventional spheroids and long‐term growth. −Murine cell line.	[88]
2022	ZR75.1	Cell gel: 3% ALG + 1% GEL; cell‐free scaffold gel: 6% ALG + 11% methylcellulose	Extrusion of alternating six‐layer structure; ionic crosslinking	Directly bioprinted; compared with 2D, ultra‐low‐attachment spheroids and xenograft	21 Days	Rapamycin; doxycycline; doxorubicin	Viability/Metabolism; growth; histology/phenotype; protein profiling; xenograft comparison	+Long‐term culture and cross‐model benchmarking.	[91]
2023	MCF‐7	8% GEL + 3.2% ALG	Dual‐mode extrusion of thin‐bar microconstructs; combined thermal gelation and ionic crosslinking	Directly bioprinted, high‐throughput single‐spheroid miniwell assay	9 Days	Benzo[a]pyrene (carcinogen); metformin	Viability/Morphology; genotoxicity; CSC phenotype	+In situ quantification of carcinogen‐induced CSC‐like enrichment. −No matched 2D or nonprinted 3D controls.	[82]
2023	Patient‐derived breast cancer cells/organoids	Bioprinted PEG with RGD, GFOGER and/or DYIGSR plus MMP‐degradable crosslinks	Noncontact drop‐on‐demand printing for PEG; GelMA UV‐crosslinked and GelSH click‐crosslinked as cast controls	Patient‐derived cancer organoids; only PEG constructs were bioprinted	22 Days	Doxorubicin; paclitaxel; EP31670	Viability/Metabolism; organoid growth; phenotype (IF); stiffness/rheology; IC_50_	+Patient‐derived cells with defined ligand and stiffness design. −Fabrication, matrix, ligands, crosslinking and organoid size were not uniform.	[89]
2023	MCF10AT	1% Hyaluronic acid + 1% elastin‐like protein; dynamic or static crosslinks; RGD versus nonbinding RDG	Extrusion/Embedded spatial printing; dynamic covalent self‐crosslinking; small‐molecule catalyst/competitor tuned gelation	Premalignant, cancer‐cell‐only invasion model with spatially patterned ligand cues	6 Days	TGF‐β (invasion inducer; not an anticancer drug)	Viability; rheology/Stress relaxation; print fidelity; invasion morphometry/IF	+Stiffness/Composition‐matched dynamic versus static matrices and spatial ligand control. −Premalignant line; no therapeutic test.	[90]
2025	Xenograft‐recovered MDA‐MB‐231 and MCF‐7	GelMA‐based photoink; 7% GelMA+ 2 mM/20 mM Ru/SPS photoinitiator	Custom DLP bioprinting; layer‐by‐layer photocrosslinking	Directly bioprinted, cancer‐cell‐only constructs with or without acellular blood‐vessel‐like channels	14 Days	Doxorubicin; capecitabine	Viability/Metabolism; morphology/phenotype; channel/print/mechanical QC; IC_50_; in vivo validation	+Rapid, high‐fidelity channels and preliminary in vitro/in vivo comparison. −Acellular, nonperfused channels.	[173]
2026	MDA‐MB‐231	Soft: 7.5% GelMA; stiff: 12.5% GelMA + 2.5% PEGDM; both with 0.2% LAP and 1 mM tartrazine	DLP bioprinting; photocrosslinking	Directly bioprinted, comparing compositionally distinct soft (7 kPa) and stiff (44 kPa) matrices	14 Days	Doxorubicin	Print/Material QC; viability/metabolism; morphology; secretome; cell mechanics (AFM); drug response	+Links matrix mechanics, cell stiffening and DOX response.	[172]

Abbreviations: AFM, atomic force microscopy; ALG, alginate; AxGy, *x*% ALG–*y*% GEL formulation; CD44, cluster of differentiation 44; CSC, cancer stem cell; dECM, decellularized extracellular matrix; DLP, digital light processing; DYIGSR, Asp–Tyr–Ile–Gly–Ser–Arg; EC50, half‐maximal effective concentration; ECM, extracellular matrix; EMT, epithelial–mesenchymal transition; GEL, gelatin; GelMA, gelatin methacryloyl; GelSH, thiolated gelatin; GFOGER, Gly–Phe–Hyp–Gly–Glu–Arg; GFP, green fluorescent protein; GR50, half‐maximal growth‐rate inhibition concentration; HA, hyaluronic acid; IC50, half‐maximal inhibitory concentration; IF, immunofluorescence; LAP, lithium phenyl‐2,4,6‐trimethylbenzoylphosphinate; MMP, matrix metalloproteinase; PCL, polycaprolactone; PEG, poly(ethylene glycol); PEG‐DMA/PEGDM, poly(ethylene glycol) dimethacrylate; QC, quality control; RDG, Arg–Asp–Gly; RGD, Arg–Gly–Asp; ROS, reactive oxygen species; Ru/SPS, ruthenium/sodium persulfate photoinitiator system; SAR, structure–activity relationship; TGF‐β, transforming growth factor beta; Ti3C2Tx, surface‐terminated titanium carbide MXene.

### 3.2 Second Generation: Models Containing Two Cell Types

Second‐generation models were formed by combining breast cancer cells with one additional supporting cell type, enabling heterotypic interactions absent from cancer‐cell‐only constructs to be examined. MCF‐7 [32, 35, 36, 38], MDA‐MB‐231 [33, 35, 39, 101], MDA‐MB‐468 [32], and 21PT [37] were combined with fibroblasts (IMR‐90 [33]), adipocytes [35, 36], ASC‐derived spheroids [101], adipose‐derived mesenchymal stem cells (ADMSCs) [37, 38], embryonic stem cells [39], and breast cancer stem cells [40].

These co‐cultures were produced using alginate [35, 39], alginate/gelatin composites [33, 36, 40], collagen and collagen type I [38], hyaluronic acid [101], and decellularized delipidated tumor ECM combined with gelatin methacryloyl (GelMA) and alginate [38]. Cell–cell and cell–matrix interactions could thereby be examined [101], and more in vivo‐like behavior [37] and treatment responses [35, 37, 40], including retention of MCF‐7 chemoresistance [40], were demonstrated.

#### 3.2.1 Cancer and Epithelial Cells

Non‐tumorigenic mammary epithelial cells were incorporated to examine whether a normal epithelial context could modify breast cancer cell organization and phenotype. MCF‐7 or MDA‐MB‐468 cells were combined with MCF‐12A cells at a 1:5 ratio and deposited as 66‐nL, 40‐cell clusters in linear or circular arrays within pre‐polymerized rat‐tail collagen I (Figure 5A) [32]. Compared with random manual embedding, the reported frequency of chimeric organoid formation was increased from ∼1% to ∼96% at Day 7 and from ∼3% to ∼90% at Day 14. Large contiguous structures containing both cell types were formed by Day 21. The principal contribution of printing was therefore controlled initial cell proximity and reproducible chimeric‐organoid generation.

**FIGURE 5:**
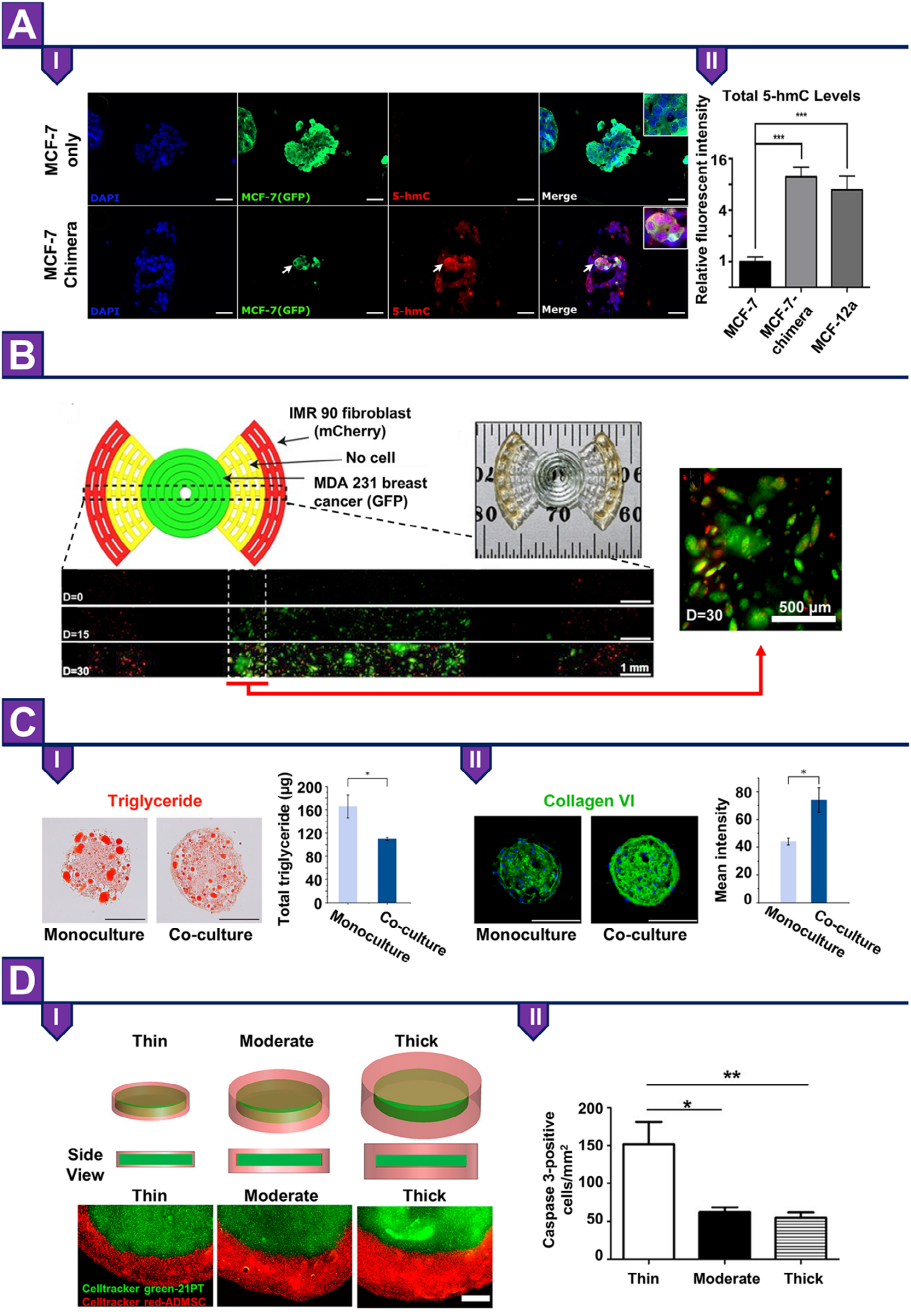
Second generation of 3DBP breast cancer models that employ two different cell types. (A) Cancer cell and epithelial cell‐based constructs. Epigenetic alterations in tumorigenic MCF‐7 cells in chimeric organoids (with non‐tumorigenic MCF‐12A cells). (I) Images of bioprinted constructs 21 days post‐printing. Scale bar 50 µm. (II) Incorporation into chimeric organoids causes significantly increased 5‐hydroxymethylcytosine (5‐hmC) levels in MCF‐7 cells. Reduced 5‐hmC levels are characteristic of cancer cells. (B) Cancer cell and fibroblast‐based constructs. Multicellular tumor spheroid formation in 3DBP alginate/gelatin constructs throughout 30 days. IMR‐90 fibroblasts (red) migrated across the no‐cell region and infiltrated MDA‐MB‐231 tumor spheroids. (C) Cancer cell and adipocyte‐based constructs. Differences between monocultures of human adipose‐derived stromal cell spheroids and their co‐cultures with MDA‐MB‐231 breast cancer cells on Day 30 (Day 9 of co‐culture). Scale bar 100 µm. Addition of MDA‐MB‐231 cells caused a significant reduction in lipid (I) and an increase in collagen VI content (II) content in the spheroids. (D) Cancer cell and stem cell‐based constructs. Effect of an ADMSC layer on drug resistance. (I) 3DBP breast cancer models with various ADMSC outer layer thicknesses. Scale bar 500 µm. (II) Caspase‐3‐positive cell density across constructs with different ADMSC outer‐layer thicknesses. Decreased density of caspase‐3‐positive cells indicates reduced doxorubicin activity on 21PT breast cancer cells in the middle area of the constructs. Figure A was reproduced from ref. [32] (an open‐access article distributed under the terms of the Creative Commons CC BY license). Figure B was reproduced from ref. [33] (an open‐access article distributed under the terms of the Creative Commons Attribution License, CC BY 4.0). Figure C was reproduced from ref. [101] [This article is an open access article distributed under the terms and conditions of the Creative Commons Attribution (CC BY) license, https://creativecommons.org/licenses/by/4.0/]. Figure D reproduced with permission.[37] 2018, American Chemical Society.

Subtype‐dependent effects were observed. MCF‐7 cells were incorporated into lumen‐containing structures, and their 5‐hydroxymethylcytosine levels (typically low in malignant phenotypes) were increased to values comparable to those of MCF‐12A cells. In contrast, MDA‐MB‐468 cells remained dispersed and were not observed lining lumens [32]. Although the matrix used lacked human mammary‐specific stromal cues, these findings underscore the significance of incorporating tissue‐specific cellular contexts in 3DBP tumor models.

#### 3.2.2 Cancer Cells and Fibroblasts

Fibroblasts are major stromal regulators of tumor growth, organization, and treatment response [174, 175, 191, 192], while the tumor–stroma ratio has also been associated with clinical outcome [193]. Bioprinting has therefore been used to regulate the initial distance, distribution, and matrix context of tumor and stromal populations, allowing their subsequent migration and phenotypic changes to be examined under defined conditions.

An early spatially patterned model was generated by separately embedding MDA‐MB‐231 cells and IMR‐90 fibroblasts in 3% alginate–7% gelatin and printing a central tumor region and peripheral fibroblast regions separated by an acellular gap (Figure 5B) [33]. Tumor spheroids appeared after 7 days, and fibroblasts crossed the gap and infiltrated them by approximately Day 15; heterogeneous spheroids were maintained for 30 days. Thus, the starting geometry was defined by printing, whereas the eventual tumor–stromal organization arose through migration and self‐assembly. Although the IMR‐90 fibroblasts used are not breast‐derived CAFs, the model demonstrated a dynamic migration and integration phenomenon.

Proteolytic matrix–stroma coupling was examined using a BCN‐functionalized HA bioink supplemented with 1% xanthan gum and cRGD and crosslinked with either an MMP‐degradable VPM peptide or nondegradable PEG bis‐azide [141]. MCF‐7 cells were printed in a heart‐shaped compartment and covered with a separately printed layer containing primary human dermal fibroblasts, and the constructs were cultured for 14 days. Fibroblasts promoted tumor‐cell growth and spheroid formation, but the largest increases in cell density and spheroid size, together with migration from the original tumor compartment, occurred when the MCF‐7 region was protease‐degradable. The factorial comparison of fibroblast‐free and fibroblast‐containing degradable/nondegradable compartments therefore showed that stromal crosstalk and matrix susceptibility acted jointly.

Tissue‐specific matrix cues were incorporated through flow‐on‐repellent extrusion onto a superhydrophobic surface. MDA‐MB‐231 cells and human breast CAFs at a 1:2 tumor‐to‐stroma ratio were deposited as photocrosslinked beads in 2.5% HAMA containing 5% porcine mammary dECM microfibers [174]. During 7 days of culture, tumor cells remained broadly dispersed, whereas CAFs formed clusters. Following 72‐h gemcitabine exposure, cell survival was higher in heterotypic than monotypic conditions. CAF inclusion was associated with an upward shift in survival, with the greatest resistance observed in heterotypic breast cancer beads.

High‐throughput dot printing was used to compare adipose‐dECM microspheres containing MCF‐7 cells alone (MC), MCF‐7/MRC‐5 co‐cultures (MCR), or co‐cultures treated with TGF‐β to induce a CAF‐like phenotype (MCRT) [175]. Fibroblast co‐culture increased construct growth and partially activated fibroblasts, while TGF‐β further intensified tumor–stromal interactions. By Day 7, metabolic activity increased 3.5‐fold in MCR versus 2.1‐fold in MC, and the α‐SMA‐positive fraction increased from 6.1% in MC to 30.9% in MCR and 76.3% in MCRT. MCRT also exhibited greater migration, including an increased contribution of migrating cancer cells, larger and more proliferative tumor spheroids, denser ECM deposition, and elevated HIF‐1α. Reduced E‐cadherin and increased Snail, Vimentin, and Twist1 indicated an EMT‐associated shift, while IL‐6, IL‐8, TNF‐α, and VEGF secretion was enhanced. Transcriptomic analysis of MCF‐7 cells from MCRT identified changes in ECM organization, chemotaxis, PI3K–Akt signaling, cytokine pathways, and drug‐resistance genes. Correspondingly, resistance to doxorubicin, oxaliplatin, and gemcitabine increased, whereas sensitivity to 5‐fluorouracil was enhanced. Although the findings were limited by the use of lung‐derived MRC‐5 fibroblasts, they demonstrate that CAF‐like activation promoted a more migratory, hypoxic, and drug‐modulated phenotype rather than uniform chemoresistance.

Spatiotemporal monitoring of drug uptake and response within 3DBP breast cancer models is critical for capturing intratumoral heterogeneity and tumor–stroma differences that are inaccessible to conventional end‐point assays. In a recent study, gold nanorod‐loaded alginate/carboxymethylcellulose pillars were directly printed into core–shell bioprinted breast cancer constructs and used as surface‐enhanced Raman scattering‐based sensors for real time monitoring of drug transport and consumption. The model was fabricated using porcine breast‐derived dECM containing MDA‐MB‐231 tumor cells in the core and human breast fibroblasts in the stroma. Upon treatment with the anticancer drug 6‐thioguanine, the sensor pillars revealed a significantly faster decline in drug signal in the tumor core than in the stroma over 5 days, correlating with higher tumor cell death [176].

#### 3.2.3 Cancer Cells and Adipocytes

Adipocytes are abundant components of mammary stroma and can acquire cancer‐associated phenotypes characterized by lipid loss and altered ECM secretion, thereby supporting breast cancer progression and invasion [194, 195]. Bioprinting has been used to model this interaction through either direct admixture of tumor and adipose cells or sequential assembly of spatially separated compartments.

An initial proof‐of‐concept model was produced by mixing MCF‐7 cells and adipose‐derived stromal cell (ADSC)‐derived adipocytes at a 1:1 ratio in a 3:2 alginate‐to‐gelatin blend prepared with 5% alginate [36]. Cylindrical constructs were extrusion‐printed and ionically crosslinked. The viability of both cell types was maintained throughout the 10‐day culture period. Compared to monocultures, the 3DBP co‐cultures exhibited more clustered cell distributions, suggesting enhanced cell–cell and cell–matrix interactions.

A more structured bioprinting‐assisted hybrid model was generated from preformed human ASC spheroids suspended in a photocrosslinkable thiolated‐hyaluronic‐acid bioink containing allyl‐functionalized poly(glycidol) and high‐molecular‐weight hyaluronic acid (Figure 5C) [101]. The spheroids were extrusion‐printed, differentiated toward adipose microtissues for 21 days, and overlaid with a separately printed MDA‐MB‐231‐containing hydrogel compartment for a further 9 days of co‐culture. At the selected printing condition of 1 bar through a 330‐µm nozzle, visibly damaged spheroids increased from 1% in matched cast controls to 9%; damage reached 56% under the more severe condition of 5 bar and a 250‐µm nozzle. Nevertheless, differentiation and ECM deposition were comparable between printed and cast adipose constructs, indicating that printing primarily enabled homogeneous spheroid distribution and controlled compartmental integration rather than inducing adipogenesis. During co‐culture, tumor cells remained confined to their compartment, while lipid and triglyceride content decreased within the adipose microtissues. Collagen I, collagen VI, and fibronectin deposition increased, collagen IV decreased, and laminin remained unchanged relative to adipose‐only controls [101]. Because direct contact and tumor‐cell migration between compartments were absent, the findings are most consistent with paracrine adipose remodeling.

#### 3.2.4 Cancer Cells and Adipose‐Derived Mesenchymal Stromal Cells (ADMSCs)

ADMSCs can modify breast cancer progression and treatment response through paracrine signaling and ECM remodeling. Bioprinting has been used to organize these cells within layered or granular tumor compartments, enabling stromal geometry, matrix mechanics, and tissue‐specific matrix cues to be examined.

A radially organized construct was produced by printing HER2‐positive 21PT cells in a central compartment and primary human ADMSCs in surrounding layers with various thicknesses (0.4, 0.8, or 1.2 mm) using methacrylated‐hyaluronic‐acid‐ and gelatin‐based bioinks (Figure 5D) [37]. After 21 days of culture and 3 days of doxorubicin exposure, fewer cleaved‐caspase‐3‐positive cells were detected in co‐cultures than in cancer‐cell‐only constructs at 1 and 10 µg mL^−1^, whereas 100 µg mL^−1^ caused extensive apoptosis in both. Moderate and thick ADMSC layers were associated with less apoptosis than the thin layer. Lysyl oxidase inhibition reduced stiffness selectively within the ADMSC region and increased doxorubicin sensitivity. Although the model enabled a simplified assessment of stromal‐thickness‐dependent drug response, its relevance to obesity‐associated breast cancer was limited by the absence of mature adipocytes and obesity‐associated metabolic and inflammatory cues.

An anatomically patterned model was generated by printing MCF‐7 cells and human ADMSCs in spatially distinct compartments using porcine mammary‐dECM bioinks containing GelMA and alginate, with or without collagen I [177]. In cancer‐cell‐only constructs, mammary dECM promoted stemness‐, invasion‐, and immunosuppression‐associated phenotypes but did not independently increase doxorubicin resistance, while collagen I supplementation increased COL1A1, CDH2, and MMP2 expression. Incorporation of ADMSCs increased doxorubicin resistance in the heterotypic model, particularly when collagen I was included, indicating that stromal and matrix composition jointly influenced treatment response. However, their individual contributions were not resolved. Translational relevance was further limited by the absence of vascular, immune, patient‐derived, and in vivo validation.

Matrix architecture and tumor–stromal crosstalk were examined more explicitly using ADMSC‐laden GelMA microgel matrices with low or high packing densities. MCF‐7 or MDA‐MB‐231 cells were deposited at the matrix interface by drop‐on‐demand printing in 96‐ or 384‐well plates [133]. Within 72 h, MDA‐MB‐231 cells migrated up to approximately 1000 µm through high‐packing matrices containing ADMSCs, compared with less than 350 µm without ADMSCs. Greater α‐SMA expression and morphological changes consistent with a myofibroblastic CAF‐like phenotype were observed in high‐packing co‐cultures with both cancer cell lines. In MDA‐MB‐231 drug assays, ADMSC inclusion increased the doxorubicin IC_50_ from ∼1.3 to ∼1.9 µM in low‐packing matrices and from ∼3.6 to ∼5.5 µM in high‐packing matrices [133]. Similar doxorubicin distributions across the matrices reduced the likelihood that these differences resulted solely from drug penetration.

True tumor‐to‐stroma invasion was subsequently enabled with a low‐polymer, migration‐permissive bioink containing ColMA and HA‐SH [178]. GFP‐labelled MDA‐MB‐231 cells and human ASCs were extrusion‐printed in contiguous tumor and stromal compartments and photocrosslinked together, producing a unified collagenous matrix without an interfacial barrier. During 95 h of 3D live imaging, ASCs increased tumor‐cell track length and speed, promoted a more elongated phenotype and collagen remodeling, and increased the fraction of tracks entering the stromal compartment from 7% in the matched acellular control to ∼17%. The design therefore extended the earlier ring–disk model from paracrine migration to quantified intercompartmental invasion while controlling initial geometry and matrix conditions [178].

#### 3.2.5 Cancer and Endothelial Cells

A bipartite tumor–endothelial model was generated using matrix‐assisted sacrificial printing. A petroleum‐jelly/liquid‐paraffin fugitive ink was printed within a 0.6% bacterial‐cellulose hydrogel, which was subsequently dried and processed to form hollow, perfusable channels. GFP‐labeled HUVECs were then introduced into the channels, while MCF‐7 cells were seeded onto both surfaces of the surrounding cellulose matrix [179]. Printing controlled the channel geometry and spatial compartmentalization, whereas endothelial lining and tumor‐cell growth occurred after seeding. By Day 14, HUVECs formed a confluent channel lining, whereas MCF‐7 cells proliferated along the matrix surfaces but did not penetrate the dense cellulose network.

#### 3.2.6 Cancer Cells and Bone Marrow‐Derived Mesenchymal Stromal Cells

A bone‐marrow stromal compartment was incorporated to evaluate localized therapy at a breast cancer–bone interface [180]. MDA‐MB‐231 cells and primary human bone marrow‐derived mesenchymal stromal cells (hBM‐MSCs) were separately suspended in 1% alginate–7% gelatin and extrusion‐printed as a 2.6‐mm‐diameter tumor disk surrounded by a 4.4‐mm‐diameter stromal ring, both 1 mm high. A reversed arrangement was also fabricated to control for compartment position. The constructs were ionically crosslinked. After 24 h, the constructs were exposed once to direct cold atmospheric plasma (CAP) for varying durations or to 5 µM doxorubicin. Live/dead analysis showed greater loss of MDA‐MB‐231 viability than hBM‐MSC viability with CAP exposure, whereas doxorubicin affected both populations similarly.

#### 3.2.7 Summary

Second‐generation models introduced one supporting cell population, allowing defined pairwise interactions to be examined. The designation therefore indicates an intermediate level of cellular complexity rather than chronological or technical superiority (Table 5). Printing contributed most clearly by controlling initial cell proximity, compartment geometry and thickness, matrix architecture, sensor placement, or channel configuration. In several bioprinting‐assisted hybrid models, only the starting architecture was imposed by printing, whereas mature organization subsequently arose through migration, differentiation, self‐assembly, or post‐print seeding. These biological processes should therefore be distinguished from the spatial control provided by printing. The strongest functional evidence was obtained in fibroblast‐ and ADMSC‐containing models, in which migration, CAF‐like activation, hypoxia‐ and EMT‐associated changes, ECM remodeling, altered mechanics, and modified drug responses were demonstrated. Nevertheless, treatment effects were model‐ and drug‐dependent rather than uniformly resistant, while sensing and localized‐treatment platforms remained proof‐of‐concept demonstrations. Epithelial and adipocyte models revealed subtype‐dependent organization, phenotypic modulation, and paracrine remodeling, whereas the endothelial construct primarily established spatially controlled channel lining. Cross‐study interpretation was limited by established cancer cell lines, non‐mammary supporting cells in some models, short or unequal culture periods, bulk assays, and simultaneous variation in matrix composition, stiffness, cell number, geometry, or diffusion distance. Patient‐derived tumors, immune components, sustained perfusion with validated vascular function, and in vivo validation were generally absent. Future pairwise models should therefore employ tissue‐matched primary cells, matched monoculture and acellular controls, cell‐resolved longitudinal endpoints, and standardized concentration–response testing. These models have potential to provide a mechanistically tractable bridge between cancer‐cell‐only constructs and more complex multicellular systems.

**TABLE 5:** Summary of second‐generation 3DBP breast cancer models.

Years	Cancer cells	Supporting cell population	Bioink	Printing/Crosslinking	Model complexity	Time	Drugs	Endpoint assays	Advantages/Limitations	Refs.
2017	MDA‐MB‐231	IMR‐90	3% ALG + 7% GEL	Multicartridge extrusion; room‐temperature pre‐gelation; ionic crosslinking	Directly bioprinted tumor core and peripheral fibroblast regions separated by an acellular gap; migration‐driven mixed spheroids.	30 Days	None	Viability; spheroid morphometry; fibroblast migration/infiltration	+Defined starting geometry and long‐term heterotypic self‐assembly. −Non‐mammary fibroblasts; no stromal‐activation or treatment testing.	[33]
2018	21PT	Primary human ADMSCs	MeHA/HA and MeGel/GEL dual bioinks	Dual‐head extrusion of a tumor core and concentric stromal layers; photocrosslinking	Directly bioprinted radial model with tunable ADMSC‐layer thickness.	21 Days	Doxorubicin; LOX inhibitor	Viability; apoptosis (cleaved caspase‐3); stiffness; LOX secretion; gene expression	+Links stromal geometry/mechanics with drug response.	[37]
2019	MCF‐7; MDA‐MB‐468	MCF‐12A	Pre‐polymerized rat‐tail collagen I	Robotic microdispensing into defined patterns; no post‐print crosslinking	Bioprinting‐assisted chimeric mammary organoids; mature organization formed by post‐print self‐assembly.	21 Days	None	Organoid morphometry; live imaging/migration; histology/IF; 5‐hmC	+Reproducible cell proximity and chimeric‐organoid generation. ‐Collagen‐only matrix and no drug testing.	[32]
2019	MCF‐7	GFP‐HUVECs	Bacterial‐cellulose matrix; petroleum‐jelly/liquid‐paraffin sacrificial ink	Matrix‐assisted sacrificial extrusion; drying/processing and fugitive‐ink removal; cells post‐seeded	Bioprinting‐assisted endothelialized hollow‐channel/tumor interface.	14 Days	Tamoxifen	Channel/Perfusion QC; viability; endothelial lining; qualitative drug response	+Low‐cost, perfusable channel. −Post‐seeded static model; no angiogenic function; one‐dose qualitative drug test.	[179]
2020	MCF‐7	ADSC‐derived adipocytes	ALG/GEL blend (3:2; prepared with 5% ALG)	Extrusion of cylindrical constructs; ionic crosslinking	Directly bioprinted mixed tumor–adipocyte co‐culture.	10 Days	None	Material QC; viability/metabolism; DNA; lipid staining; morphology	+Simple direct tumor–adipocyte proof of concept. −Bulk/Qualitative assays were not cell‐resolved; no drug testing.	[36]
2021	MDA‐MB‐231	Human ASC spheroids differentiated into adipose microtissues	Thiolated HA + allyl‐functionalized poly(glycidol) + high‐MW HA	ASC‐spheroid extrusion and photocrosslinking; separate tumor compartment added for co‐culture	Bioprinting‐assisted hybrid with differentiated adipose microtissue and a spatially separate tumor compartment.	21 Days	None	Print damage/Viability; adipogenesis/lipids; ECM IF; compartment morphology	+Mature adipose microtissue and controlled paracrine interface. −No direct contact or invasion; no drug testing.	[101]
2022	MCF‐7	Human ADMSCs	Porcine mammary dECM + GelMA + ALG, with or without collagen I	Extrusion of a 2‐layer tumor core and 4‐layer stromal shell; photocrosslinking + ionic crosslinking	Directly bioprinted core–shell construct.	14 Days	Doxorubicin	Print/Material QC; viability/proliferation; histology/IF; gene expression; IC_50_	+Tissue‐specific dECM and sharp tumor–stroma interface. −Co‐culture was assessed for only 1 days.	[38]
2024	MCF‐7	Primary human dermal fibroblasts from breast‐reduction skin	HA‐BCN + 1% XG + cRGD; MMP‐degradable or nondegradable crosslinker	Pneumatic extrusion; SPAAC crosslinking; printed MCF‐7 compartment covered by a fibroblast layer	Directly bioprinted, spatially separated tumor–fibroblast model with factorial degradable/nondegradable compartments.	14 Days	None	Material/Degradation QC; viability; confocal morphology; cancer‐cell density; spheroid size/compactness; migration	+Fibroblast‐free controls and factorial matrix degradability reveal matrix–stroma coupling. −Dermal rather than mammary fibroblasts; no drug testing.	[141]
2025	MCF‐7; MDA‐MB‐231	Human ADMSCs	GelMA granular microgel matrices with low or high packing density	Granular‐matrix assembly and drop‐on‐demand cancer‐cell deposition in well plates; GelMA photocrosslinking	Bioprinting‐assisted, high‐throughput tumor–stroma interface in tunable microporous matrices.	72 h	Doxorubicin	Matrix QC; migration; α‐SMA/CAF‐like phenotype; secretome; viability/IC_50_; drug distribution	+Compares matrix architecture and stromal inclusion at high throughput.	[133]
2025	MDA‐MB‐231	Human breast CAFs	2.5% HAMA with or without 5% porcine mammary‐dECM microfibers	Flow‐on‐repellent extrusion onto a superhydrophobic bed; photocrosslinking	Directly bioprinted quasispherical tumor–CAF beads with tissue‐specific fibrous dECM.	7 Days	Gemcitabine	Print/Bead QC; viability; cell tracking/morphology; drug‐response viability	+Rapid, reproducible fibrous‐dECM tumor–stroma beads. −Drug readout was not cell‐resolved.	[174]
2025	MCF‐7	MRC‐5 lung fibroblasts, with or without TGF‐β‐induced CAF‐like activation	Adipose‐dECM/GelMA hybrid bioink	High‐throughput extrusion dot printing into well plates; photocrosslinking	Directly bioprinted mixed microspheres with an inducible CAF‐like stromal state.	7 Days	Doxorubicin; oxaliplatin; gemcitabine; 5‐fluorouracil; TGF‐β inducer	Viability/Metabolism; flow/IF; migration/spheroids; ECM/hypoxia/EMT; cytokines/transcriptomics; IC_50_	+Rapid well‐plate model with dynamic stromal activation and broad drug testing. −Lung‐derived fibroblasts; bulk assays.	[175]
2025	MDA‐MB‐231	Human breast fibroblasts	Porcine breast dECM; Au‐nanorod‐loaded thiolated ALG/methacrylated CMC sensing‐pillar ink	Multimaterial extrusion of a dECM core–shell model and sensing pillars; dECM thermal gelation + pillar thiol–ene photocrosslinking	Directly bioprinted tumor–stroma compartments with printed, compartment‐specific sensors.	5 Days	6‐thioguanine	Print/Sensor QC; compartment‐resolved longitudinal SERS; viability/cell death	+Local, longitudinal monitoring of drug dynamics.	[176]
2025	MCF‐7	Human ADMSCs	Porcine mammary dECM+ GelMA + ALG, with or without collagen I	Extrusion of tumor and stromal compartments; photocrosslinking + ionic crosslinking	Directly bioprinted tumor–ADMSC model separating tissue‐specific matrix and stromal compartments.	14 Days	Doxorubicin	Print/Material QC; viability/growth; stemness, invasion, immune and ECM phenotype; IC_50_	+Anatomically patterned, tissue‐specific matrix–stroma model.	[177]
2025	MDA‐MB‐231	Primary human bone‐marrow‐derived MSCs	1% ALG + 7% GEL	Extrusion of a tumor disk and stromal ring, ionic crosslinking	Directly bioprinted tumor–bone‐marrow‐stroma interface.	24 h	CAP; doxorubicin control	Spatial live/Dead imaging	+Spatial assessment of selective plasma response.	[180]
2026	MDA‐MB‐231‐GFP	Human ASCs	3.5 mg mL^−^^1^ ColMA + 1 mg mL^−^^1^ HA‐SH + 0.8 mg mL^−^^1^ LAP; same matrix in both compartments	Two‐compartment extrusion; photocrosslinking	Fully bioprinted contiguous tumor–ASC interface with a matched acellular stromal compartment and no physical barrier.	5 Days	Batimastat; Y‐27632 (mechanistic spheroid assays)	Print/Material QC; viability; 3D single‐cell tracking; invasion; morphology; collagen remodeling; MMP/protease assays	+Matched acellular control and true intercompartmental invasion resolved at single‐cell level. −Short culture time.	[178]

Abbreviations: 5‐hmC, 5‐hydroxymethylcytosine; ADMSC, adipose‐derived mesenchymal stromal cell; ADSC/ASC, adipose‐derived stromal cell; ALG, alginate; Au, gold; BCN, bicyclo[6.1.0]nonyne; CAF, cancer‐associated fibroblast; CMC, carboxymethylcellulose; ColMA, methacrylated collagen; dECM, decellularized extracellular matrix; DNA, deoxyribonucleic acid; ECM, extracellular matrix; EMT, epithelial–mesenchymal transition; GEL, gelatin; GelMA, gelatin methacryloyl; GFP, green fluorescent protein; HA, hyaluronic acid; HAMA, hyaluronic acid methacrylate; HA‐SH, thiolated hyaluronic acid; HUVEC, human umbilical vein endothelial cell; IC_50_, half‐maximal inhibitory concentration; IF, immunofluorescence; LAP, lithium phenyl‐2,4,6‐trimethylbenzoylphosphinate; LOX, lysyl oxidase; MeGel, methacrylated gelatin; MeHA, methacrylated hyaluronic acid; MMP, matrix metalloproteinase; MSC, mesenchymal stromal cell; MW, molecular weight; QC, quality control; SERS, surface‐enhanced Raman scattering; SPAAC, strain‐promoted azide‐alkyne cycloaddition; TGF‐β, transforming growth factor‐β; XG, xanthan gum; α‐SMA, α‐smooth muscle actin.

### 3.3 Third Generation: Multiple‐Cell‐Type Models That Include Cancer and Other Cell Types

Third‐generation 3DBP models extend beyond pairwise co‐cultures by integrating multiple, functionally interacting components of the breast TME within spatially organized constructs. Through the controlled arrangement of tumor, stromal, vascular, lymphatic, and immune elements, these models enable interdependent tissue‐level processes and treatment responses to be examined with greater physiological relevance.

#### 3.3.1 Modeling Vascularized Breast Cancer

Vascularization is a major determinant of breast tumor growth, metastatic dissemination, and therapeutic exposure because endothelial cells mediate oxygen, nutrient, and drug transport while also exchanging paracrine signals with tumor and stromal cells [71]. In the 3DBP literature, however, the term “vascularized” encompasses different levels of functionality. Some models contain spatially organized endothelial cells or CD31‐positive cord‐like structures, whereas others generate lumenized networks; only a limited number demonstrate perfusion or measurable barrier function. These constructs should therefore be interpreted along a continuum from prevascular organization to functional, perfusable vasculature rather than considered equivalent merely because endothelial cells are present.

An early scaffold‐free approach used continuous‐deposition bioprinting to construct a breast tumor core containing MCF‐7 cells and human umbilical vein endothelial cells (HUVECs), surrounded by a stromal compartment composed of primary human mammary fibroblasts and HUVECs (Figure 6) [46]. Temporary hydrogel components were removed after printing, allowing the cells to produce their own ECM. The endothelial cells developed branched CD31‐positive capillary‐like networks, while modification of the stromal composition produced distinct tissue phenotypes. Mesenchymal stromal cells increased collagen deposition and desmoplastic organization without substantially increasing MCF‐7 proliferation, whereas preadipocytes underwent maturation within the construct. These results demonstrated that stromal identity can alter ECM remodeling and tissue architecture independently of tumor‐cell number. The same platform also illustrated how multicellular organization can modify therapeutic response [46]. In tissues containing mammary fibroblasts, HUVECs, preadipocytes, and MCF‐7 cells, the doxorubicin median lethal dose was approximately 0.66 µM, compared with 0.032 µM in 2D cultures containing the same cell proportions. In HCC1143‐containing tissues, the PI3K/mTOR inhibitor BEZ235 suppressed ribosomal protein S6 phosphorylation but did not produce a corresponding reduction in proliferation, and fibroblast‐conditioned medium attenuated the treatment response. In MDA‐MB‐231‐containing tissues, sunitinib disrupted endothelial networks while simultaneously increasing collagen deposition.

**FIGURE 6:**
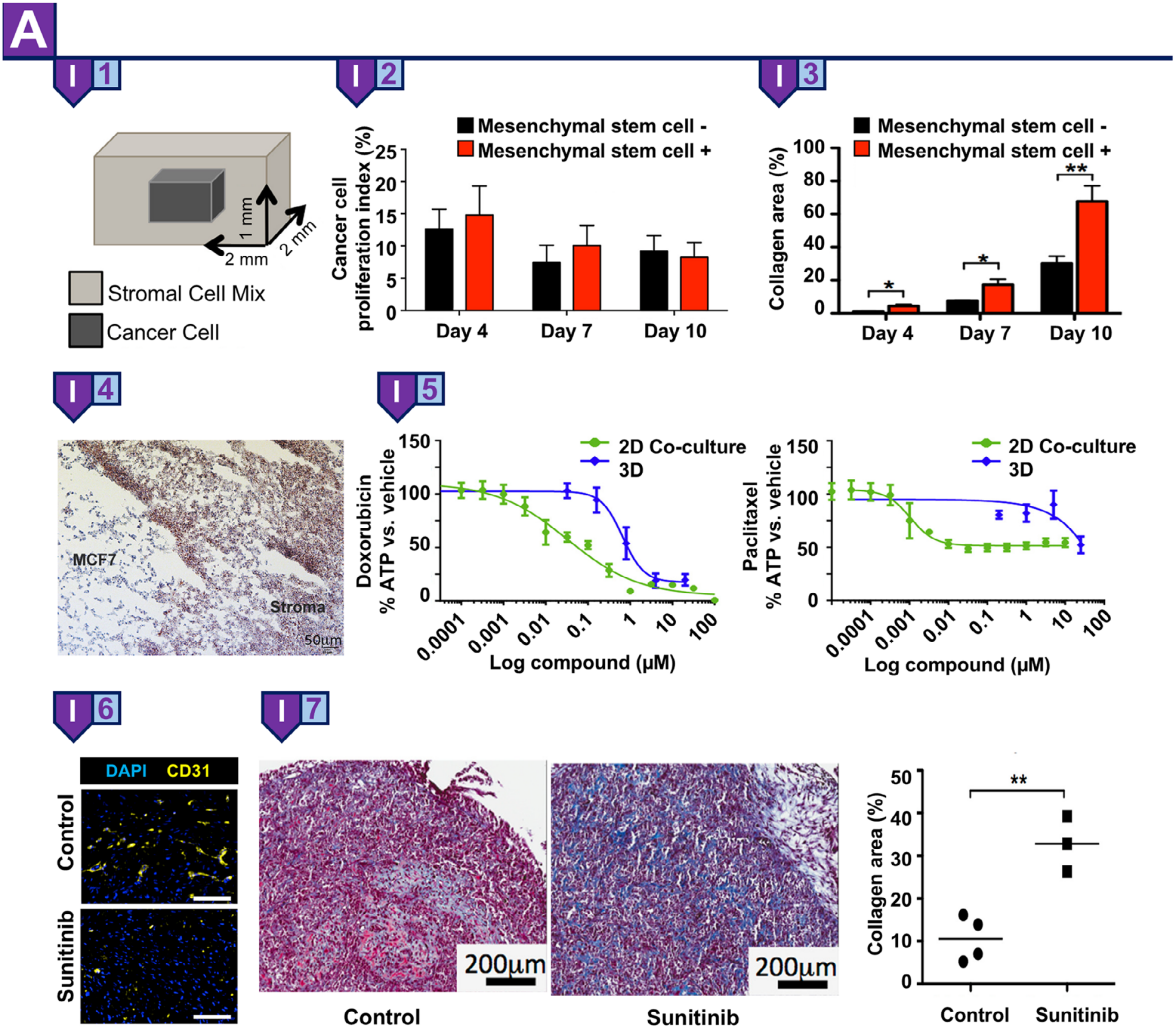
Third generation of 3DBP breast cancer models that employ multiple additional cell types. (A) Tumor stroma with additional tissue‐relevant cell types. (I) Effect of MSCs or adipocytes in stroma with fibroblasts and endothelial cells. (1) A schematic of a 3DBP tissue. Stroma included primary human mammary fibroblasts (50%) and HUVECs (25%) and an additional cell type (25%). Core solely consisted of MCF‐7 (unless stated otherwise). (2) Quantitation of the percentage of Ki‐67‐positive MCF‐7 cells in the constructs with bone‐marrow‐derived MSCs. (3) Quantitation of trichrome staining on the bioprinted tissues with MSC. ∗*p* < 0.05 and ∗∗*p* < 0.01 by a two‐tailed *t* test. While the presence of MSCs did not have an effect on cell proliferation, they caused a significant increase in collagen deposition resulting in a more desmoplastic TME. (4) Oil red O staining of cryosectioned bioprinted breast cancer tissues containing primary human subcutaneous preadipocytes (SPA). SPA could be differentiated into mature adipocytes and increase the lipid content of stroma. Scale bar 50 µm. (5) Dose response curves for 3DBP MCF‐7 breast cancer tissues containing preadipocytes, compared to 2D co‐cultures with the same ratio of cell types. Tissues or cells were treated with doxorubicin or paclitaxel and assessed by ATP utilization assay. Significantly higher drug doses were required to show efficacy on the cancer cells in the 3DBP constructs with adipocytes. (6) Immunofluorescent images of tissues bioprinted with MDA‐MB‐231. Bioprinted tissues were left untreated (control) or treated with 1 µM Sunitinib. Sunitinib inhibited vascular endothelial growth factor and reduced the endothelial cell networks. Scale bar 100 µm. (7) Representative images (left) and quantitation (right) of trichrome staining in MDA‐MB‐231 bioprinted tissues treated with or without Sunitinib. Sunitinib demonstrating side effects by increasing collagen deposition in the stroma, thereby increasing stiffness. ∗∗*p* < 0.01 by a two‐tailed *t* test. Figure A was reproduced from ref. [46] [This article is an open access article distributed under the terms and conditions of the Creative Commons Attribution (CC BY‐NC‐ND 4.0), license, https://creativecommons.org/licenses/by‐nc‐nd/4.0/].

Greater control over spatial organization was achieved using a multibarrel nozzle combined with aerosol‐mediated crosslinking to deposit four adjacent bioinks with different cellular and mechanical properties (Figure 7A) [44]. MDA‐MB‐231 cells were localized within a cancer layer, HUVECs and normal human lung fibroblasts were incorporated into a vascular layer, and fibroblast‐containing migration and support layers separated and stabilized the construct. By Day 12, the endothelial cells had formed hollow CD31‐positive network structures, while tumor cells migrated through the intervening layers toward the vascular region. Although the construct primarily reproduced prevascular morphogenesis rather than functional tumor vasculature, the model demonstrated how bioprinting can establish reproducible interfaces for examining tumor migration toward an endothelial compartment.

**FIGURE 7:**
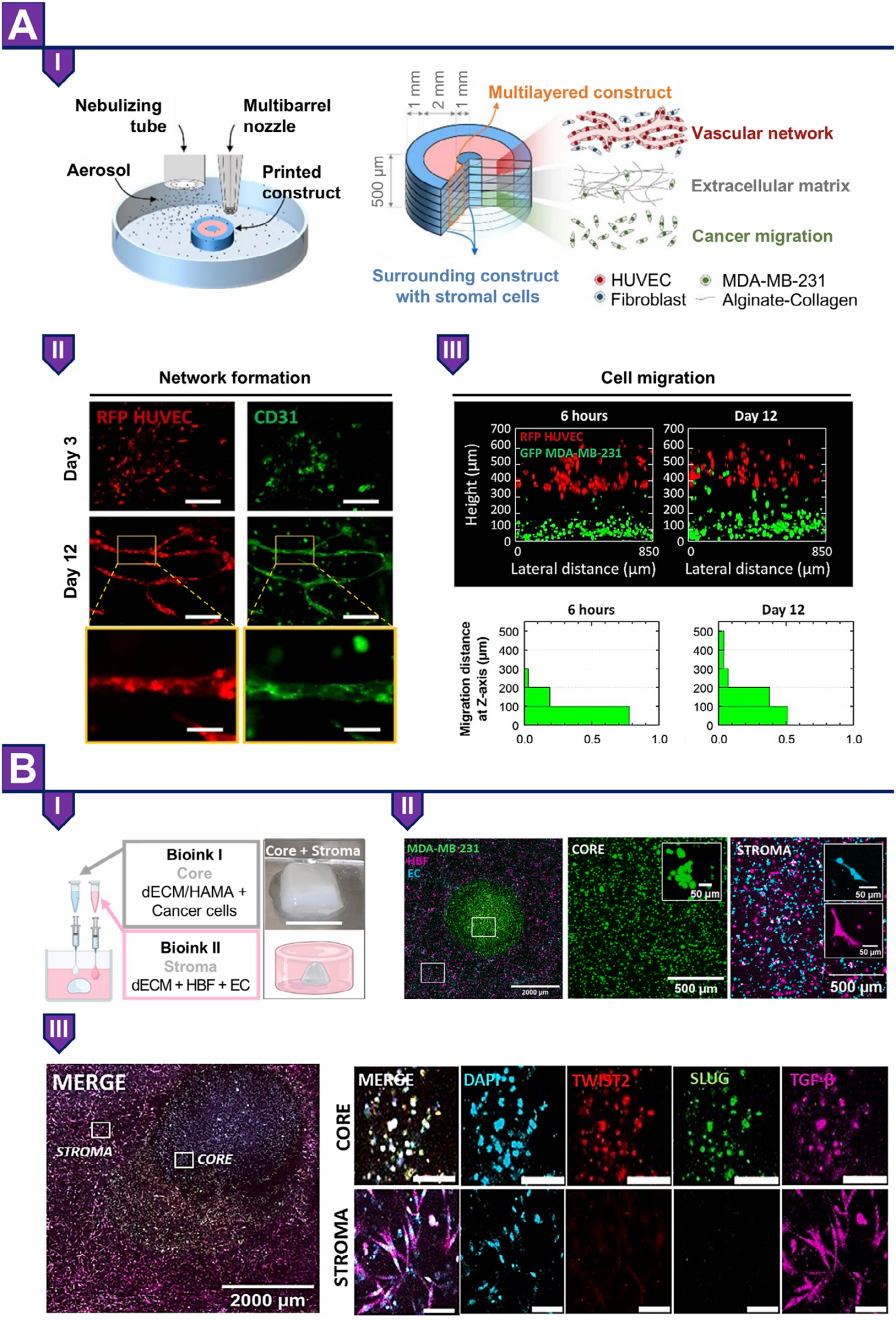
Third generation of 3DBP breast cancer models that employ more than two cell types. (A) Multilayered and heterogeneous constructs with different mechanical properties and cellular composition. (I) A schematic of printing the constructs with multiple layers: a cancer cell layer, a stromal cell layer and a vascular layer, all enclosed by a stromal layer. (II) HUVECs in the vascular layer formed hollow network structures with high CD31 tight cell‐cell junction protein expression in 12 days. RFP; red fluorescent protein. (III) MDA‐MB‐231 cells migrated through layers with different mechanical properties and reached the vascular layer over 12 days of incubation. GFP; green fluorescent protein. (B) Constructs with cancer, fibroblast and endothelial cells. Modeling the complexity of breast tumors with architectural features resembling the breast TME. (I) Two distinct bioinks were prepared for tumor core (dECM, porcine breast‐derived decellularized extracellular matrix; HAMA, methacrylated hyaluronic acid; cancer cells, human triple‐negative MDA‐MB‐231 cells) and tumor stroma (dECM; HBF, human stromal breast fibroblasts; EC, endothelial cells) to resemble the breast tumor niche. Scale bar 2 mm (core) and 1 cm (stroma). (II) Fluorescence images depicting an inner core composed of MDA‐MB‐231 breast tumor cells (green), surrounded by HBF (pink) and EC (cyan) stromal cells. While the cells in the core region formed typical tumor aggregates, cells in the stromal region demonstrated elongated fibroblast and endothelial cell morphologies. (III) Immunofluorescent images of the 3D model stained for TWIST 2 (red), SLUG (green), and TGF‐β (pink) and with DAPI (cyan) for cell nuclei. MDA‐MB‐231 cells within the tumor core expressed SLUG and TWIST‐1 biomarkers, which indicate EMT (aggressive phenotype) progression in the core region. Scale bar 100 µm. Figure A reproduced with permission.[44] 2021, IOP Publishing. Figure B was reproduced from ref. [43] [This article is an open access article distributed under the terms and conditions of the Creative Commons Attribution (CC BY‐NC‐ND 4.0), license, https://creativecommons.org/licenses/by‐nc‐nd/4.0/].

Matrix composition and oxygen tension were examined using embedded bioprinting in a physically crosslinked silk‐fibroin support bath [42]. A tumor core containing 21PT breast cancer cells in a collagen–hyaluronic acid–pNIPAM bioink was surrounded by HUVECs and immortalized fibroblasts embedded in a collagen I–fibrin matrix. Culture under hypoxic conditions increased endothelial network formation and tumor‐cell migration from the core into the surrounding vascularized matrix. This behavior was accompanied by increased expression of VEGFA, matrix metalloproteinases, and lysyl oxidase‐like 2. The results highlighted the interdependence of oxygen availability, ECM remodeling, angiogenic signaling, and tumor invasion.

Tissue‐specific biochemical signaling was incorporated by bioprinting tumor cores composed of either MCF‐7 or MDA‐MB‐231 cells in porcine breast‐derived decellularized ECM and hyaluronic acid methacrylate (Figure 7B) [43]. The cores were surrounded by a stromal compartment containing primary human breast fibroblasts and human aortic endothelial cells. MDA‐MB‐231‐containing constructs exhibited increased expression of EMT‐associated factors, including SLUG and TWIST1, together with evidence of TGF‐β signaling, while endothelial clustering and CD31 expression suggested angiogenic organization. Drug testing in a multiwell format showed dose‐related MCF‐7 responses to doxorubicin, paclitaxel, and tamoxifen. In contrast, MDA‐MB‐231 constructs displayed greater resistance and a less consistent dose–response relationship, particularly for tamoxifen. Following high‐dose tamoxifen exposure, cell death was concentrated near the outer region of the tumor core, consistent with diffusion‐limited drug exposure.

A more recent model extended these compartmentalized approaches by using multi‐nozzle extrusion bioprinting to spatially organize MDA‐MB‐231 cells, endothelial cells, and human mammary cancer‐associated fibroblasts (CAFs) within biomimetic ECM bioinks [182]. Dense cancer‐cell‐rich regions were surrounded by microvessel‐rich stroma, while angiogenic organization and ECM stiffening were localized predominantly within the stromal compartment, resembling patterns observed in patient specimens. Transcriptomic analysis identified angiogenesis‐ and ECM‐remodeling signatures together with candidate ligand–receptor interactions between the cellular compartments. Cancer cells occupying different spatial regions also exhibited unequal chemotherapy sensitivity, directly connecting bioprinted spatial heterogeneity with environment‐mediated drug resistance.

Overall, most current vascularized breast tumor constructs reproduce endothelial organization or prevascular morphogenesis rather than perfusable, barrier‐forming vasculature, making functional transport validation the principal remaining challenge.

#### 3.3.2 Modeling Breast Cancer With Lymphatics

Lymphatic vessels provide a major route for regional breast cancer dissemination and influence interstitial transport within tumors [196, 197, 198]. Nevertheless, lymphatic compartments remain substantially less represented than blood vasculature in 3DBP breast cancer models. Although only two cell populations were used in the model described below, its inclusion within this generation is justified by the spatial reconstruction of a distinct lymphatic compartment rather than by cell number alone.

A sacrificial extrusion‐bioprinting strategy was used to generate a lymphatic‐vessel‐containing triple‐negative breast cancer model (Figure 8A) [41]. An agarose microfiber was printed between partially photocrosslinked layers of GelMA and subsequently removed to produce a hollow channel. Human dermal LECs were seeded within the channel, while MDA‐MB‐231 cells were encapsulated in the surrounding GelMA matrix. By Day 8, the LECs had formed an intact cylindrical CD31‐positive lining. Treatment with VEGF‐C promoted LEC sprouting over 20 days and enhanced MDA‐MB‐231 migration toward and along the lymphatic structure. Thus, printing defined the initial channel geometry and tumor–lymphatic separation, whereas subsequent sprouting and directional migration arose through cell‐mediated organization.

**FIGURE 8:**
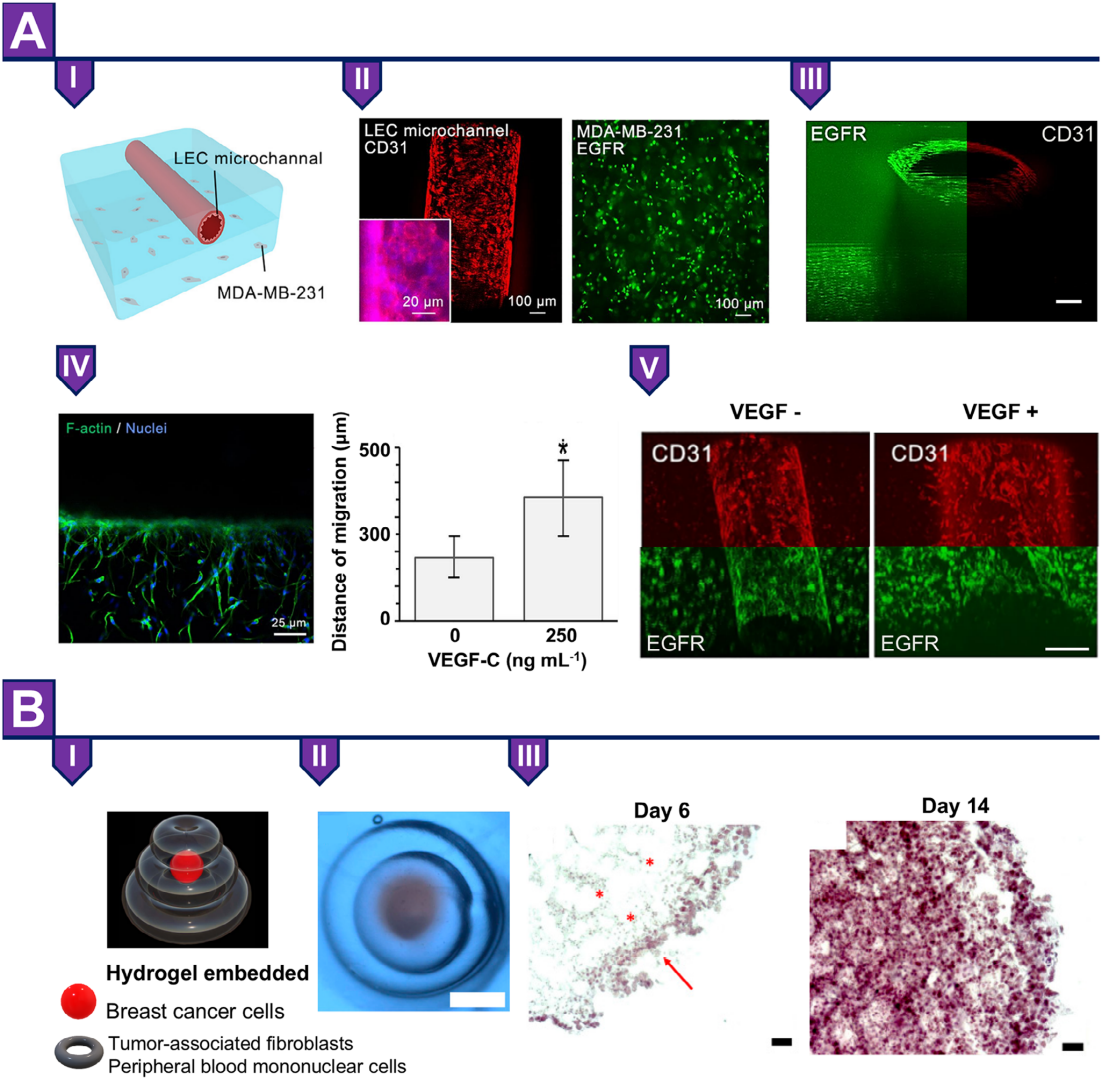
Third generation of 3DBP breast cancer models that employ multiple additional cell types. (A) Modeling breast cancer having lymphatics. I) Constructs with microchannels were created with sacrificial agarose microfibers embedded in photocrosslinked GelMA hydrogel. LECs were seeded into the microchannel and MDA‐MB‐231 cells were encapsulated in the polymeric matrix. (II) Confocal reconstruction images showing the LECs (left) and MDA‐MB‐231 breast tumor cells at Day 8 of culture. The inset is a high‐magnification image showing CD31 expressions, where CD31 was stained in purple and nuclei in blue. (III) Cross section view of intact cylindrical lymphatic vessel. LECs in the microchannel and MDA‐MB‐231 cells in the matrix at Day 8 of culture. Scale bar 100 µm. (IV) Effect of VEGF‐C (250 ng mL^−1^) on lymphangiogenesis. F‐actin and CD31 staining of the LECs, revealing sprouting at 20 days of culture. (V) MDA‐MB‐231 cell migration toward the microchannel to bridge the initial gap in between the two cell types. The migration behavior of the cells was pronounced with VEGF exposure. (B) Modeling breast cancer having immune cells. (I) Digital models of the triple‐layered structure that mimic the TME. Tori represent rings of hydrogel loaded with human tumor‐associated fibroblasts and peripheral blood mononuclear cells, whereas the red sphere stands for a cancer‐cell‐laden hydrogel droplet encapsulating SK‐BR‐3 breast cancer cells. (II) Stereomicroscopic top view of 3DBP triple‐layered structures. Scale bar 1 mm. (III) Hematoxylin‐eosin staining of the triple‐layered tissue constructs. Significant proliferation, migration and remodeling occurred in the constructs, which were facilitated by the proximity of the fibroblasts and mononuclear cells. Stars indicate cellular distribution on the inner part; arrows point to the exterior part of the structure, which is intensely populated with cells. Scale bar 10 µm. Figure A reproduced with permission.[41] 2021, Elsevier. Figure B was reproduced from ref. [45] (an open‐access article distributed under the terms of the Creative Commons Attribution License, CC BY 4.0).

However, lymphatic function and therapeutic performance were not demonstrated. No anti‐lymphangiogenic agent was tested, and lymph‐like flow, drainage, permeability, barrier function, or tumor‐cell intravasation was not quantified. Biological breadth was further limited by the use of dermal rather than breast‐ or tumor‐derived LECs, and the absence of stromal and immune populations. Overall, the model reproduced early tumor–lymphatic contact and lymphangiogenic sprouting but not the transport, immune, and metastatic functions of a lymphatic system.

Recent non‐bioprinted 3D lymphatic models provide a useful functional benchmark for future biofabricated systems. In defined synthetic hydrogels, human LECs and bone‐marrow‐derived mesenchymal stromal cells self‐organized into matrix‐supported lymphatic networks. Invasive MDA‐MB‐231 cells associated more closely with these networks than MCF‐7 cells, and this interaction was attenuated by CCL21 blockade [199]. Although outside the directly bioprinted study set, these findings identify stromal support, subtype‐dependent lymphatic interactions, and perturbable chemokine signaling as priorities for future printed lymphatic models.

#### 3.3.3 Modeling Breast Cancer With Fibroblasts and Immune Cells

Direct incorporation of both fibroblasts and immune cells remains uncommon in 3DBP breast cancer models. In one compartmentalized approach, SK‐BR‐3 breast cancer cells were combined with primary breast tumor‐associated fibroblasts (TAFs) and peripheral blood mononuclear cells (PBMCs) in a commercial nanocellulose–alginate bioink (Figure 8B) [45]. TAFs and PBMCs from HLA‐A*11‐positive breast cancer patients were selected for compatibility with the SK‐BR‐3 cell line, thereby reducing nonspecific immune responses caused by major histocompatibility mismatch. Using two extrusion‐printing cartridges, SK‐BR‐3 cells were deposited as a central tumor compartment, while an equal mixture of TAFs and PBMCs was printed in surrounding hydrogel rings. Both toroidal and pyramidal triple‐layered constructs were produced, with the latter providing greater structural stability and more complete enclosure of the tumor compartment. During 14 days of static culture, the constituent cells proliferated, migrated, formed heterotypic aggregates, and remodeled the hydrogel [45]. Triple‐layered constructs developed denser and more consistent cellular organization than toroidal constructs. Cells accumulated preferentially near the construct periphery, which was attributed to spatial differences in oxygen and nutrient availability. Thus, bioprinting established the initial tumor–stromal architecture, whereas subsequent cell‐mediated migration and self‐organization generated the evolving cellular microstructure. However, TAFs and PBMCs were deposited together and were not distinguished during histological analysis. Consequently, immune‐cell survival, infiltration, activation, and interactions with either fibroblasts or cancer cells were not resolved.

A recent bioprinting‐assisted vascularized model advanced from static immune‐cell inclusion toward dynamic immune–stromal interrogation [183]. Heterotypic spheroids containing HCC1937 triple‐negative breast cancer cells and fibroblasts were incorporated into a self‐organizing endothelial–stromal matrix within a perfused bioreactor. Following vascular‐network development, allogeneic PBMCs were introduced through the circulation. PBMC perfusion rapidly reduced vascular density and interconnectivity and increased tumor‐cell apoptosis and pyroptosis, resulting in spheroid shrinkage. Nevertheless, peripheral tumor‐cell proliferation and invasive dissemination into the surrounding matrix were simultaneously enhanced. Fibroblast activation, FAP and αvβ3 integrin expression, and collagen I/III deposition also increased, indicating immune‐induced desmoplastic remodeling. Myeloid and macrophage‐like populations progressively predominated over cytotoxic T cells, linking the observed response primarily to innate inflammatory activity rather than a sustained tumor‐specific adaptive response [183].

#### 3.3.4 Summary

Third‐generation 3DBP breast cancer models are distinguished not simply by the number of incorporated cell types, but by the spatial organization and functional interaction of tumor, stromal, vascular, lymphatic, and immune components (Table 6). Their maturity can be evaluated at three levels: cellular incorporation, structural organization, and functional integration. Although most reported models reproduce the first two levels, comparatively few demonstrate perfusion, vascular barrier function, lymphatic drainage, defined immune activity, or treatment responses.

**TABLE 6:** Summary of third‐generation 3DBP breast cancer models.

Years	Cancer cells	Supporting cell populations	Bioink	Printing/Crosslinking	Model complexity	Time	Drugs	Endpoint assays	Advantages/Limitations	Refs.
2019	MCF‐7; SK‐BR‐3; HCC1143; MDA‐MB‐231	Primary human mammary fibroblasts; HUVECs; preadipocytes and/or bone‐marrow MSCs	High‐density cellular paste with removable temporary hydrogel support; cell‐produced ECM in the mature tissue	Continuous‐deposition extrusion; temporary hydrogel removed; no permanent scaffold crosslinking	Directly bioprinted tumor core surrounded by stromal/endothelial regions; self‐produced ECM and branched CD31+ capillary‐like networks; nonperfused.	10 Days	Doxorubicin; paclitaxel; BEZ235; sunitinib	ATP/Luciferase viability; histology/IF; target engagement; endothelial‐network, migration and ECM morphometry; SHG	+Scaffold‐free multicellular platform with subtype‐ and stroma‐dependent drug/ECM responses. −No perfusion; heterogeneous endpoints.	[46]
2021	MDA‐MB‐231	Human dermal lymphatic endothelial cells	GelMA tumor matrix; sacrificial agarose microfiber	Sacrificial agarose extrusion between photocrosslinked GelMA layers; fiber removal; LEC post‐seeding; final photocrosslinking	Bioprinting‐assisted endothelialized hollow lymphatic channel separated from tumor cells; VEGF‐C‐induced sprouting and directional migration; static/no flow.	20 Days	VEGF‐C stimulation	Confocal/IF (CD31, F‐actin); channel/lumen integrity; LEC sprouting; cancer‐cell migration	+Controlled tumor–lymphatic geometry and long‐term sprouting. −Dermal LECs; no flow or drug testing.	[41]
2021	SK‐BR‐3	Primary breast tumor‐associated fibroblasts; patient PBMCs (HLA‐A*11‐selected)	Commercial nanocellulose–alginate	Dual‐cartridge extrusion of toroidal and triple‐layered pyramidal constructs; ionic crosslinking	Directly bioprinted central tumor compartment enclosed by fibroblast/PBMC rings; static, avascular model with post‐print migration and self‐organization.	14 Days	None	Viability; Hoechst staining/histology; spatial cell distribution and aggregate morphology; computational self‐organization	+Patient‐derived stromal/immune cells and defined compartmental architecture. −No immune‐function, treatment, or vascular assays.	[45]
2021	MDA‐MB‐231	HUVECs; normal human lung fibroblasts	RGD–alginate/collagen I (cancer and migration layers); RGD–alginate/fibrinogen (vascular layer); 8% alginate support	Continuous multi‐bioink microextrusion through a multibarrel nozzle; CaCl_2_ aerosol and ionic post‐crosslinking	Directly bioprinted four‐layer cancer/vascular/migration/support construct; hollow CD31+ networks and cancer migration toward the vascular region; nonperfused.	12 Days	None	Print/Interface quality; cancer‐cell migration; CD31 IF; lumen and endothelial‐network morphometry	+Precise multicompartment interfaces and lumen‐like endothelial networks. −Lung fibroblasts; no perfusion or drug testing.	[44]
2023	21PT	HUVECs; immortalized human fibroblasts (H16NF)	Tumor core: collagen I + HA–pNIPAM; vascular matrix: collagen I + fibrin; physically crosslinked silk‐fibroin support bath	Embedded extrusion in silk‐fibroin bath; thermal collagen/HA–pNIPAM gelation and thrombin‐mediated fibrin gelation	Directly printed tumor core within endothelial/fibroblast matrix; hypoxia enhanced vascular organization and tumor‐cell migration; nonperfused.	7 Days	None	Viability/Morphology; CD31 network IF/morphometry; cancer‐cell migration; RNA‐seq/qPCR; hypoxia‐response markers	+Low‐collagen embedded printing links hypoxia, angiogenesis and invasion. −No perfusion or drug testing.	[42]
2023	MCF‐7; MDA‐MB‐231	Primary human breast fibroblasts; human aortic endothelial cells	Tumor core: porcine breast dECM + 2% HAMA; stroma: unmodified porcine breast dECM	Sequential extrusion; UV photocrosslinking of tumor core and thermal gelation of stromal dECM; 48‐well format	Directly bioprinted tumor core–stroma model with tissue‐specific ECM; endothelial clustering/CD31+ organization; nonperfused.	7 Days	Doxorubicin; paclitaxel; tamoxifen	Live/Dead; LDH cytotoxicity; histology/IF; cytokine array; EMT/TGF‐β/CAF markers; spatial drug‐response imaging	+Breast‐specific dECM, subtype comparison and plate‐compatible spatial drug testing. −No perfusion; short drug assay.	[43]
2024	MDA‐MB‐231	HUVECs; human mammary CAFs	Region‐optimized GelMA + 5% gelatin ECM‐mimetic bioinks	Multi‐nozzle extrusion; photocrosslinking	Directly bioprinted cancer‐cell‐rich regions within microvessel‐rich fibroblast/endothelial stroma; spatial angiogenesis, ECM stiffening and drug resistance; nonperfused.	7 Days	Doxorubicin	Live/Dead; CD31/ECM/CAF IF; vessel morphometry; stiffness mapping; FACS and transcriptomics/ligand–receptor analysis; regional drug response	+Patient‐image‐inspired spatial heterogeneity with mechanistic omics and region‐resolved drug response. −No perfusion.	[182]
2026	HCC1937	Normal human dermal fibroblasts; GFP‐HUVECs; allogeneic PBMCs	Collagen–fibrinogen matrix; sacrificial gelatin channel ink	Bioprinting‐assisted hybrid: sacrificial gelatin channel printing, HUVEC post‐seeding, embedded tumor–fibroblast spheroids; perfusion at 0.1 mL min^−^^1^	Perfusable endothelialized vessel connected to a self‐organizing vascular network infiltrating heterospheroids; circulating PBMC challenge.	15 Days	None	Live imaging/Vessel morphometry; IF; SHG collagen; cytokine profiling; flow cytometry of immune subsets; invasion and spheroid size	+Functional perfusion and dynamic immune–vascular–stromal analysis. −Allogeneic mismatch.	[183]

Abbreviations: dECM, decellularized extracellular matrix; GelMA, gelatin methacryloyl; HAMA, methacrylated hyaluronic acid; HMCAFs, human mammary cancer‐associated fibroblasts; HUVECs, human umbilical vein endothelial cells; IF, immunofluorescence; LECs, lymphatic endothelial cells; MSCs, mesenchymal stromal cells; PBMCs, peripheral blood mononuclear cells; SHG, second‐harmonic generation.

The evidence remains uneven across the modeled compartments. Blood‐vascular models have achieved multicompartment tumor–stroma organization, endothelial cord or lumen formation, hypoxia‐dependent angiogenesis and invasion, and spatially heterogeneous drug responses. However, most represent prevascular organization rather than perfusable and barrier‐forming vasculature. Lymphatic modeling remains limited to an endothelialized sacrificially printed channel that supports VEGF‐C‐induced sprouting and directional tumor‐cell migration, without demonstrating drainage, lymph‐like flow, permeability, or tumor‐cell intravasation. Immune modeling has progressed from the static incorporation of fibroblasts and heterogeneous peripheral blood mononuclear cells to immune‐cell perfusion through vascularized hybrid constructs. Notably, immune exposure was shown to increase tumor‐cell death while simultaneously promoting invasion and desmoplastic remodeling, indicating that viability alone may provide an incomplete or misleading measure of therapeutic response.

Interpretations remain constrained by frequent reliance on established cancer cell lines, non‐breast‐derived endothelial cells, dermal LECs, allogeneic or incompletely characterized immune populations, static culture, short observation periods, and heterogeneous outcome measures. Future models should integrate blood and lymphatic networks with perivascular and stromal cells, defined autologous or HLA‐matched immune subsets, and controlled perfusion. Vascular validation should include lumen continuity, perfusability, and permeability, whereas lymphatic assessment should include drainage, directional transport, permeability, and tumor‐cell intravasation. Immune‐cell survival, migration, subset identity, activation state, cytokine signaling, cytotoxicity, antigen presentation, and immune‐checkpoint activity should be evaluated alongside tumor viability, invasion, and ECM remodeling.

### 3.4 Metastasis Models

Breast cancer metastasis is an organ‐selective, multistep process, with bone, lung, liver, and brain representing the principal distant sites; among these, bone is the most frequently involved across molecular subtypes [200]. Because individual platforms reproduce different portions of the metastatic cascade, they should be interpreted according to whether they capture local invasion, vascular dissemination, extravasation, or colonization of a secondary tissue. 3DBP enables spatially controlled construction of primary‐tumor, vascular, and organ‐specific niches, allowing tumor–stromal and tumor–ECM interactions to be investigated under more representative conditions. Early models primarily examined cancer‐cell adaptation and crosstalk within the target tissue, whereas subsequent vascularized systems incorporated migration between primary and secondary compartments.

An early bone‐metastasis bioprinting‐assisted hybrid model was produced by stereolithography‐based bioprinting of human fetal osteoblasts or human bone marrow‐derived MSCs within GelMA/nHAp matrices, followed by seeding of MDA‐MB‐231 cells onto the printed constructs (Figure 9A) [92]. Co‐culture increased cancer‐cell proliferation and vascular endothelial growth factor secretion while reducing the proliferation and alkaline phosphatase activity of the bone‐forming cells, demonstrating reciprocal tumor–bone crosstalk.

**FIGURE 9:**
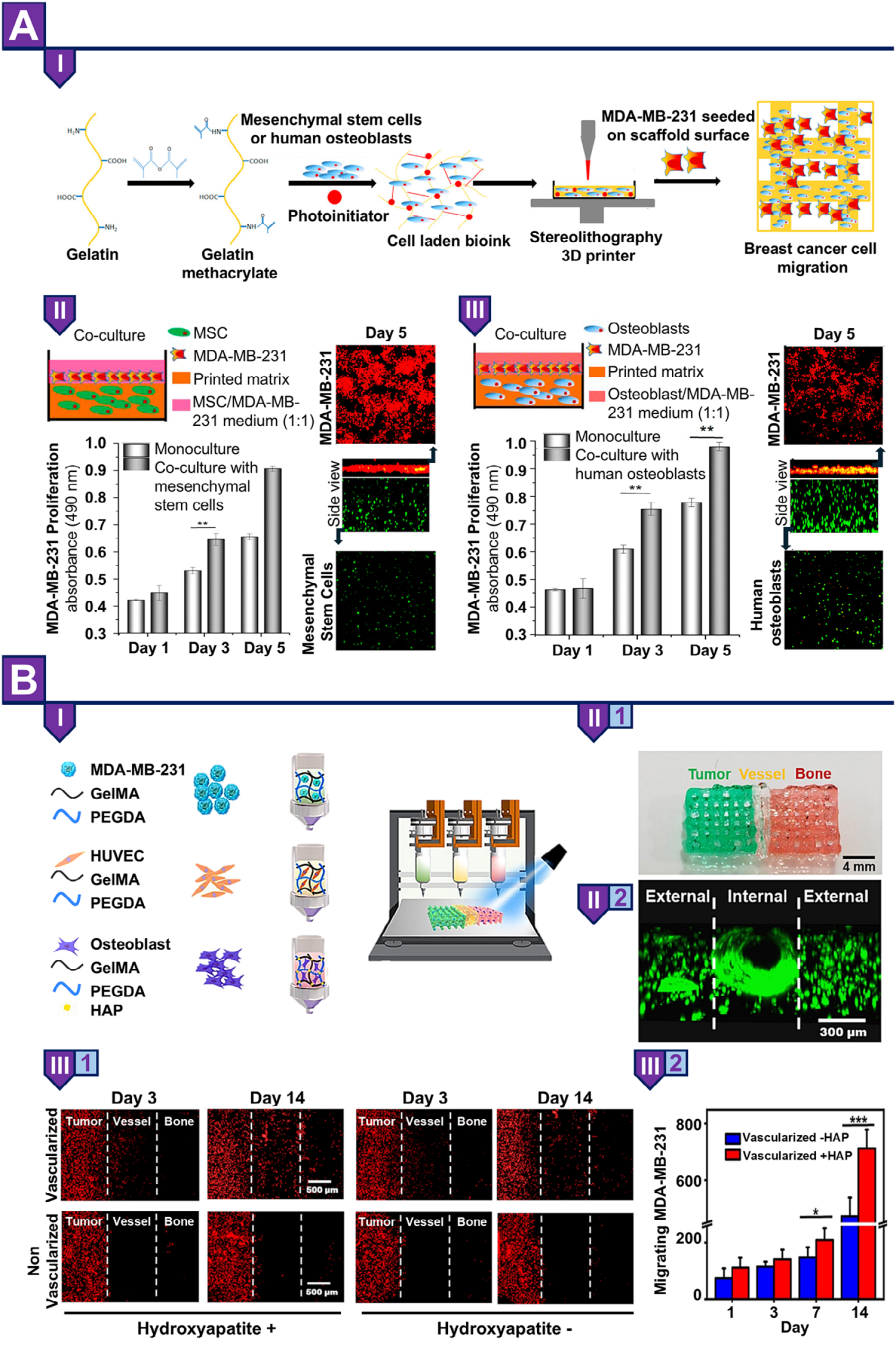
3DBP breast cancer metastasis models. (A) Breast cancer metastasis to bone. (I) Stereolithography‐based bioprinting of bone matrices composed of GelMA and nanocrystalline hydroxyapatite (nHA) to study the cross‐talk between human fetal osteoblasts, human bone marrow MSCs and MDA‐MB‐231 cells. (II) MDA‐MB‐231 cells mono‐ and co‐cultured with MSC in the 3DBP matrix composed of 15% GelMA+nHA(10 wt%) after 1, 3, and 5 days. Data are mean ± standard error of the mean; *n* = 6. The asterisk indicates *p* < 0.05, and two asterisks indicate *p* < 0.01. MSCs and MDA‐MB‐231 cells were stained with green dye and red dye, respectively. (III) MDA‐MB‐231 cells mono‐ and co‐cultured with osteoblasts in the 3DBP matrix composed of 15% GelMA+nHA (10 wt.%) after 1, 3, and 5 days. Data are mean ± standard error of the mean; *n* = 6. The asterisk indicates *p* < 0.05, and two asterisks indicate *p* < 0.01. Osteoblasts and MDA‐MB‐231 cells were stained with green dye and red dye, respectively. (B) Breast cancer metastasis to bone through vessels. (I) One‐step 3DBP of a tumor‐vessel‐bone co‐culture model for the reconstruction of the metastasis process of MDA‐MB‐231 cells to bone through a vessel. Three bioinks were used with precision to simulate the ECM of native tumor, blood vessel and bone tissues. GelMA, gelatin methacryloyl; PEGDA, poly(ethylene glycol) diacrylate; HUVECs, human umbilical vein endothelial cells; HAP, nano‐hydroxyapatite. II) 3DBP cell‐laden tumor‐vessel‐bone co‐culture model. (1) Visual appearance of the 3D model. (2) Distribution of green fluorescent protein‐tagged HUVECs in the vascular channel after 14 days of co‐culture. HUVECs grew adherently on the inner wall of the hollow tube, proliferated and spontaneously formed a dense arrangement. III) Migratory behavior of MDA‐MB‐231 in the co‐culture model over a 14‐d culture period. (1) Metastasis of MDA‐MB‐231 cells (red) in the vascularized and non‐vascularized co‐culture scaffolds with/without HAP, respectively. The model demonstrated the detachment of MDA‐MB‐231 cells from primary tumor tissue, migration through hollow blood vessels, and invasion and colonization in bone tissue. (2) The number of metastatic MDA‐MB‐231 cells in the vascularized scaffold with/without HAp during 14 days of co‐culture. The vascular environment generated by HUVECs and the presence of HAP significantly increased the number of MDA‐MB‐231 cells that migrated. Figure A reproduced with permission.[92] 2016, American Chemical Society. Figure B reproduced with permission.[93] Elsevier.

Functional vascular compartments were subsequently incorporated to investigate dissemination, transendothelial migration, and therapeutic response, as discussed in the following section.

#### 3.4.1 Vascularized Metastasis Models

Integration of an endothelial compartment allows metastatic models to progress beyond secondary‐site colonization toward local invasion, intravasation, transendothelial migration, and extravasation. However, endothelialized channels should not automatically be considered functional blood vessels. Static channels reproduce tumor–endothelial contact and route‐dependent migration, whereas perfusion additionally introduces transport, shear forces, and dynamic drug exposure. Available 3DBP breast cancer platforms therefore reproduce complementary segments, rather than the entirety, of hematogenous metastasis [93, 94, 201].

A stereolithography‐based tri‐compartment construct was fabricated from a tumor region containing either MDA‐MB‐231 or MCF‐7 cells, an intervening HUVEC‐laden GelMA channel, and a bone region containing human fetal osteoblasts within a GelMA/PEGDA/nHAp matrix [94]. The highly invasive MDA‐MB‐231 cells readily migrated across the vascular compartment and colonized the bone‐mimetic region, whereas MCF‐7 cells exhibited limited migration and expansion. Differences in endothelial‐marker expression were also observed between the two models, indicating that tumor phenotype influenced vascular maturation and progression. The platform therefore distinguished subtype‐dependent metastatic behavior within an identical spatial architecture.

A more integrated tumor–vessel–bone model was subsequently constructed using MDA‐MB‐231 cells, HUVECs, and human fetal osteoblasts in spatially distinct compartments (Figure 9B) [93]. The tumor and bone regions were printed using GelMA/PEGDA matrices, with nHAp incorporated into the bone bioink, while a central hollow channel was endothelialized after printing. During 14 days of co‐culture, HUVECs formed a dense lining within the channel, and cancer‐cell detachment from the tumor region was followed by migration through the vascular compartment and accumulation in the bone‐mimetic matrix. Both the endothelial environment and nHAp‐containing bone compartment increased cancer‐cell migration. However, the static vascular channel did not reproduce circulation.

Dynamic conditions were incorporated in a more recent bioprinting‐assisted hybrid platform in which heterotypic MDA‐MB‐231/normal human lung fibroblast spheroids were positioned within a bioprinted matrix containing an endothelialized perfusable microchannel [184]. Under perfusion, untreated cancer cells displayed pronounced directional migration toward and into the vascular lumen. Treatment with the multi‐receptor tyrosine kinase inhibitor TAS‐115 reduced migration, proliferation, and viability while increasing apoptosis and suppressing HGF/c‐Met and PI3K/Akt/mTOR signaling. These effects were further enhanced when TAS‐115 was combined with doxorubicin, with invasion being almost completely suppressed in the perfused model. By connecting molecular pharmacodynamic endpoints with a dynamic migratory response, this platform extended vascularized models toward anti‐metastatic drug evaluation.

Collectively, these models demonstrate a progression from static endothelial compartments toward perfused and spatially heterogeneous systems that link metastatic behavior with therapeutic response (Table 7). Nevertheless, most remain dependent on established cell lines and bone‐directed models, while metastasis to the lung, liver, or brain remains insufficiently represented.

**TABLE 7:** Summary of 3DBP breast cancer metastasis models.

Years	Cancer cells	Supporting cells	Bioink	Printing/Crosslinking	Model complexity/Stage	Time	Drugs	Endpoint assays	Advantages/Limitations	Refs.
2016	MDA‐MB‐231	hFOBs or hBM‐MSCs	GelMA (10%–15%) ± nHA	SLA + UV; tumor cells post‐seeded	Bone niche; colonization/remodeling; avascular hybrid	14 Days	—	Live/Dead; growth; imaging; VEGF; ALP	+Bone‐specific matrix; reciprocal crosstalk. −No vessel/immune cells; tumor cells post‐seeded.	[92]
2020	MDA‐MB‐231; MCF‐7	HUVECs; hFOBs	GelMA/PEGDA; vessel: GelMA; bone: +nHA	SLA + UV	Tumor–vessel–bone tri‐culture; migration/colonization; static	14 Days	—	Growth/Morphology; SEM/IF; tracked migration	+Subtype comparison in one architecture. −Static vessel; no immune/bone‐remodeling cells.	[93]
2023	MDA‐MB‐231	HUVECs; hFOBs	GelMA/PEGDA; bone: +nHAp	Multimaterial printing + UV; channel seeded post‐print	Tumor–vessel–bone; migration/colonization; drug test; static	14 Days	Paclitaxel	Viability; vessel lining; migration/colonization; drug response	+Links architecture to paclitaxel resistance. −Static vessel; no immune/bone‐remodeling cells.	[94]
2025	MDA‐MB‐231	NHLFs; HUVECs	Collagen I/fibrin (C2F3)	Aspiration placement; enzymatic gelation; seeded channel + perfusion	Tumor–lung‐fibroblast spheroid/vessel hybrid; invasion/intravasation under flow	3 Days	TAS‐115; doxorubicin; combination	Viability/IC_50_; apoptosis/Ki‐67; qPCR/luminex; invasion imaging	+Flow‐based anti‐migration test; pathway readouts. −Short‐term; no immune/patient‐derived cells.	[184]

Abbreviations: ALP, alkaline phosphatase; C2F3, collagen I (2 mg mL^−^^1^)/fibrin (3 mg mL^−^^1^) composite hydrogel; GelMA, gelatin methacryloyl; hBM‐MSCs, human bone marrow‐derived mesenchymal stem cells; hFOBs, human fetal osteoblasts; HUVECs, human umbilical vein endothelial cells; IC50, half‐maximal inhibitory concentration; IF, immunofluorescence; nHA/nHAp, nanohydroxyapatite; NHLFs, normal human lung fibroblasts; PEGDA, poly(ethylene glycol) diacrylate; VEGF, vascular endothelial growth factor.

### 3.5 Models Employing Preformed Spheroids

Preformed spheroids provide multicellular building blocks in which cell–cell contacts and tissue‐like architecture are established before printing (Figure 10). In these bioprinting‐assisted hybrid models, controlled spheroid deposition, spacing, and integration with hydrogels, dispersed cells, or vascular structures enable the rapid assembly of spatially organized constructs. Accordingly, models ranging from cancer‐only spheroids to multicellular tumor–stromal–vascular systems have been developed to investigate invasion, angiogenesis, therapeutic response, and tumor–immune interactions.

**FIGURE 10:**
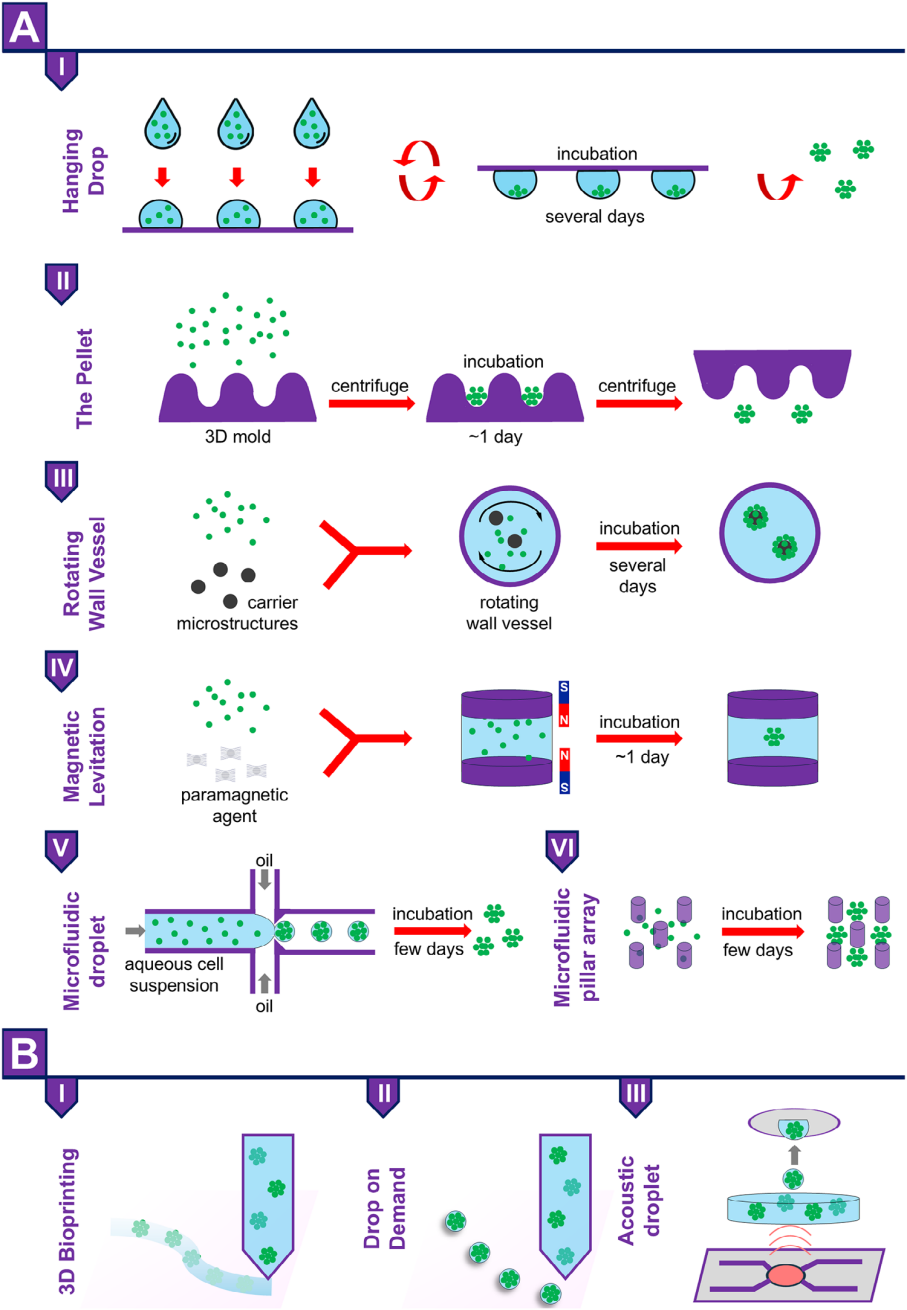
Spheroid and organoid fabrication and printing methods overview. (A) Spheroid and organoid fabrication methods. (I) The hanging drop. Droplets of a cell suspension are placed on support material that is inverted to face downward, causing the cells to collect at the bottom of each droplet under gravity. After incubation, the cells are obtained as spheroids or organoids. (II) The pellet. Three‐dimensional U‐shaped molds are used in a mild centrifugation process to pellet cells, which are then incubated for a relatively short time to obtain cell aggregates. (III) Rotating wall vessel. A device used to incubate cells under microgravity in a low‐turbulence, low‐shear environment to allow cells to form aggregates spontaneously or via the facilitation of microcarriers. (IV) Magnetic levitation. Paramagnetic drug‐internalized cells are accumulated at a certain height during their incubation under a magnetic field, thereby forming spheroids or organoids. (V) Microfluidic droplet. A typical microfluidic droplet generator with two inlets; one for an oil phase and one for an aqueous phase containing cells. Phases meet at a junction with a certain geometry to form aqueous droplets of desired size. Packed cells are then incubated to obtain cell aggregates. (VI) Microfluidic pillar array. Pillar arrays are constructed in a microfluidic chip, where incubated cells form aggregates which cannot attach to the surface due to surface treatment. As the aggregates grow, they are confined by the pillars, which allow aggregate size to be controlled. (B) Three‐dimensional spheroid and organoid printing methods. (I) Extrusion method. A wide range of hydrogel bioinks with shear‐thinning properties is used to fabricate well defined structures incorporating cell aggregates. (II) Drop on demand method. It is used to fabricate small structures with low‐viscosity bioinks. (III) Acoustic droplet. Another droplet‐based technology taking advantage of an acoustic field to generate and move droplets with milder shear stress.

#### 3.5.1 Cancer Cell Spheroids

To assess the utility of preformed spheroids, dispersed MCF‐10A, MCF‐10A‐NeuN, MDA‐MB‐231, and MCF‐7 cells were compared with preformed spheroids in Matrigel‐, gelatin–alginate‐, and collagen–alginate‐based bioinks [47]. Although dispersed cells remained viable after printing, spheroids developed only in Matrigel. By contrast, preformed spheroids retained >82% viability at 24 h and cell‐line‐specific morphology for at least 48 h in all three bioinks (Figure 11A). Pre‐assembly therefore reduced dependence on matrix‐supported post‐printing self‐organization, although a spacing of at least 100 µm was required to limit fusion into megaspheroids. Preformed spheroids were also more resistant to paclitaxel than dispersed cells.

**FIGURE 11:**
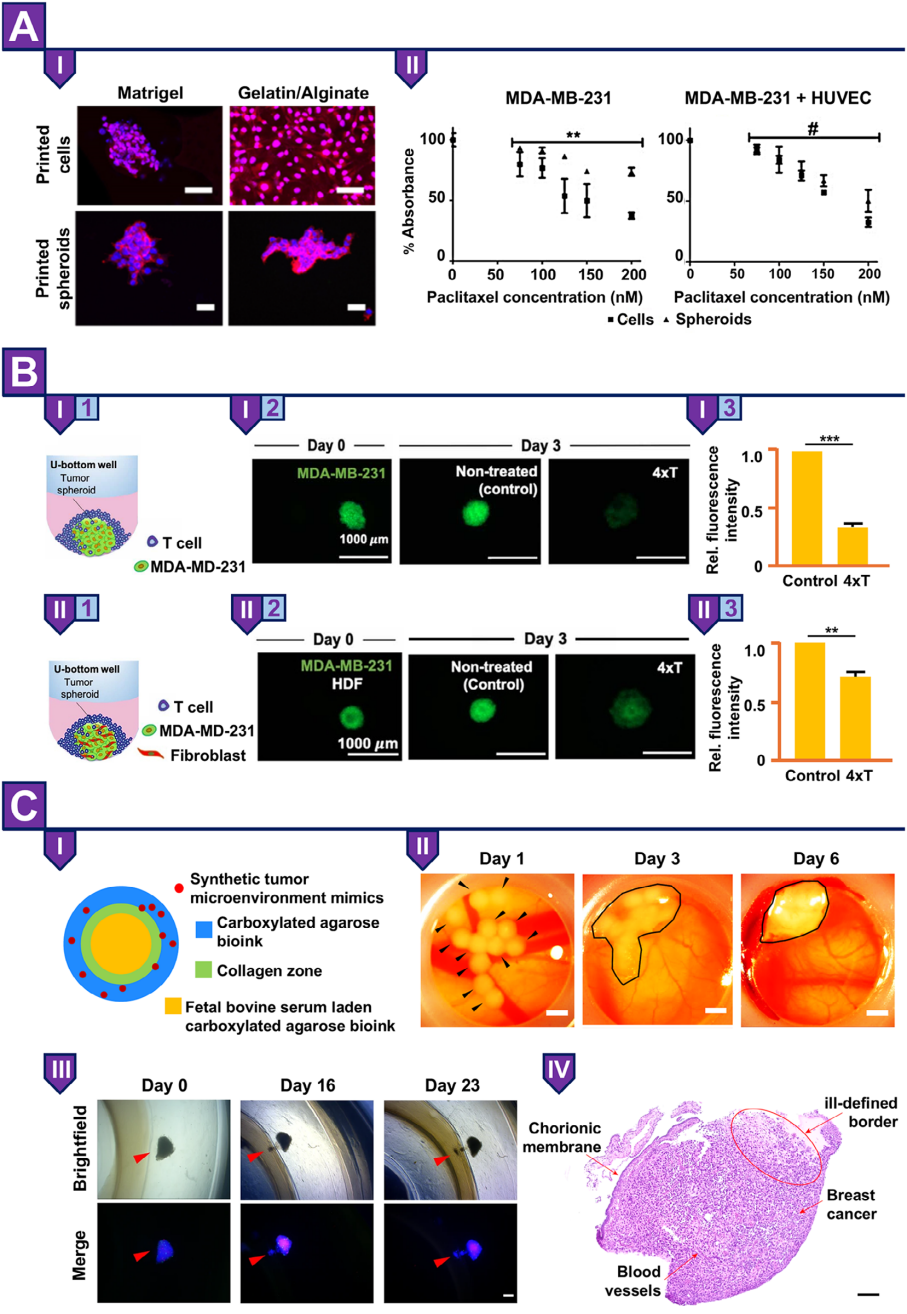
3DBP breast cancer models employing preformed spheroids. (A) Extrusion 3DBP with breast cancer cells and spheroids. (I) Confocal microscopy images of MDA‐MB‐231 cells and spheroids 5–8 days and 48 h, respectively, after bioprinting in Matrigel or gelatin/alginate (blue =Hoechst/nuclei and red = rhodamine phalloidin/actin). Scale bar 50 µm. While the printed cells could only form spheroids in Matrigel, the printed spheroids could retain their phenotype in both bioinks. (II) Bioprinted 3D breast spheroids were more resistant to paclitaxel than individual cells, but this effect was abrogated in co‐culture with endothelial cells. # *p* < 0.05, ** *p* < 0.001. (B) Preformed spheroids composed of breast cancer cells and fibroblasts. (I) Engineered T cell mediated cytotoxicity on homotypic MDA‐MB‐231 tumor spheroids. (1) A schematic image of the 3D model showing a free‐standing MDA‐MB‐231 spheroid treated with engineered T cells in a U‐bottom well plate. (2) Fluorescent images of MDA‐MB‐231 spheroids before (Day 0) and after (Day 3) the T cell treatment. T cell (effector) to cancer cell (target) ratio was 4:1 (4xT). (3) Graphical representation of fold change in GFP fluorescence intensity of homotypic spheroids as compared to the non‐treated control groups after the T cell treatment. (II) Engineered T cell mediated cytotoxicity on heterotypic free‐standing MDA‐MB‐231/human dermal fibroblast tumor spheroids. (1) A schematic image of the 3D model showing free‐standing MDA‐MB‐231/human dermal fibroblast spheroids treated with engineered T cells in U‐bottom wells. (2) Fluorescent images of MDA‐MB‐231/human dermal fibroblast spheroids before (Day 0) and after (Day 3) the T cell treatment. T cell (effector) to cancer cell (target) ratio was 4:1 (4xT). (3) Graphical representation of fold change in GFP intensity of heterotypic spheroids as compared to non‐treated control spheroids after the T cell treatment. Similar to the homotypic tumor spheroids, a decrease in GFP intensity was observed due to cytotoxicity. However, the extent of cancer cell death in the heterotypic tumor model was lower as compared to the homotypic model. The presence of fibroblasts partially abrogated the toxic effect on the cancer cells. (C) Recapitulating early cellular infiltration events in a chorioallantoic membrane tumor model. (I) Schematic on 3DBP invasion assay system comprising a construct of two conjoined concentric rings presenting the STEMs in a carboxylated agarose bioink, a collagen zone to mimic the basement membrane, encompassing a center laden with fetal bovine serum to establish a radial gradient of soluble signals. STEMs consisted of MDA‐MB‐231 cells, human primary microvascular endothelial cells and human bone‐marrow‐derived MSCs. (II) Optical micrographs showing the process of local invasion and coalescence of STEMs inoculated onto the chicken chorioallantoic membrane (CAM) into CAM tumors. Day 1: Black arrows point to STEMs on the CAM membrane. Day 3: Areas bound by a black line identify STEMs that are undergoing coalescence. Day 6: Areas bounded by the black line identify CAM tumors. Scale bar: 500 µm. (III) Images showing the process of formation of a tumor metastatic unit from STEMs and the invasion of the cell cluster (Day 16) into the collagen zone and the formation of a subsequent daughter cell cluster in the collagen (Day 23), providing insights into the potential formation of circulating tumor cell clusters. Red arrows point to the cell cluster. (IV) CAM tumor: H&E staining revealing a distinct small CAM tumor with clearly visible blood vessels and ill‐defined borders, morphologically reminiscent of a small breast cancer lesion. Scale bar 100 µm. Figure A reproduced with permission.[47] 2019, IOP Publishing. Figure B reproduced with permission.[50] 2022, IOP Publishing. Figure C was reproduced from ref. [53] (an open‐access article distributed under the terms of the Creative Commons Attribution License, CC BY 4.0).

An endothelial interface was subsequently created by depositing preformed MCF‐10A or MDA‐MB‐231 spheroids onto pre‐established HUVEC networks [48]. The spheroids and endothelial structures retained their morphology for at least 24 h, after which breast cells—particularly MDA‐MB‐231 cells—migrated from the spheroids along the endothelial network. Although the stable co‐culture period was short, and control over spheroid number and location was limited, this arrangement enabled direct tumor–endothelial interactions to be examined without waiting for post‐printing co‐assembly [48].

Greater analytical standardization was achieved through automated drop‐on‐demand deposition of individual MCF‐7 spheroids into poly(2‐hydroxyethyl methacrylate)‐coated electrochemical oxygen‐sensor microwells [49]. Spheroids were positioned with 25 µm precision while their integrity and viability (∼93%) were preserved. Label‐free monitoring quantified oxygen‐consumption rates of 450–850 fmol min^−^^1^ and detected an approximately 95% reduction following treatment with antimycin A, a mitochondrial inhibitor. The system therefore provided proof of concept for reproducible, real time metabolic phenotyping at the single‐spheroid level [49].

More recently, preformed MCF‐7 and MDA‐MB‐231 spheroids were incorporated into GelMA–PEGDA matrices of graded stiffness using a 96‐well‐compatible stereolithographic bioprinting platform [185]. After 7 days, matrix stiffness regulated MCF‐7 survival and metabolic activity and altered mechanotransduction‐ and EMT‐associated phenotypes, with distinct responses under standard and simulated microgravity. This work extends bioprinted spheroid models from structural preservation to mechanistic interrogation of matrix mechanics; however, no therapeutic response was evaluated.

#### 3.5.2 Cancer Cell and Fibroblast Spheroids

Preformed heterotypic spheroids containing GFP^+^ MDA‐MB‐231 cells and human dermal fibroblasts (HDFs) at a 2:1 ratio were used to examine how a fibroblast compartment modifies the response to engineered T cells [50]. Following priming with the MAIT‐cell ligand precursor 5‐ARU, CD8^+^ T cells expressing mucosal‐associated invariant T‐cell receptors induced effector‐density‐dependent tumor killing over 3 days (Figure 11B). At an effector‐to‐target ratio of 8:1, approximately 70% cancer‐cell death was observed in heterotypic spheroids, compared with 90%–95% in cancer‐only spheroids. CCL2 was detected only in the heterotypic cultures, consistent with altered cancer–fibroblast crosstalk.

To examine the spatial dependence of immune activity, engineered T cells were printed as circular patterns within type I collagen, and a preformed MDA‐MB‐231/HDF spheroid was aspiration‐deposited at the center, establishing tumor–immune separations of ∼250 or ∼650 µm [50]. Bioprinting controlled the geometry between the tumor and immune compartments. After 3 days, proximally positioned T cells reduced cancer‐cell invasion by approximately 70%, compared with 50% for distally positioned cells, and induced higher secretion of granzymes, IFN‐γ, and CCL2. In contrast, GFP intensity decreased similarly by ∼50%–60% at both distances, indicating that spatial proximity affected invasion and secretory signaling more clearly than the bulk tumor signal. The model nevertheless remained a short‐term static proof of concept.

More recently, preformed heterotypic spheroids containing GFP^+^ MDA‐MB‐231 cells and HDFs at a 1:2 ratio were used to evaluate RuNO_2_TPyP‐mediated photodynamic treatment in free‐standing and matrix‐embedded configurations [186]. Free‐standing spheroids showed concentration‐dependent photocytotoxicity (IC_50_ = 0.44 µM), increased caspase‐3/7 activity, and reduced Ki‐67 staining. Individual spheroids were then aspiration‐positioned at a uniform depth within collagen–fibrin matrices, providing a standardized extracellular‐matrix context. Treatment reduced spheroid diameter, GFP signal, and secretion of IL‐6, IL‐8, and HGF over 48 h.

#### 3.5.3 Cancer Cell, Fibroblast, and Endothelial Cell Spheroids

Preformed HUVEC/MDA‐MB‐231 spheroids were aspiration‐bioprinted into HDF‐laden fibrin at center‐to‐center separations of approximately 700–2500 µm [51]. Bioprinting enabled inter‐spheroid geometry to be varied while maintaining constant cellular composition and matrix conditions. After 5 days, cumulative endothelial sprout length peaked at approximately 1700 µm; smaller separations produced denser and more branched but shorter networks, whereas the response at 2500 µm resembled that of isolated spheroids. Tumor‐cell invasion did not vary significantly with spacing, indicating that mesoscale organization regulated collective angiogenesis more strongly than invasion. The model was static and nonperfused, and HDFs were dispersed within the surrounding matrix rather than incorporated into the spheroids.

Greater functional integration was achieved using tri‐cellular spheroids composed of HUVECs, MDA‐MB‐231 cells, and HDFs at a 7:1:1 ratio [52]. The spheroids were aspiration‐positioned within collagen–fibrin matrices at approximately 100 or 500 µm from a HUVEC‐lined perfusable channel. With a positional accuracy of approximately 50 µm, bioprinting established a controlled tumor–vessel interface. Under flow, proximal placement approximately doubled vascular length and junction number, promoted anastomosis with the channel, increased cancer‐cell spread, and enhanced vascular permeability relative to distal placement. Perfusion subsequently enabled doxorubicin and HER2‐targeted CAR‐T cells to be delivered through the endothelial channel. Doxorubicin produced dose‐dependent tumor reduction, whereas CAR‐T cells adhered to the endothelium, migrated through the vascular network, infiltrated the spheroids, and reduced tumor volume by ∼70% at the higher cell dose over 72 h [52]. Thus, the principal advance was the integration of precisely positioned spheroids with functional flow, permitting spatially controlled investigation of vascular drug delivery and immune‐cell trafficking.

#### 3.5.4 Cancer Cell, Mesenchymal Stromal Cell, and Endothelial Cell Spheroids

Preformed synthetic tumor microenvironment mimics (STEMs) containing MCF‐7 or MDA‐MB‐231 cells, human pulmonary microvascular endothelial cells (HPMECs), and bone marrow‐derived mesenchymal stromal cells (MSCs) at a 5:3:2 ratio were generated by hanging‐drop culture [53]. During 10 days, MSCs and endothelial cells remained closely associated, and lumen‐containing, PODXL^+^ endothelial structures formed in MDA‐MB‐231 STEMs but were absent when MSCs were omitted. MCF‐7 STEMs showed limited organization, while MCF‐10A controls developed only sparse endothelial structures. These interactions developed before printing and reflect the intrinsic cellular assembly within the spheroid building block. To investigate collective invasion, 10–15 preformed STEMs were extrusion‐printed within a nonremodelable carboxylated‐agarose outer ring surrounding an FBS‐laden agarose disk, with type I collagen subsequently introduced as an intervening barrier (Figure 11C) [53]. Printing therefore confined the spheroids to a defined annulus and established spatially distinct inert, remodelable, and soluble‐cue compartments. MDA‐MB‐231 STEMs generated traction on the collagen by Day 3 and subsequently disseminated cancer cells and occasional multicellular clusters into it, whereas MCF‐7 STEMs showed no collective migration. In a complementary ex ovo assay, 10–12 STEMs were placed in Matrigel on the chick chorioallantoic membrane rather than bioprinted [53]. After 6 days, coalesced tumors formed in 75% of MDA‐MB‐231 and 30% of MCF‐7 implants. MDA‐MB‐231 tumors contained human endothelial cell‐lined vessels with avian erythrocytes, consistent with host anastomosis, whereas MCF‐7 tumors were avascular and necrotic. Cancer‐only and binary cancer–MSC or cancer–endothelial spheroids dissociated by Day 3, supporting the importance of the complete tri‐cellular composition.

#### 3.5.5 Combination of Spheroids and Individual Cells

Hybrid models have combined preformed spheroids with dispersed cells to retain established multicellular organization while enabling stromal compartmentalization. In an early model, MCF‐10A or MDA‐MB‐231 spheroids were co‐printed with dispersed HUVECs within a six‐layer collagen–alginate grid [47]. Printing standardized their incorporation into a common 3D architecture but did not control spheroid–endothelial distance or vascular organization. Although the apparent paclitaxel resistance of preformed spheroids was attenuated in co‐culture, tumor‐specific sensitization could not be established because HUVECs were preferentially killed and the bulk metabolic assay did not resolve the two populations.

Greater spatial control was achieved in adipose‐stromal models. ASC spheroids were initially extrusion‐printed with homogeneous distribution in a thiolated‐HA matrix, differentiated into adipose microtissues, and integrated with a separately deposited layer of dispersed MDA‐MB‐231 cells [101]. More recently, preformed ASC or 9‐d differentiated adipose spheroids were printed as an HA‐based annulus surrounding a collagen‐I disk containing dispersed MDA‐MB‐231 cells [181]. This ring–disk geometry enabled live single‐cell tracking and showed that both stromal spheroid types increased tumor‐cell motility, persistence, and invasion distance. Bioprinting therefore progressed from simple co‐encapsulation to reproducible spatial separation of spheroid and single‐cell compartments. Nevertheless, the migration‐restrictive HA ring prevented cancer‐cell entry into the stromal compartment, and the outward directional bias was not significant; the model consequently quantified paracrine stimulation of migration rather than adipose‐directed invasion.

#### 3.5.6 Summary

Preformed spheroids reduce reliance on prolonged post‐printing self‐assembly by preserving established cell–cell contacts and tissue‐like organization before deposition. However, this biological complexity is generated during spheroid formation rather than by printing itself. Simple co‐encapsulation or transfer into grids and matrices mainly improves handling, reproducibility, and assay compatibility. A more substantive contribution is achieved when bioprinting controls spheroid spacing, tumor–immune separation, matrix compartmentalization, or tumor–vessel distance, or integrates spheroids with sensors and perfusable channels. These systems are therefore most appropriately regarded as bioprinting‐assisted hybrid models when printing establishes an experimentally meaningful architecture rather than merely dispensing spheroids (Table 8).

**TABLE 8:** Summary of 3DBP‐assisted breast cancer models employing preformed spheroids.

Years	Cancer cells	Spheroid/Added cells	Bioink	Printing/Crosslinking	Model design	Time	Treatment	Endpoint assays	Advantages/Limitations	Refs.
2019	MCF‐10A; MCF‐10A‐NeuN; MCF‐7; MDA‐MB‐231	Preformed epithelial/tumor spheroids; added: dispersed HUVECs in co‐culture arm	Matrigel; gelatin–alginate; collagen–alginate	Extrusion; six‐layer grid; ionic alginate or thermal crosslinking	Standardized spheroid deposition and HUVEC co‐printing.	72 h	Paclitaxel	Live/Dead; spheroid morphology and polarity; metabolic drug response	+Rapid, reproducible transfer. −Bulk readout.	[47]
2020	MCF‐10A; MDA‐MB‐231	Preformed breast spheroids; added: pre‐established HUVEC networks	Matrigel; gelatin–alginate; collagen–alginate	Direct extrusion onto endothelial networks; ionic or thermal crosslinking	Rapid spheroid–endothelial co‐culture assembly; spheroid position was controlled.	48 h	None	Live/Dead; epithelial polarity and architecture; endothelial‐network imaging	+Preserved viability and polarized architecture across bioinks. −No drug screening.	[48]
2022	MCF‐7	One preformed tumor spheroid; no supporting cells	PBS carrier; pHEMA‐coated sensor microwell (no ECM)	DoD PipeJet nanodispensing (22 nL); no crosslinking	Automated placement of one spheroid about 25 µm from an oxygen microelectrode in a sealable 55 nL well.	4 Days	Antimycin A	Live/Dead; deposition accuracy; amperometric O_2_; respiration rate	+Label‐free, rapid single‐spheroid metabolic monitoring. −Low biological complexity.	[49]
2025	MCF‐7; MDA‐MB‐231	Preformed 1000‐cell tumor spheroids; no supporting cells	GelMA/PEGDA with LAP; GelMA varied to tune stiffness	Vat photopolymerization using a custom SLA printer	Spheroids were embedded in stiffness‐tunable printed matrices and cultured at 1 g or simulated microgravity.	7 Days	Gravity	Metabolic activity; IF (Ki‐67, YAP and EMT markers); RNA‐seq	+Orthogonal control of matrix stiffness and gravity. −No stromal/vascular cells or therapeutic validation.	[185]
2022	MDA‐MB‐231	Spheroid: MDA‐MB‐231 ± HDF (2:1); added: MAIT‐TCR‐engineered CD8^+^ T cells	Fibrin; HDF‐laden fibrin matrix	Extrusion/aspiration‐assisted patterning; thrombin‐mediated fibrin gelation	Tumor spheroids and immune cells were patterned at proximal or distal spacing to test localization‐dependent killing.	3 Days	5‐ARU + engineered CD8^+^ T cells	Tumor viability and invasion; cytokines/chemokines; granzyme A/B	+Printing controlled tumor–immune distance and supported paracrine analysis. −Short culture.	[50]
2026	GFP^+^ MDA‐MB‐231	Heterotypic spheroid: MDA‐MB‐231:HDF = 1:2	Collagen–fibrin matrix	Aspiration‐assisted placement between matrix layers; thermal/enzymatic gelation	Uniform spheroid depth enabled comparison of free‐standing and matrix‐embedded photodynamic responses.	48 h	RuNO_2_TPyP	alamarBlue; GFP/diameter; caspase‐3/7; Ki‐67; IL‐6, IL‐8 and HGF	+Standardized ECM and light context. −No embedded‐model IC_50_.	[186]
2021	MDA‐MB‐231	Preformed HUVEC:MDA‐MB‐231 spheroids (9:1); added: HDFs dispersed in fibrin	HDF‐laden fibrin	Aspiration‐assisted spheroid placement; thrombin‐mediated fibrin gelation	Center‐to‐center spheroid spacing (about 700–2500 µm) was controlled to test collective angiogenesis.	5 Days	None	Endothelial sprouting/branching; invasion index; transcript profiling	+Isolated mesoscale proximity effects.	[51]
2022	MDA‐MB‐231	Tri‐cellular spheroid: HUVEC:MDA‐MB‐231:HDF = 7:1:1; HUVEC‐lined channel	Collagen–fibrin matrix	Aspiration‐assisted placement near a perfusable channel; enzymatic gelation	Tumor–vessel distance (about 100 vs. 500 µm) was controlled under flow for anastomosis and therapy delivery.	72 h	Doxorubicin; HER2‐targeted CAR‐T cells	Vascular length/Junctions and permeability; tumor volume; CAR‐T recruitment and infiltration	+Precisely defined tumor–vessel interface with perfusion.	[52]
2022	MCF‐7; MDA‐MB‐231	Preformed STEMs: cancer:HPMEC:BM‐MSC = 5:3:2	Carboxylated agarose; type I collagen; FBS–agarose source	Extrusion‐printed spheroid annulus; agarose gelation and collagen polymerization	A spheroid annulus, collagen barrier, and central serum source created distinct invasion compartments.	10 Days	None	IF (PODXL and Notch1); invasion imaging; CAM tumor formation and anastomosis	Defined inert, remodelable, and chemotactic regions. −Invasion was mainly qualitative.	[53]
2021	MDA‐MB‐231	Preformed ASC spheroids; added: dispersed cancer cells in a separately printed layer	HA‐SH/P(AGE‐co‐G) hydrogel	Extrusion; UV‐induced thiol–ene crosslinking	ASC spheroids were printed and differentiated for 21 days before a dispersed‐cancer‐cell layer was added for 9 days co‐culture.	30 Days total	Adipogenic differentiation	Live/Dead; triglycerides; adipogenic gene expression; ECM immunostaining	Long‐term adipose microtissue maturation and modular layering. −No therapeutic testing.	[101]
2024	MDA‐MB‐231	Preformed ASC or 9‐d adipose spheroids; dispersed cancer cells in collagen I	HA‐SH‐based stromal ink; collagen I tumor disk	Extrusion‐printed annulus; HA chemical crosslinking and collagen polymerization	A ring–disk architecture spatially separated stromal spheroids and tumor cells for live single‐cell migration tracking.	9 Days	None	Viability; lipid/ECM/Secretome; 3D tracking of motility, persistence, speed and invasion distance	Reproducible spatial compartments and cell‐resolved tracking.	[181]

Abbreviations: 5‐ARU, 5‐amino‐6‐D‐ribitylaminouracil; ASC, adipose‐derived stromal cell; BM‐MSC, bone marrow‐derived mesenchymal stromal cell; CAR‐T, chimeric antigen receptor T cell; DoD, drop‐on‐demand; ECM, extracellular matrix; FBS, fetal bovine serum; GelMA, gelatin methacryloyl; HA‐SH, thiolated hyaluronic acid; HDF, human dermal fibroblast; HGF, hepatocyte growth factor; HPMEC, human pulmonary microvascular endothelial cell; HUVEC, human umbilical vein endothelial cell; IF, immunofluorescence; LAP, lithium phenyl‐2,4,6‐trimethylbenzoylphosphinate; MAIT, mucosal‐associated invariant T cell; MSC, mesenchymal stromal cell; P(AGE‐co‐G), allyl‐functionalized poly(glycidol); PBS, phosphate‐buffered saline; PEGDA, poly(ethylene glycol) diacrylate; pHEMA, poly(2‐hydroxyethyl methacrylate); SLA, stereolithography; STEM, synthetic tumor microenvironment mimic; TNBC, triple‐negative breast cancer.

Collectively, the studies demonstrate that therapeutic and biological outcomes are highly configuration‐dependent. Drug response was influenced by the use of preformed rather than dispersed cells, matrix properties and embedding, stromal composition, and vascular integration. Fibroblasts modified immune‐mediated killing, while controlled spatial organization affected angiogenesis, invasion, and immune‐cell trafficking. Nevertheless, greater cellular complexity did not consistently produce more informative models: spacing affected angiogenesis more clearly than invasion in some systems, while bulk metabolic or fluorescence measurements frequently could not distinguish tumor‐specific effects from toxicity to supporting cells. Longitudinal cell‐resolved imaging, apoptosis and proliferation assays, secreted‐factor profiling, vascular measurements, and immune‐infiltration endpoints are therefore more informative when used in combination than single bulk‐viability readouts.

Most models remain based on MDA‐MB‐231 or MCF‐7 cells combined with HUVECs, dermal fibroblasts, or bone marrow‐derived MSCs. Cultures are generally short‐term and static, matrices are simplified, and intramatrix drug exposure, oxygenation, light penetration, and cell‐type‐specific responses are rarely quantified. Future development should combine standardized spheroid size and composition with patient‐derived cells, tissue‐matched stromal and vascular populations, broader immune components, and perfusion. Preformed spheroids thus provide a rapid and modular bridge between dispersed‐cell bioprinting and more complex organoid or organ‐on‐chip systems, but their added value depends on the extent to which printing controls spatial organization and functional integration.

### 3.6 Models Employing Preformed Organoids

Organoids are self‐organizing 3D structures generated from tissue‐derived stem, progenitor, or tumor cells that reproduce selected architectural and functional characteristics of their tissue of origin [202]. However, “organoid” and “spheroid” are sometimes used interchangeably in cancer‐modeling studies. In this review, organoid‐based models are restricted to constructs generated from primary tumor tissue, patient‐derived tumor cells, or patient‐derived xenograft tissue. Models are included only when bioprinting materially controls the matrix environment, spatial organization, or scalable manufacture of the construct, rather than functioning solely as a cell‐dispensing method (Table 9).

**TABLE 9:** Summary of 3DBP‐assisted preformed organoid‐ or microfluidic‐ or ex vivo tissue‐based breast cancer models.

Years	Cancer cells	Models	Matrix/Tissue	Printing/Crosslinking	Model design	Time	Treatment	Endpoint assays	Advantages/Limitations	Refs.
A. Models employing preformed organoids										
2023	C3(1)‐Tag murine breast tumor organoids	Preformed tumor organoids; no added cells	Matrigel; collagen I; CH; silk fibroin support bath	Embedded extrusion; thermal collagen gelation in a physical support bath	Organoid cores enabled matrix‐dependent morphology and phenotype comparison.	7 Days	None	H&E; morphology/area; IF (E‐cadherin, α‐SMA)	+CH best preserved source‐tumor‐like phenotype. −No added TME cells, or drug testing.	[42]
B. Combination of 3DBP and microfluidics										
2023	MCF‐7; MDA‐MB‐231; MCF‐10A	Dissociated cells in layered, mixed, or ratio‐controlled co‐cultures	Alginate–gelatin	Multi‐cartridge extrusion; ionic alginate crosslinking	Printing fixed initial cell ratios/positions; a tree‐like chip generated an EGF gradient.	11 Days; 8 h flow	EGF	Live/Dead; proliferation; cell‐resolved migration/chemotaxis	+Distinguished fabrication‐imposed position from migration. −Acute gradient exposure, no drug testing.	[54]
2023	BT‐474; HUVECs	Dissociated tumor/endothelial cells; self‐organized spheroids and barrier	Alginate–fibrinogen	Extrusion onto a micropillar chip; Ca^2^^+^/thrombin gelation	A 4 × 10 array with 25–150 µm pillar gaps tuned confinement and exchange under perfusion.	7 Days; 1 mL h^−^^1^	Pillar‐gap variation	Live/Dead; spheroid size; CD31^+^ endothelial‐barrier coverage	+Geometry‐controlled self‐organization. −No drug testing.	[55]
2024	BT‐474; HUVECs	BT‐474‐only, mixed, or concentric BT‐474/HUVEC models	RGD–alginate; RGD–alginate/fibrinogen	Multibarrel extrusion on a 4 × 9 micropillar chip; Ca^2^^+^ aerosol + thrombin	Thirty‐six models combined 3 TME compositions × 4 DOX concentrations.	7 Days perfusion	DOX	Live/Dead and IC_50_; spheroid morphology; IF; FITC–dextran and DOX transport	+Enabled multivariable barrier‐dependent screening.	[187]
2025	MCF‐7; MDA‐MB‐231; HUVECs	Preformed tumor spheroids; endothelial and bone‐mimetic compartments	ColMA/HAMA/collagen tumor inks; GelMA/HAp bone ink	Extrusion bioprinting; photopolymerization	Perfusable core/periphery–vessel–bone regions modeled invasion, intravasation, and colonization.	7 Days	DOX	Proliferation/Migration; IF (CD44, EMT markers); intravasation/extravasation imaging	+Connected metastatic stages in one chip. − No drug test in the integrated platform.	[188]
C. Bioprinting‐assisted models incorporating ex vivo tissues										
2015	MDA‐MB‐231; MCF‐7	BJ5ta fibroblasts; intact rat blood and lymphatic microvascular networks	Native rat mesentery; no hydrogel matrix	Laser direct‐write; 4 × 4 arrays or heterotypic patterns; no crosslinking	Defined cancer/fibroblast positions enabled cell‐resolved migration and vessel‐interaction tracking.	5 Days	None	Viability/Proliferation; migration/infiltration; vessel association and remodeling	+Retained intact blood/lymphatic networks. −Non‐mammary rat tissue, static culture, and no drug testing.	[189]
2021	4T1 (WT, non‐targeting, or CTSL‐KD)	Intact WT or NG2‐KO mouse mesometrium	2% alginate cell carrier; native mesometrium	Piezoelectric DoD inkjet; 10–12 droplets; no reported crosslinking	Cancer‐cell or host‐tissue genetic modification separated tumor‐ and TME‐derived effects.	5 Days	CTSL knockdown; host NG2 knockout	Live/Dead; time‐lapse proliferation/migration; sprouts, vascular islands and density	+Mechanistic tumor–host dissection in intact microvasculature. −Murine non‐mammary tissue, static culture, and no drug testing.	[87]
2025	MCF‐7; MDA‐MB‐231	Preformed spheroids; rat adipose microparticles with native stromal cells/ECM	FAT‐ink: 20% v/v adipose microparticles in collagen–alginate/gelatin/HA	Embedded extrusion; infiltrated or 0–2 mm cancer–adipose configurations	Controlled cancer–adipose distance revealed proximity‐dependent paracrine signaling and drug response.	7 Days	DOX; Compound C + SP600125; AG490; metformin	Live/Dead; proliferation/Migration; lipid/IF; adipokine ELISAs; efflux‐gene qPCR	+Preserved adipose complexity and spatial control. −Human cell lines with rat tissue, static culture.	[190]

Abbreviations: CH, collagen I–hyaluronic acid–poly(*N*‐isopropylacrylamide); ColMA, collagen methacrylate; CTSL, cathepsin L; DoD, drop‐on‐demand; DOX, doxorubicin; ECM, extracellular matrix; EGF, epidermal growth factor; ELISA, enzyme‐linked immunosorbent assay; EMT, epithelial–mesenchymal transition; FAT‐ink, fragmented adipose‐tissue ink; FITC, fluorescein isothiocyanate; GelMA, gelatin methacryloyl; H&E, hematoxylin and eosin; HA, hyaluronic acid; HAMA, hyaluronic acid methacrylate; HAp, hydroxyapatite; HUVEC, human umbilical vein endothelial cell; IF, immunofluorescence; KD, knockdown; NG2, neuron‐glial antigen 2; RGD, Arg‐Gly‐Asp; TME, tumor microenvironment; WT, wild type; α‐SMA, alpha‐smooth muscle actin.

#### 3.6.1 Animal‐Derived Cancer Cell Organoids

Preformed breast tumor organoids isolated from C3(1)‐Tag transgenic mouse tumors were incorporated into Matrigel, low‐concentration collagen I, or a collagen–hyaluronic acid–poly(*N*‐isopropylacrylamide) formulation (CH). The organoid‐laden hydrogels were deposited by embedded extrusion bioprinting into a physically crosslinked silk fibroin support bath (Figure 12) [42]. Distinct organoid behaviors were produced by the three matrices. Organoids printed in Matrigel or CH retained their spheroidal architecture between Days 1 and 7, whereas collagen alone induced early cell spreading and invasion into the surrounding matrix. Immunostaining further showed that the CH formulation most closely preserved the spatial distribution of E‐cadherin‐ and α‐smooth muscle actin‐positive populations observed in the source tumor. Matrigel supported little α‐smooth muscle actin expression, whereas collagen increased its expression but did not preserve the original organoid structure. Thus, the matrix was shown to function as an active experimental variable: the CH bioink favored phenotypic preservation, whereas collagen exposed a more invasive phenotype. Fabrication in multiwell plates also demonstrated the feasibility of parallel organoid production. Nevertheless, validation was limited to a 7‐day culture period and therapeutic response was not evaluated. Immune, vascular, and lymphatic components were also absent.

**FIGURE 12:**
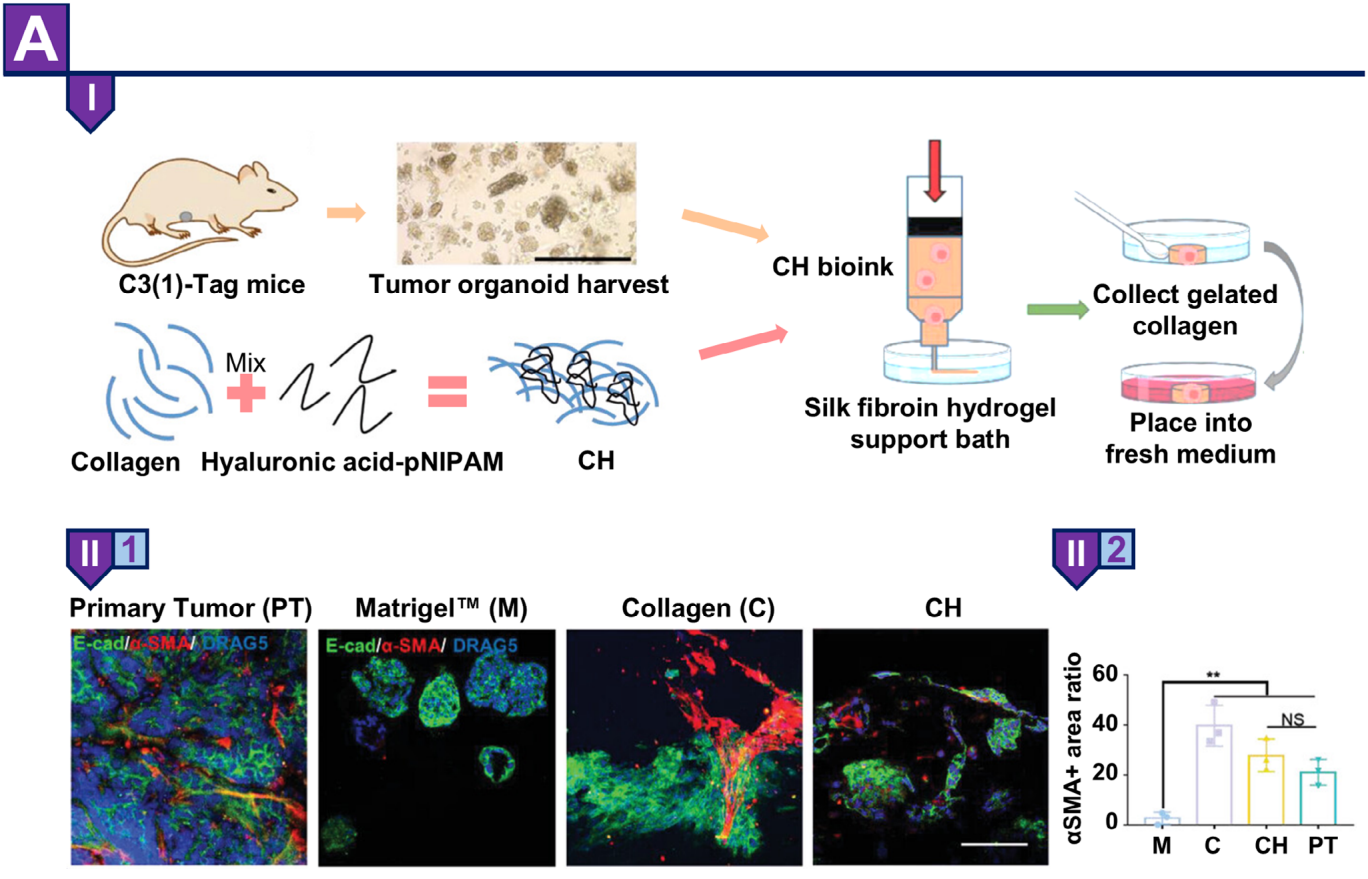
3DBP breast cancer models employing preformed organoids. (A) Effect of bioink composition on organoid phenotype maintenance. (I) Breast tumor organoids were harvested from C3(1)‐Tag transgenic mice tumors and mixed with collagen–hyaluronic acid–poly(*N*‐isopropylacrylamide) (pNIPAM) hydrogel precursor (CH), which was printed into a silk fibroin hydrogel‐based support bath at 37°C until complete gelation. (II) Immunofluorescence staining for E‐cadherin and α‐SMA proteins on mouse tumor sample and breast tumor organoids. Comparison of the organoid morphology and cell phenotype across different bioinks, namely, Matrigel, collagen, and CH, revealed that the bioprinted tumor organoids in the Matrigel and CH models remained in the spheroid structure from Day 1 to 7; however, in collagen, cells became more invasive and quickly spread to the surrounding area on Day 1, leading to the loss of the original spheroid structure. However, no obvious staining of α‐SMA was detected in the Matrigel model; in comparison, an increased level of α‐SMA expression was found in the collagen model. Only the CH model could resemble the primary tumor spatial organization and phenotype. Scale bar 100 µm. Figure A reproduced with permission.[42] 2023, John Wiley and Sons.

Future development of models employing preformed organoids should prioritize patient‐derived breast tumor organoids representing diverse molecular subtypes and intratumoral heterogeneity, together with longer‐term culture in compositionally defined bioinks. Integration of stromal, immune, vascular, and lymphatic components under dynamic perfusion will be required to reproduce clinically relevant microenvironmental interactions and drug transport.

### 3.7 Combination of 3DBP and Microfluidics

Microfluidic systems can complement 3DBP by superimposing biochemical gradients, perfusion, and microscale confinement on spatially patterned tumor constructs [203]. Moreover, coupling organ‐on‐a‐chip (OoC) systems with 3DBP supports the incorporation of real time sensing modules, allowing continuous monitoring of TME parameters and tumor response to different therapeutic molecules over time [204]. Recent advancements have demonstrated the utility of 3DBP‐based tumor‐on‐a‐chip models in replicating spatially defined TME architecture and dynamic conditions [202]. Breast‐specific applications have investigated controlled cellular positioning and chemotaxis, geometry‐dependent tumor–endothelial self‐organization, multivariable drug screening and multicompartment modeling of bone metastasis.

#### 3.7.1 Precise Control of the Printed Cell Positioning

An extrusion‐bioprinted tumor‐on‐chip model was developed using breast cancer cells encapsulated in an alginate–gelatin bioink [54]. Multi‐cartridge printing enabled MCF‐7 and MDA‐MB‐231 cells to be arranged either in successive layers or as mixed populations, thereby providing control over the initial spatial distribution and relative abundance of phenotypically distinct cancer cells. The optimized formulation maintained high post‐printing viability and supported cellular proliferation for up to 11 days. The printed constructs were subsequently integrated with a tree‐like microfluidic gradient generator containing two inlets and six outlets (Figure 13A). For chemotaxis analysis, MDA‐MB‐231 and non‐tumorigenic MCF‐10A cells were co‐printed at ratios of 1:1, 1:4, and 4:1 and exposed to a 100 nM epidermal growth factor gradient at a flow rate of 5 µL min^−^^1^ for 8 h. The greatest directional migration of MDA‐MB‐231 cells was observed at the 1:1 ratio, whereas migration was negligible at 1:4 and reduced at 4:1. In contrast, MCF‐10A cells showed little net migration. These findings demonstrated that the response to an identical biochemical gradient was influenced by the cellular composition of the construct.

**FIGURE 13:**
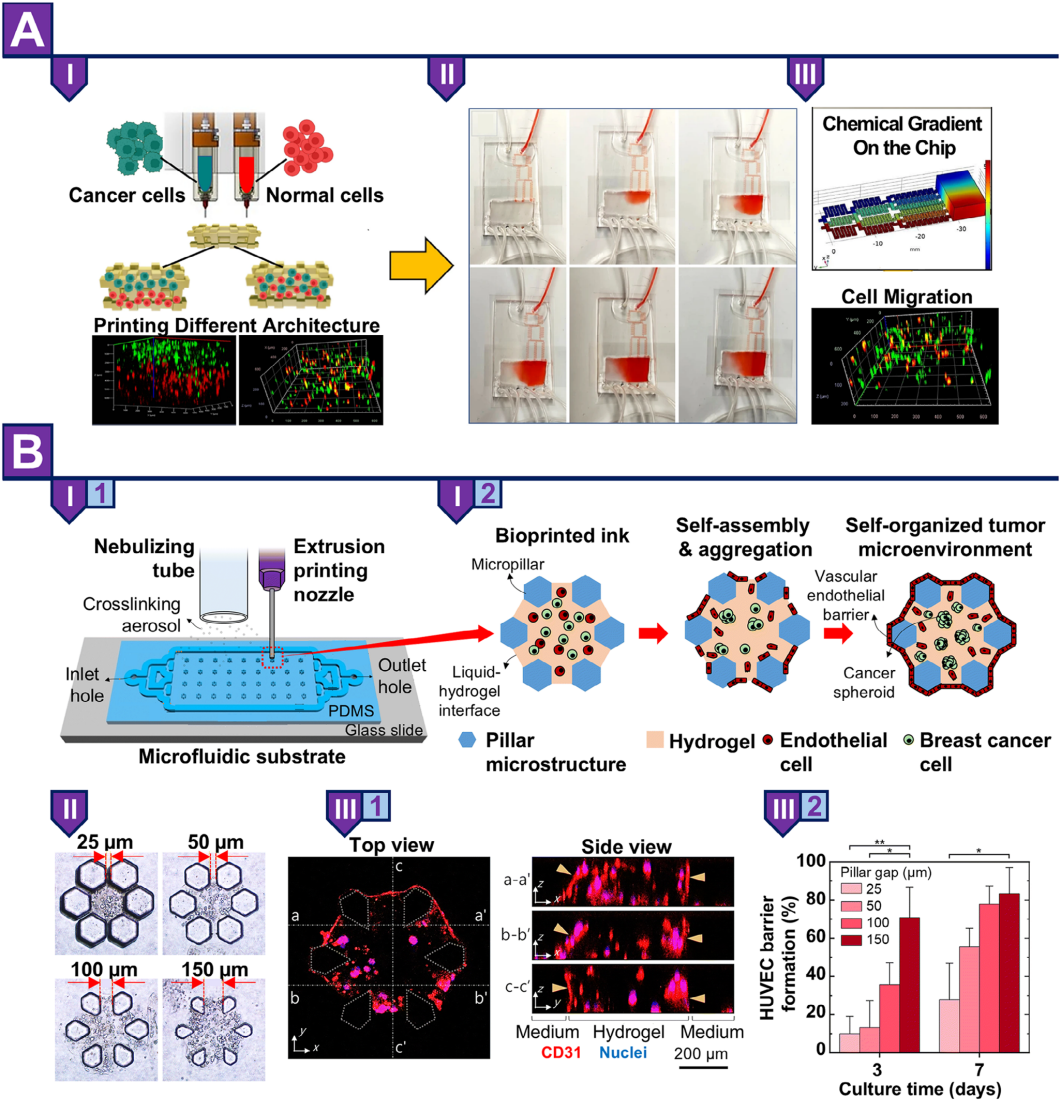
Integration of 3DBP and microfluidic devices to model breast cancer. (A) Modelling chemical gradients in TME with a microfluidic chip to investigate tumor heterogeneity. (I) An extrusion bioprinter was used to 3DBP breast cancer constructs, where tumorigenic breast cancer cells (MDA‐MB‐231 or MCF‐7) and non‐tumorigenic breast epithelial cells (MCF‐10A) in alginate–gelatin constructs could have distinct spatial distributions. (II) Pictures of the chamber at 5‐min intervals. The gradient was generated and maintained for at least 5 h without flow. The constructs were directly placed into the open chamber of a microfluidic tree‐like gradient generator, which was sealed afterward. (III) A gradient of epidermal growth factor as a chemoattractant was established in the tumor‐on‐chip model for 8 h to study migration patterns of MDA‐MB‐231 cells. The upper image shows a COMSOL simulation of the device, with red and blue representing higher and lower concentrations, respectively. The lower image shows representative MDA‐MB‐231 (green) and MCF‐10A (red) cell distribution after the gradient application. The test revealed that the migration of MDA‐MB‐231 cells depended on the ratio of tumorigenic to non‐tumorigenic cells in the construct, with the greatest migration observed at a 1:1 cell ratio, which might be attributed to the fact that a 1:1 ratio allows cell competition as compared to 1:4 or 4:1 cell ratios. (B) Modeling self‐organization of TMEs on microfluidic chips. (I) Schematic for a self‐organized TME array‐on‐a‐chip through bioprinting on a microfluidic substrate. (1) Direct bioprinting of the TME (TME) array on a microfluidic substrate. (2) Formation of the vascular barrier surrounding tumor spheroids in a TME unit, enabling self‐organization of the cancer cells and vascular endothelial cells using microstructures. (II) The bioinks trapped by micropillars with four different pillar gaps after bioprinting (BT‐474 cells and HUVECs suspended in an alginate–fibrinogen hydrogel precursor blend). (III) Self‐organization of the BT‐474 cells and HUVECs cultured in the bioprinted TME‐on‐a‐chip. (1) Immunostaining image showing vascular endothelial barriers, with CD31 (red) expressed by HUVECs. The side view images observed along lines a–a′, b–b′, and c–c′ of the top view image show the HUVEC barriers. The yellow arrows indicate the barriers. (2) Quantitative analysis of vascular‐barrier formation by HUVECs cultured in groups with various pillar gaps for 3 and 7 days. While the HUVECs cultured in the narrow groups with gaps of 25 µm and 50 µm were relatively slow to form the vascular barrier, the group with a 150 µm gap achieved 70% barrier coverage in 3 days. The significant differences between the pillar gap groups were analyzed using the Kruskal–Wallis test with a post hoc Dunn's multiple comparison test. * *p* < 0.05, ** *p* < 0.01, *** *p* < 0.001. Figure A was reproduced from ref. [54] (an open‐access article distributed under the terms of the Creative Commons Attribution License, CC BY 4.0). Figure B reproduced with permission.[55] 2023, Royal Society of Chemistry.

#### 3.7.2 Self‐Organization and Drug Screening

A self‐organized vascularized tumor array was fabricated by directly extrusion‐printing BT‐474 cells and human umbilical vein endothelial cells (HUVECs), suspended in an alginate–fibrinogen hydrogel precursor, onto a micropillar‐containing microfluidic substrate [55]. Printing generated repeated cell‐laden TME units, whereas the pillar arrays retained the hydrogel and defined the exchange pathways between the constructs and the perfusion channels. Following fabrication, BT‐474 cells self‐organized into tumor spheroids surrounded by CD31‐positive endothelial barriers. Interpillar gaps of 25, 50, 100, and 150 µm were evaluated to regulate mass transport within the printed arrays (Figure 13B). Wider gaps supported greater initial cell viability and more rapid cellular organization. Gaps of 100 µm favored the formation of larger tumor spheroids, whereas endothelial‐barrier development occurred more rapidly at 150 µm, reaching approximately 70% coverage by Day 3.

The platform was subsequently extended to integrate multiple model compositions and drug concentrations within a single microfluidic device [187]. Thirty‐six TME units were bioprinted in three configurations: BT‐474 monoculture, mixed BT‐474/HUVEC co‐culture, and spatially separated BT‐474/HUVEC co‐culture. Each configuration was evaluated at four doxorubicin concentrations. During seven days of culture, tumor spheroids and CD31‐positive endothelial barriers self‐organized within the co‐culture models, with the separated configuration producing the most continuous barrier around the tumor compartment. Reduced transport of 70 kDa FITC–dextran in the barrier‐containing models demonstrated that the spatial organization established by bioprinting subsequently influenced molecular permeability. Doxorubicin concentrations ranging from 0 to 1.5 µM were delivered through the microfluidic gradient generator for 24 h. The estimated IC_50_ increased from 0.82 µM in the BT‐474 monoculture to 1.34 µM in the mixed co‐culture. The resulting shift in drug sensitivity connected printing‐defined cellular organization and endothelial‐barrier formation with restricted drug transport and treatment response.

#### 3.7.3 Modeling Metastasis on a Chip

A multicompartment 3DBP microfluidic platform was constructed to reproduce selected stages of breast cancer bone metastasis using preformed MCF‐7 or MDA‐MB‐231 spheroids [188]. Spatially distinct tumor‐core and peripheral compartments were produced using collagen methacrylate–hyaluronic acid methacrylate–collagen hydrogels with tunable hyaluronic acid contents and stiffnesses. The softer core environment promoted early tumor‐cell proliferation, whereas the peripheral matrix supported outward migration. MDA‐MB‐231 cells migrated farther than MCF‐7 cells and maintained greater CD44 and vimentin expression together with lower E‐cadherin expression, consistent with their more mesenchymal phenotype. A HUVEC‐containing vascular‐like compartment was incorporated adjacent to the tumor region. Endothelial co‐culture increased vimentin and decreased E‐cadherin expression in MDA‐MB‐231 cells, while vascular endothelial growth factor promoted CD44 expression, tumor‐cell infiltration, and limited intravasation. A bone‐mimetic compartment composed of 8 wt% GelMA and 5 wt% hydroxyapatite was additionally printed to support the evaluation of extravasation from the vascular‐like region. Migration of MDA‐MB‐231 cells into this compartment demonstrated that the platform could connect tumor growth, vascular interaction, and colonization of a bone‐mimetic matrix within an independently perfusable system [188]. The system demonstrated a mechanistic model with potential for future antimetastatic drug testing.

#### 3.7.4 Summary

The available evidence remains limited to a small number of proof‐of‐concept studies based on established cell lines (Table 9). Culture periods were generally short, and therapeutic testing within the integrated devices was limited. Patient‐derived tumors, immune and lymphatic components, and resident bone cells also remain absent. Future platforms should incorporate subtype‐diverse patient‐derived models, physiologically relevant perfusion and oxygen gradients, and immune–vascular–lymphatic interactions. Standardized reporting of flow rate, shear stress, gradient stability, barrier permeability, drug exposure, and material‐dependent drug absorption will also be necessary. These developments should be combined with scalable fabrication, cell‐resolved response measurements, and validation before bioprinted microfluidic platforms can provide reliable high‐throughput drug screening.

### 3.8 Bioprinting‐Assisted Models Incorporating Ex Vivo Tissues

Native ECM, resident stromal populations, and pre‐existing vascular structures can be preserved by ex vivo tissues, thereby providing biological features that are difficult to reconstruct using cell‐laden hydrogels alone. When combined with 3DBP, cancer cells or spheroids can be deposited at defined positions while the harvested tissue supplies much of the underlying architecture. Breast‐specific applications have included the deposition of cancer cells onto intact mesenteric microvascular tissues and the incorporation of living adipose tissue into spatially organized tumor–stroma constructs (Table 9).

#### 3.8.1 Cancer Cells Printed Onto Intact Microvascular Tissues

Laser direct‐write printing was used to deposit MDA‐MB‐231 and MCF‐7 breast cancer cells and BJ5ta human dermal fibroblasts onto cultured rat mesentery (Figure 14A) [189]. This thin tissue, measuring approximately 20–40 µm, retained intact blood and lymphatic microvascular networks and permitted cell‐resolved imaging without tissue sectioning. Cancer cells were deposited as 4 × 4 arrays containing 16 spots separated by 0.8 mm, as single‐cell patterns, or as heterotypic MDA‐MB‐231/fibroblast and MDA‐MB‐231/MCF‐7 populations. Distinct migration patterns were observed following printing. MDA‐MB‐231 cells migrated distances equivalent to approximately 0.40 and 0.54 times the initial spot radius after 24 and 48 h, respectively, whereas MCF‐7 cells remained largely within their initial positions. In mixed MDA‐MB‐231/fibroblast constructs, fibroblasts preceded the cancer cells and accounted for approximately 84% of the cells outside the printed boundary after 24 h. In mixed MDA‐MB‐231/MCF‐7 constructs, MDA‐MB‐231 cells accounted for approximately 90% of the invasive population, while MCF‐7 cells remained localized. These results showed that controlled printing of heterogeneous populations could distinguish cell‐type‐specific and fibroblast‐associated migration within a native tissue environment. By Day 5, MDA‐MB‐231 cells had penetrated the thickness of the mesentery and were observed in association with endothelial cells and blood and lymphatic microvessels [189]. Vascularized regions beneath the printed cancer‐cell spots exhibited more than a twofold increase in capillary sprouts relative to unprinted regions. However, branch‐point number and total vascular length were not significantly different. The model therefore demonstrated localized endothelial sprouting and cancer–microvessel interactions.

**FIGURE 14:**
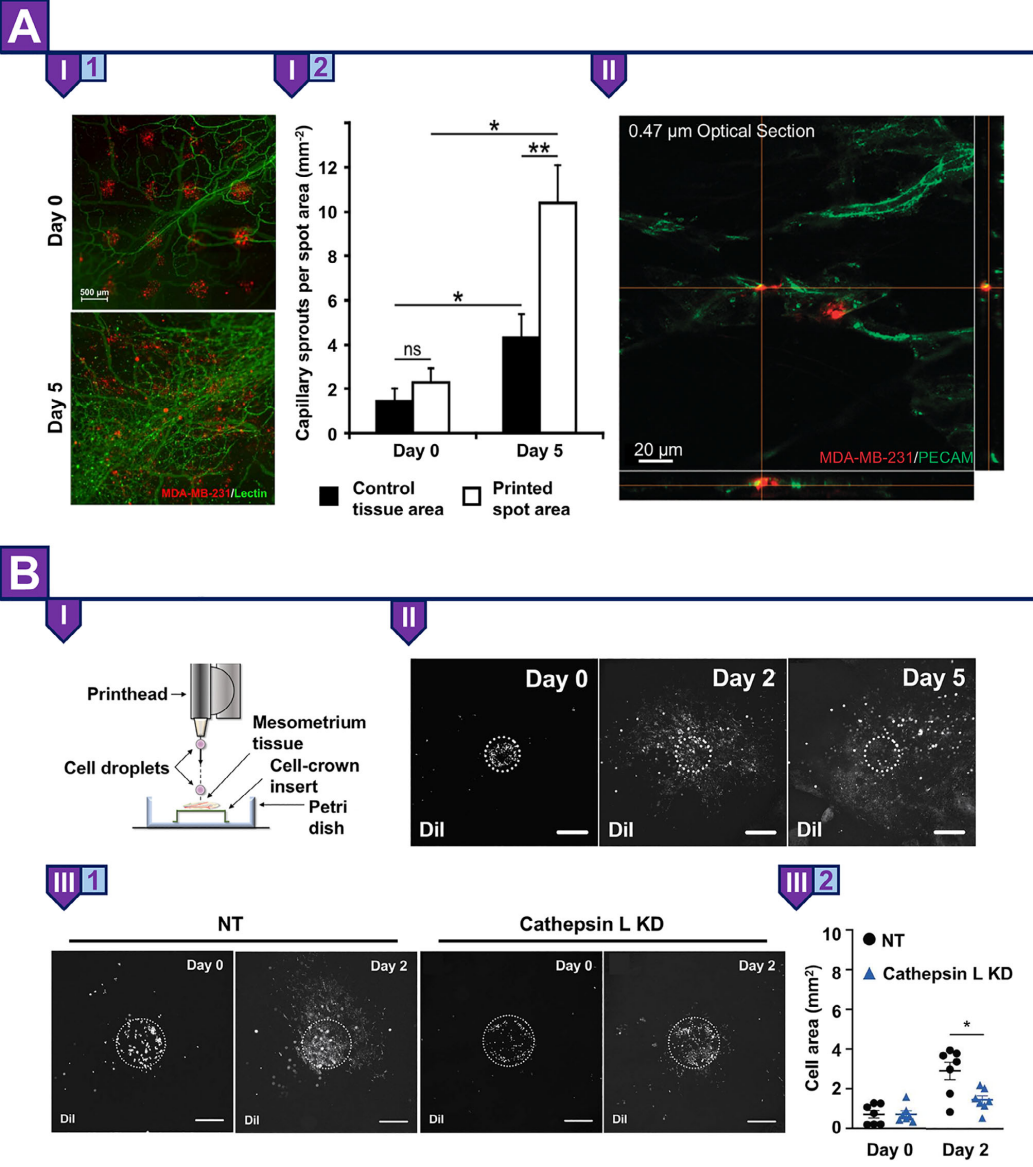
Modeling 3DBP breast cancer on ex vivo tissues. (A) Interactions between cancer cells and microvascular networks. I) Significant increase in capillary sprouting within groups with printed breast cancer cells compared to those without breast cancer cells. (1) Arrays of MDA‐MB‐231 cells printed on mesentery tissue and tracked over 5 days. By Day 5, evidence of angiogenesis in microvascular networks was observed in areas containing printed MDA‐MB‐231 cells. (2) Quantification of capillary sprouts, normalized to the initial‐spot area (*n* = 37 spots over 3 tissues, two‐way repeated measures ANOVA; * = *p* < 0.05, ** = *p* < 0.01, ns = not significant). II) Confocal microscopy images showing MDA‐MB‐231 cells interacting and integrating with lymphatic and blood vessels (PECAM‐positive endothelial cells). MDA‐MB‐231 cells were printed on cultured rat mesenteric tissues and infiltrated the tissue throughout 5 days to interact with endothelial cells. (B) Colonization of the cancer cell clusters with capillary sprouts. (I) Schematic of bioprinting exogenous cells onto live, ex vivo tissue. The cells were printed on top of the mesometrium tissue spread on a cell‐crown insert inside a 100‐mm Petri dish. (II) Bioprinting mouse breast cancer cells on live mouse tissue. DiI‐positive (fluorescently labeled) cancer cells were inkjet printed in a circular pattern (dashed circle). The printed cells could adhere to the underlying tissue, proliferate and migrate over 5 days. (III) Cathepsin L knockdown in breast cancer cells decreases proliferation and motility during ex vivo culture. (1) NT (non‐targeting) or cathepsin L knockdown (KD) 4T1 murine breast cancer cells were bioprinted on mesometrium tissues containing intact microvasculature with its native physiology. Scale bars = 500 µm. (2) Motility of bioprinted NT and cathepsin L‐KD cancer cells on mesometrium tissues was quantified during ex vivo culture. Cathepsin L‐knockdown cancer cells have decreased motility compared with NT cells after 1 day of culture in a physiologically relevant ex vivo tissue. Data are shown as the mean ± SEM; and * represents *p* < 0.05; *n* = 7. Figure A was reproduced from ref. [189] (an open‐access article distributed under the terms of the Creative Commons Attribution License, CC BY 4.0). Figure B reproduced.[87] 2021, Mary Ann Liebert, Inc.

A related platform was subsequently produced by depositing DiI‐labeled 4T1 murine breast cancer cells onto living mouse mesometrium using piezoelectric drop‐on‐demand inkjet printing [87]. The cell suspension was combined with 2% sodium alginate at a 2:1 ratio, and 10–12 droplets were deposited at one predefined position on each tissue. The printed cells remained viable and could be followed over 5 days to quantify proliferation and migration (Figure 14B). The platform was used to distinguish tumor‐intrinsic and host‐tissue contributions to cancer‐cell behavior. Knockdown of cathepsin L in 4T1 cells significantly reduced their proliferation and migration compared with non‐targeting controls. Independently, unmodified 4T1 cells printed onto mesometrium obtained from neuron‐glial antigen 2‐knockout mice showed lower proliferation and migration than cells printed onto wild‐type tissue. These experiments demonstrated that the same fabrication strategy could be combined with genetic modification of either the printed cancer cells or the host tissue to isolate specific tumor–microenvironment interactions.

Printed 4T1 clusters were found in close association with angiogenic microvessels [87]. Nevertheless, neither capillary‐sprout density nor total vascular density differed significantly between tissues containing printed cancer cells and sham controls. Only vascular‐island density increased significantly, from 0.32 to 1.14 islands mm^−^^1^. These findings indicate selective early microvascular remodeling rather than a generalized increase in angiogenesis.

#### 3.8.2 Breast Cancer‐Adipose Composite Tissue Models

A more breast‐relevant stromal environment was developed by micronizing living rat adipose tissue into particles approximately 280 µm in diameter and incorporating them at 20% v/v into a collagen–alginate formulation termed FAT‐ink [190]. The resulting material preserved native ECM together with heterogeneous populations of perilipin‐positive adipocytes, CD31‐positive endothelial cells, vimentin‐positive fibroblasts, and SSEA‐4‐positive adipose‐derived stromal cells. Embedded printing was then used to position MCF‐7 or MDA‐MB‐231 spheroids within the FAT‐ink or at defined distances of 0, 1, or 2 mm from the adipose compartment. Detailed interaction and treatment experiments were performed using MCF‐7 spheroids. Co‐culture induced cancer‐associated adipocyte‐like changes, including an approximately 29% reduction in adipocyte lipid‐droplet diameter, 2.3‐fold and 1.5‐fold increases in leptin and resistin secretion, respectively, and reduced adiponectin secretion. Cancer‐cell migration and proliferation were also increased, together with the expression of the drug‐efflux‐associated genes ABCB1, ABCC2, and ABCG2. These changes demonstrated reciprocal signaling in which cancer cells altered adipose‐tissue phenotype while the adipose compartment promoted a more migratory and treatment‐resistant cancer phenotype.

The effect of spatial proximity was evaluated during 6 days of doxorubicin exposure [190]. Cancer‐cell proliferation increased as the distance from the adipose compartment decreased, and the infiltrated configuration exhibited approximately 1.9‐fold greater proliferation than the 2‐mm configuration by Day 7. Distance‐dependent responses were also obtained using adipokine‐targeted interventions. Combined Compound C/SP600125 treatment, AG490, and metformin reduced proliferation by approximately 32%, 16%, and 23%, respectively, in the infiltrated configuration, whereas only the combined treatment produced a significant reduction of approximately 18% at a separation of 2 mm. The same therapeutic intervention could therefore produce different apparent efficacy depending on the spatial relationship between the cancer and adipose tissues.

Future studies should combine patient‐derived breast tumors with matched adipose and vascular tissues under long‐term physiological perfusion, together with the preservation or incorporation and functional characterization of immune and lymphatic components. These will be required to determine whether ex vivo tissue‐integrated platforms can provide reproducible drug‐screening systems.

## 4 Current Challenges and Future Outlook

### 4.1 Challenges

Across the model classes summarized in Table 3, the central challenge is no longer whether increasingly complex constructs can be fabricated, but whether the resulting models are fit for a clearly defined purpose. A reductionist construct may be appropriate for isolating tumor‐intrinsic or matrix‐dependent effects, whereas studies of metastasis or patient‐specific therapy prediction require validated stromal, vascular, lymphatic, immune, and transport functions. The origin of an observed phenotype or treatment response therefore cannot always be assigned with confidence, and increasing complexity does not automatically confer greater biological fidelity or predictive value [60, 61, 182].

Cellular representation and functional integration remain uneven. As summarized in Tables 4, 5, 6, 7, 8, 9, most platforms are dominated by established MDA‐MB‐231 or MCF‐7 cells, frequently combined with non‐matched endothelial, stromal, or immune populations. Patient‐derived breast tumor organoids have been bioprinted [89], but matched tumor, stromal, adipose, vascular, and immune tissues remain largely absent even from tissue‐integrated systems [190]. Moreover, cellular incorporation should not be equated with functional reconstruction. Endothelial marker expression, cord formation, or channel lining does not by itself establish lumen continuity, barrier function, or physiological perfusion; similarly, a lymphatic endothelial compartment does not demonstrate drainage, directional transport, permeability, or tumor‐cell intravasation [41, 199]. Immune competence cannot be inferred without defining immune‐cell survival, migration, subset identity, activation state, antigen presentation, cytokine signaling, cytotoxicity, and immune‐checkpoint activity. This distinction is particularly important because immune exposure can suppress tumor growth while simultaneously promoting vascular regression, desmoplasia, or invasion [183]. Adipose signaling is represented in only a limited number of systems, while neural and neuro–immune interactions remain largely absent despite their recognized roles in cancer progression [190, 205, 206].

Bioink stiffness, stress relaxation, degradability, porosity, ligand presentation, crosslinking chemistry, and matrix remodeling can alter proliferation, invasion, stemness, and treatment response [131, 140, 141, 172, 185]. The same properties also influence oxygen and nutrient transport, drug diffusion and partitioning, and optical penetration for imaging or phototherapies. Consequently, an apparently resistant phenotype may reflect restricted exposure rather than a cell‐intrinsic resistance mechanism. Matrigel provides complex biological cues but is murine tumor‐derived, compositionally undefined, and batch‐variable, whereas chemically defined synthetic matrices improve experimental control but require deliberate incorporation of relevant adhesion and degradation signals [130, 161]. Human tissue‐derived dECM can provide greater tissue specificity, but donor variability, processing‐dependent composition, and lot‐to‐lot mechanical differences complicate standardization [158, 159, 174, 177]. Interpretation is further weakened when matrix composition, stiffness, degradation, geometry, and cell density are altered together without matched acellular, monoculture, nonprinted 3D, and stiffness‐matched controls.

Printing resolution, shape fidelity, speed, cell survival, cell density, and bioink compatibility cannot generally be optimized independently [135]. Multimaterial, embedded, and microfluidic strategies expand the achievable architecture but introduce additional interfaces and failure modes, including incomplete channel formation or endothelialization, material mismatch, variable flow, and device‐dependent transport [170, 207]. Most breast cancer constructs summarized in Tables 4, 5, 6, 7, 8, 9 have been evaluated for no more than two to 3 weeks. Sustained operation requires matrix stability balanced against cell‐mediated remodeling, reliable nutrient and oxygen delivery, aseptic perfusion, and nondestructive longitudinal monitoring. Serial release and reprinting can extend cell propagation [102], but it does not reproduce continuous evolution within a mature TME. In flow‐based systems, differences in flow rate, shear stress, gradient stability, barrier permeability, tubing or device adsorption, and actual drug exposure further limit comparison between platforms [54, 55, 187, 188].

The drug‐screening evidence consequently remains difficult to compare and is not yet equivalent to predictive validation. Studies vary widely in the drugs selected, concentration range, exposure duration, treatment schedule, culture age, and analytical endpoint. Reported IC_50_ or EC_50_ values cannot be compared directly when cell number, proliferation rate, diffusion distance, matrix binding, oxygenation, and flow differ. Endpoint metabolic activity or bulk viability may also conflate tumor‐cell response with toxicity to stromal, vascular, or immune cells and may miss invasion, epithelial–mesenchymal transition or stemness. More complex models can therefore produce biologically informative but apparently discordant endpoints, as illustrated when immune‐mediated tumor suppression is accompanied by increased invasion [183]. Patient‐derived organoids, matrix‐controlled platforms, vascularized immunotherapy models, and multivariable arrays have expanded the available screening formats [52, 89, 172, 187], but they are still far from being established preclinical benchmarks.

High‐throughput fabrication and artificial‐intelligence‐assisted analysis are also constrained by the quality of the underlying measurements. Automated arrays, single‐organoid interferometry, integrated sensors, and spectroscopic monitoring can reduce destructive sampling and increase data density [49, 59, 176, 187]. However, most datasets remain small, single‐laboratory, and heterogeneous in imaging conditions, annotations, biological endpoints, and quality‐control criteria. Under these conditions, algorithms may capture batch, device, or imaging effects rather than tumor biology. Robust use of artificial intelligence therefore depends on standardized metadata, cell‐resolved ground truth, transparent preprocessing, external validation, and datasets sufficiently diverse to separate biological variation from manufacturing variation [60, 208].

Finally, reproducibility and qualification remain major translational barriers. Bioink formulation, rheology, cell source and passage, printing parameters, post‐print viability, construct geometry, culture conditions, flow, drug exposure, and endpoint definitions are reported inconsistently. Patient‐derived platforms add constraints related to tissue availability, processing time, consent, data governance, and the turnaround required for clinical decision‐making. Recent FDA initiatives promoting new approach methodologies and accepting an organ‐on‐chip technology into the ISTAND pilot signal growing regulatory openness, but they do not constitute qualification of 3DBP breast cancer models [62, 63]. Translation will require a predefined context of use, reproducible manufacturing, and prospective benchmarking against appropriate biological and clinical reference standards [61]. Until these requirements are met, the field will remain rich in innovative prototypes but limited in validated platforms suitable for routine drug development or patient‐specific treatment selection.

### 4.2 Future Outlook

The barriers identified above imply a modular development strategy. Figure 15 summarizes the historical progression of 3DBP breast cancer models, whereas Table 3 converts that evidence into class‐specific prospective roles. Cancer‐cell‐only and pairwise co‐culture systems should be standardized as scalable platforms; multicomponent, microfluidic, metastatic, and ex vivo tissue‐integrated systems should be developed as targeted modules for questions involving stromal regulation, transport, immune response, dissemination, or host‐tissue interactions [60, 61, 182].

**FIGURE 15:**
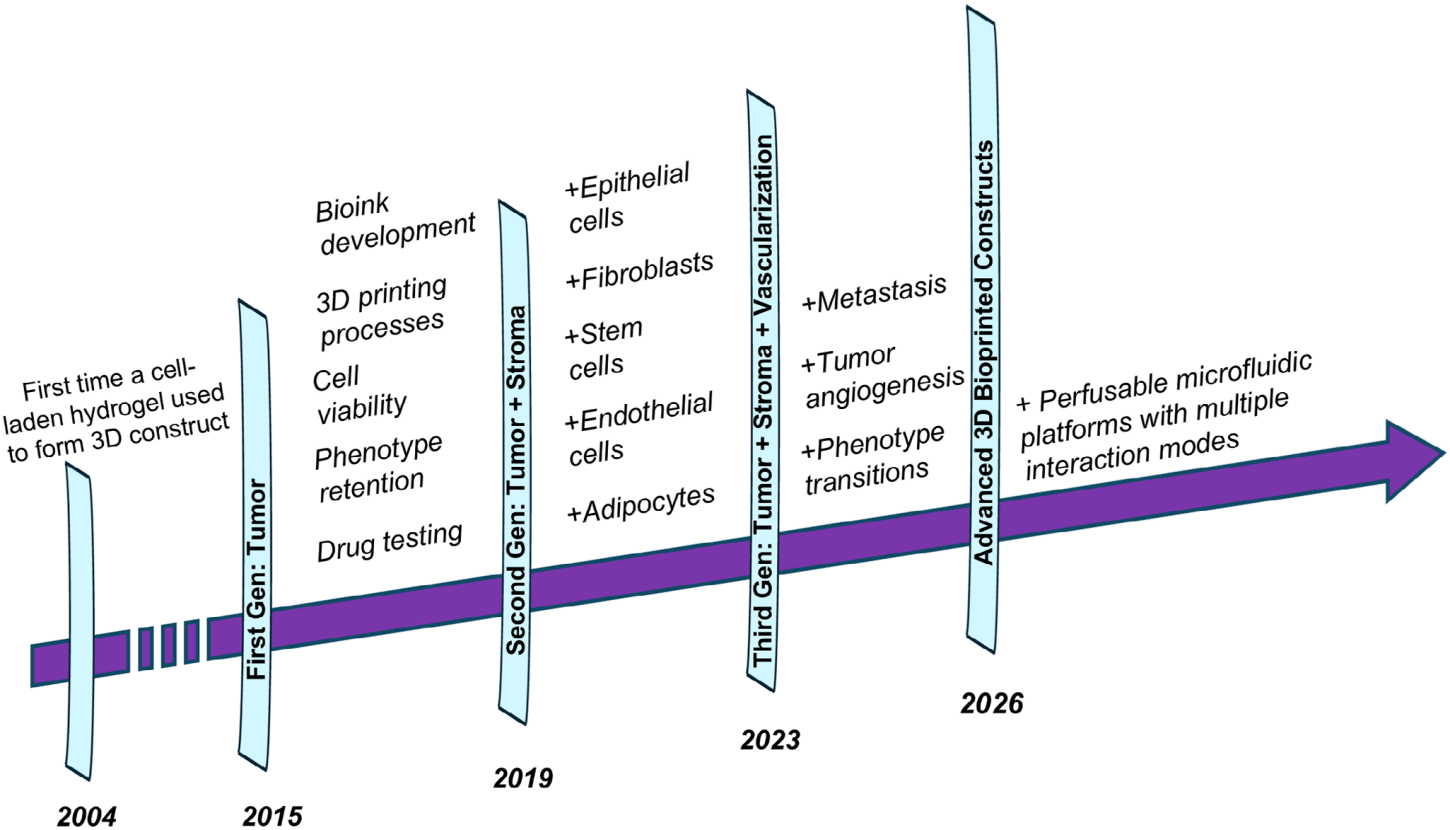
Evolution of 3DBP breast cancer models.

The most immediate biological advance should be a transition from a small set of established cell lines toward well‐characterized patient‐derived panels representing breast cancer subtypes, treatment histories, and interpatient variability [89]. Where tissue availability permits, tumor cells and organoids should be combined with matched fibroblasts, adipose tissue, vascular and lymphatic cells, and immune populations so that patient‐specific tumor–host interactions are retained rather than reconstructed from unrelated donors; current breast‐derived dECM and adipose models provide partial building blocks for this transition [177, 190]. This cellular transition should be accompanied by matrices whose biological composition and physical properties can be controlled independently. Human breast‐ or tumor‐derived dECM may provide tissue‐specific signals, but it should be processed with quantitative release criteria and, when necessary, combined with defined synthetic components to reduce donor and batch variability [158, 159, 174, 177]. Bioinks should consequently be selected not only for printability, but also for independently measurable stiffness, stress relaxation, ligand presentation, porosity, and cell‐mediated degradation, because these variables can modify invasion and therapy response [141, 172].

Functional validation will require blood vessels to be evaluated by lumen continuity, perfusion, permeability, and barrier response; lymphatic systems by drainage, directional transport, and tumor‐cell entry; and immune compartments by migration, activation, cytotoxicity, and checkpoint‐dependent function [41, 52, 183, 199]. Multimaterial and embedded printing can be combined with microfluidics to connect tumor, stromal, vascular, and tissue compartments while maintaining independently controlled matrices and flows [55, 170, 188, 207]. These connected systems are also expected to support sustained perfusion and longitudinal monitoring, allowing matrix remodeling, chronic treatment, immune adaptation, and dormancy to be observed within the same construct. In metastasis models, isolated bone‐mimetic endpoints are expected progressively to be complemented by linked primary‐tumor, vascular, and secondary‐niche compartments that reproduce sequential intravasation, circulation, extravasation, dormancy, and colonization under flow. Existing bone‐metastasis platforms provide an initial basis for this transition, whereas lung, liver, brain, and multiorgan routes are likely to remain priorities [61, 92, 93, 94, 188, 200]. Adipose or neural compartments will likewise be most informative when their incorporation is required by the biological hypothesis [190, 205, 206].

For drug development, a tiered testing strategy is likely to be more informative and scalable than reliance on a single maximally complex platform. Reproducible cancer‐cell‐only, spheroid, or array formats can first be used for dose‐range finding and broad compound prioritization; selected agents could then be examined in matrix‐controlled and multicellular systems to resolve stromal, vascular, immune, and transport effects; and the most informative conditions would finally progress to patient‐derived or tissue‐integrated models. Cell‐resolved viability, proliferation, apoptosis, invasion, stemness or epithelial–mesenchymal transition and immune activity are expected to be combined with longitudinal measurements rather than reduced to a single bulk metabolic endpoint. Automated spheroid placement, single‐organoid interferometry, integrated oxygen sensing, spectroscopic monitoring, and multicomposition arrays provide components of such a workflow [49, 59, 176, 187].

Future progress in automation and artificial intelligence is expected to depend on standardized experiments rather than heterogeneous proof‐of‐concept datasets. Automated printing, culture, dosing, imaging, and sampling could increase throughput while reducing operator‐dependent variation, provided that construct identity, cell provenance, bioink composition, printing parameters, culture age, flow, exposure, and quality‐control outcomes are captured as structured metadata. Artificial intelligence may then support image segmentation, multiparametric phenotype extraction, print‐parameter optimization, and integration of imaging, sensor, molecular, and treatment‐response data. Reliable model development will nevertheless require multicenter and sufficiently diverse training sets, cell‐resolved ground truth, locked preprocessing, interpretable outputs, and external validation so that biological signals can be distinguished from batch, device, or laboratory effects [60, 208].

Translation is expected to proceed through a staged qualification process. Near‐term priorities include establishing minimum reporting standards, reference materials, matched controls, and exposure‐normalized endpoint panels for each intended use. Platforms intended for patient‐specific prediction would then undergo prospective evaluation by comparing ex vivo responses with treatment outcomes. Sample requirements, fabrication failure rates, turnaround time, and cost will need to be evaluated alongside predictive performance, because a biologically sophisticated model that cannot operate within drug‐development or clinical timelines will have limited utility. Regulatory engagement is likely to be the most productive once the context of use and validation pathway have been defined [61]. Accordingly, near‐term success will be defined by reproducibility and interpretability, intermediate progress by functional integration and scalable validation, and ultimate success by demonstrated incremental value for drug development and individualized treatment selection.

## 5 Conclusion

The evidence synthesized in this review indicates that the evolution of 3DBP breast cancer models is best understood not as a linear progression toward maximal complexity, but as an expanding set of fit‐for‐purpose systems. Across directly bioprinted constructs and bioprinting‐assisted hybrid platforms, the distinctive contribution of printing has been the controlled deposition, spatial organization, architecture, and integration of tumor and host components. This control has enabled mechanistic investigation of matrix properties, cell identity and proximity, gradients, perfusion, and stromal, vascular, immune, and adipose interactions, all of which can modify invasion, angiogenesis, stemness, and therapeutic response. Nevertheless, greater structural or cellular complexity has not consistently produced greater biological fidelity or predictive value. Most systems remain short‐lived proof‐of‐concept models based on established cell lines, unmatched supporting cells, simplified matrices, and bulk endpoints. Drug responses are also highly dependent on geometry, transport, matrix composition, and exposure conditions and have rarely been benchmarked against patient outcomes.

Clinically useful platforms will therefore require modular escalation of complexity according to a predefined context of use. Reductionist models can serve as reproducible tools for early compound screening, whereas individualized prediction will require patient‐derived, preferably matched tumor and host tissues; functionally validated vascular, lymphatic, and immune compartments; sustained perfusion; and longitudinal, cell‐resolved measurements. Major biological gaps remain in immune competence, lymphatics, dynamic ECM remodeling, chronic treatment responses, and the sequential modeling of metastasis. Translation will further depend on standardized materials, fabrication, reporting, and drug‐exposure metrics; automation and AI‐ready data; interlaboratory reproducibility; and prospective comparison with established preclinical benchmarks. The next advance in the field will therefore be defined less by how many components a construct contains than by whether each added feature has a measurable function, the platform can be reproduced, and it provides incremental value for drug development or patient‐specific treatment selection.

## Author Contributions

**Mustafa Nakipoglu**: writing – original draft, writing – review and editing, visualization, methodology, data curation, and investigation. **Mahboobeh Mahmoodi**: investigation and writing – original draft. **Ehsanul Hoque Apu**: Writing – review and editing, and validation. **Fahimeh Shahabipour**: writing – review and editing. **Sibel Emir Diltemiz**: Writing – review and editing. **Sanjib Saha**: Writing – review and editing. **Halima Alem**: validation, and writing – review and editing. **Nureddin Ashammakhi**: conceptualization, supervision, writing – original draft, investigation, writing – review and editing, project administration, resources, and funding acquisition.

## Funding

N.A. acknowledges funding from the National Institutes of Health (1UG3TR003148‐01), the American Heart Association (AHA; 23IPA1053441), the Estonian Research Council (PRG1903), Michigan Translational Research and Commercialization Innovation Hub for AgBio (MTRAC AgBio, RG101700), Henry Ford Health, Department of Surgery (00679169), and the Corewell Health and Michigan State University Alliance grant.

## Conflicts of Interest

The authors declare no conflicts of interest.

## Use of Generative AI and AI‐Assisted Technologies in the Writing Process

ChatGPT Sol 5.6 (accessed July–August 2026) was used as assistive tool during manuscript revision. It supported editing and condensation of selected passages in the main text and particularly in comparison tables, as well as preparation of the graphical abstract. AI was not used to generate or analyze research data. All outputs were critically reviewed and revised by the authors; scientific claims, numerical details, citations, and table entries were verified against original full‐text publications and authoritative sources. The authors assume full responsibility for the final content.

## Supporting information




**Supporting File**: adhm71734‐sup‐0001‐SuppMat.pdf.

## Data Availability

Data sharing is not applicable to this article as no datasets were generated or analyzed during the current study.
