# Supplementary material for: Advances in 3D Bioprinting for Breast Cancer Modeling: Tumor Microenvironment, Metastasis, and Drug Screening
Source: Adv Healthc Mater. 2026 Oct 5;15(39):e71734. doi: 10.1002/adhm.71734 (PMC13691384; doi:10.1002/adhm.71734)
Supplement: Supplementary file 1 [file ADHM-15-0-s001.pdf]

Supplementary Information

Table S1. Comparison of recent reviews on 3D bioprinted breast cancer models.

Primary scope	Difference	Ref
All 3D breast-cancer platforms; 3D bioprinting is one modality	No breast 3DBP evolution.	[1]
Integration of breast organoids with 3D bioprinting	Microfluidic and metastatic applications are mainly conceptual	[2]
Breast-cancer bone metastasis and technology-to-clinic translation	Broad organoid/hybrid model evolution are not covered.	[3]
Breast-specific progression across directly bioprinted and bioprinting-assisted hybrid models	Breast-specific synthesis spanning cell sources, bioinks, cancer-only and multicellular models, vascular/lymphatic/immune niches, spheroids/organoids, microfluidics, metastasis, *ex vivo* constructs, drug screening.	Present review

References

1. Fröhlich E (2023) The Variety of 3D Breast Cancer Models for the Study of Tumor Physiology and Drug Screening. Int J Mol Sci 24:7116. https://doi.org/10.3390/ijms24087116

2. Shukla AK, Shukla S, Mongre RK, et al (2025) Integrating 3D Bioprinting with Organoid Technology-Based Breast Cancer Models for Drug Evaluation. Organoids 2025, Vol 4, Page 26 4:26. https://doi.org/10.3390/ORGANOIDS4040026

3. Miao L, Jiang D, Zhang X, Kang Y (2026) From Technological Innovation to Clinical Translation: Progress and Challenges in 3D Bioprinting for the Development of Breast Cancer Bone Metastasis Models. Adv Healthc Mater 15:e05819. https://doi.org/10.1002/adhm.202505819
